# deepmriprep: voxel-based morphometry preprocessing via deep neural networks

**DOI:** 10.1038/s43588-026-00953-7

**Published:** 2026-01-30

**Authors:** Lukas Fisch, Nils R. Winter, Janik Goltermann, Carlotta Barkhau, Daniel Emden, Jan Ernsting, Maximilian Konowski, Ramona Leenings, Tiana Borgers, Kira Flinkenflügel, Dominik Grotegerd, Anna Kraus, Elisabeth J. Leehr, Susanne Meinert, Frederike Stein, Lea Teutenberg, Florian Thomas-Odenthal, Paula Usemann, Marco Hermesdorf, Hamidreza Jamalabadi, Andreas Jansen, Igor Nenadić, Benjamin Straube, Tilo Kircher, Klaus Berger, Benjamin Risse, Udo Dannlowski, Tim Hahn

**Affiliations:** 1 Institute for Translational Psychiatry, University of Münster, Münster, Germany; 2 Department of Psychiatry and Neuroscience, Campus Benjamin Franklin, Charité-Universitätsmedizin Berlin, Berlin, Germany; 3 Institute for Geoinformatics, University of Münster, Münster, Germany; 4 Faculty of Mathematics and Computer Science, University of Münster, Münster, Germany; 5 Institute for Translational Neuroscience, University of Münster, Münster, Germany; 6 Department of Psychiatry and Psychotherapy, University of Marburg, Marburg, Germany; 7 Institute of Epidemiology and Social Medicine, University of Münster, Münster, Germany; 8 Department of Psychiatry, Medical School and University Medical Center OWL, Protestant Hospital of the Bethel Foundation, Bielefeld University, Bielefeld, Germany; 9 German Center for Mental Health (DZPG), Site Jena Magdeburg Halle, Berlin, Germany; 10 Center for Intervention and Research on Adaptive and Maladaptive Brain Circuits Underlying Mental Health (C-I-R-C), Site Jena Magdeburg Halle, Jena, Germany

**Keywords:** Computational neuroscience, Machine learning

## Abstract

Voxel-based morphometry (VBM), a popular approach in neuroimaging research, uses magnetic resonance imaging data to assess variations in the local density of brain tissue and to examine its associations with biological and psychometric variables. Here we present deepmriprep, a preprocessing pipeline designed to leverage neural networks to perform all the necessary preprocessing steps for the VBM analysis of T_1_-weighted magnetic resonance imaging. Utilizing the graphics processing unit, deepmriprep is 37 times faster than CAT12, the leading VBM preprocessing toolbox. The proposed method matches CAT12 in accuracy for tissue segmentation and image registration across more than 100 datasets and shows strong correlations in the VBM results. Tissue segmentation maps from deepmriprep have more than 95% agreement with ground-truth maps, and its nonlinear registration predicts smooth deformation fields comparable to CAT12. The high computational speed of deepmriprep enables rapid preprocessing of large datasets and opens the door to real-time applications.

## Abstract (web summary)

deepmriprep leverages neural networks to enable voxel-based morphometry preprocessing of MRI data that is 37× faster than existing methods while achieving comparable accuracy in segmentation, registration and final statistical maps across large datasets.

## Main

Voxel-based morphometry (VBM) is a widely used analytical approach in neuroimaging research that aims to measure differences in the local concentration of brain tissue across multiple brain magnetic resonance imaging (MRI) scans and to investigate their association with biological and psychometric variables^1,2^. Comparing neuroimaging data is challenging, because the intensity of MRI is not standardized, and brain structures differ across individuals. Standard VBM preprocessing addresses this by segmenting MRI scans into tissue classes and spatially normalizing the resulting tissue map to a template^3–8^. Finally, generalized linear models (GLMs) are fit for each voxel, modeling associations between spatially normalized tissue probabilities and the considered biological (for instance, age and sex) or psychometric variables (for instance, symptom severity or cognitive performance scores). If the GLM reveals a significant association, the corresponding voxel may be considered a region of interest (ROI), indicating a potential neural correlate of the variables under study.

Given that the effect sizes in VBM-based statistical analyses are typically small^9^, MRI datasets with thousands of participants are necessary to obtain accurate measurements^10^. To meet this demand, large-scale datasets have expanded to include more than 40,000 participants^11^, resulting in datasets that exceed 100,000 MRIs. However, preprocessing these large datasets with existing toolboxes, such as CAT12^7^, can take weeks or even months on standard hardware, delaying scientific progress. Developing a more computationally efficient VBM preprocessing pipeline could alleviate these processing bottlenecks, allowing researchers to focus on the conceptual aspects of their studies and accelerating scientific discovery. Therefore, creating a faster VBM preprocessing pipeline is a critical step forward for structural neuroimaging research.

In recent years, deep learning has emerged as a highly effective approach for various tasks in medical image analysis^12^, providing state-of-the-art performance in a wide range of image-related applications, such as semantic segmentation^13^. The neuroimaging community has followed this trend, resulting in neural network-based tools for brain extraction^14–16^, tissue segmentation^17,18^, registration^19–22^ and other neuroimaging-specific tasks^23^.

However, the adoption of neural network-based methods for preprocessing still lags behind classical toolboxes, such as CAT12^7^, SPM^3^ or FreeSurfer^6^, owing to two main reasons. First, deep learning tools often perform poorly in a realistic setting where they are applied to MRIs from scanner sites unseen during model training^14^. In line with ref. ^24^, this problem can be addressed by increasing the number of scanner sites^25^ and the extensive use of data augmentation^15,20,24,26^. Second, neural network-based tools are often specialized for only one processing step, while CAT12, SPM and FreeSurfer provide full processing pipelines for VBM and other methods such as surface-based morphometry (SBM). Tools, such as SynthMorph^24^, SynthStrip^15^ and EasyReg^21^, attempt to resolve this by being integrated into the FreeSurfer toolbox, serving as alternatives for parts of its preprocessing pipeline. However, to the best of our knowledge, there is no toolbox that has been developed from the ground up to fully harness the potential of neural networks across all preprocessing steps needed for VBM analysis.

We present deepmriprep, a preprocessing pipeline for VBM analysis of structural MRI data that is built to fully leverage deep learning. deepmriprep uses neural networks for the major VBM preprocessing steps: brain extraction, tissue segmentation and spatial registration with a template. Brain extraction is performed by deepbet^14^, the most accurate existing method to remove nonbrain voxels in T_1_-weighted (T_1_w) MRIs of healthy adults. To encompass the full VBM preprocessing, in this work, we additionally develop neural networks for tissue segmentation and registration. For tissue segmentation, we use a patch-based three-dimensional (3D) UNet approach, inspired by Isensee et al.^23^, which also exploits neuroanatomical properties, such as hemispheric symmetry. Nonlinear image registration is performed using a custom variant of SYMNet^22^, which uses a 3D UNet in conjunction with DARTEL shooting^27^ to predict smooth and invertible deformation fields.

The neural networks are trained on 685 MRIs compiled from 137 OpenNeuro datasets in a grouped cross-validation to ensure realistic validation performance. The worst predictions are visually inspected to identify potential weaknesses. In addition, deepmriprep is tested on 18 OpenNeuro datasets with pediatric healthy controls (HCs) and a dataset with synthetic atrophy and synthetic image artifacts. To investigate the effect of preprocessing on VBM-based statistical analyses, the VBM pipelines of deepmriprep and CAT12 are applied to 4,017 participants from three cohorts. In subsequent VBM analyses, associations with biological and psychometric variables are investigated, and the correlation of the resulting t-maps based on deepmriprep and CAT12 is analyzed. Finally, the correlations between deepmriprep- and CAT12-based tissue volume measurements are investigated in a ROI-based setting using the LPBA40 atlas^28^. In conclusion, our results indicate that deepmriprep is 37 times faster than CAT12 while achieving comparable accuracy in the individual preprocessing steps and strongly correlated results in the final VBM analysis.

## Results

### Tissue segmentation

#### OpenNeuro-HD

**Fig. 1:**
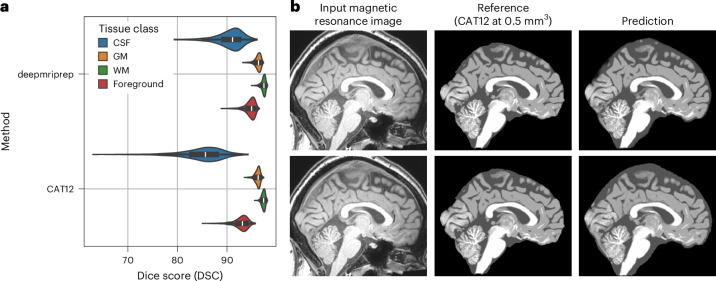
Dice scores of tissue segmentations of deepmriprep and CAT12 in OpenNeuro-HD with worst-case segmentation examples. **a**, Dice scores of deepmriprep and CAT12 with respect to the CSF, GM, WM and foreground (mean of CSF, GM and WM) across 685 MRI scans from OpenNeuro-HD. Violin plot density traces terminate exactly at the observed minima and maxima, and the superimposed box plots represent 25th percentile (lower), median (center) and 75th percentile (upper) with whiskers extending to data points within 1.5× interquartile range (IQR) of the quartiles. **b**, Sagittal slice of the T_1_w MRI, the reference tissue map (CAT12 at 0.5 mm^3^ resolution) and the predicted tissue segmentation map in the sample that resulted in the lowest foreground Dice score for deepmriprep (first row) and CAT12 (second row). Source data

We first evaluated deepmriprep and CAT12 on 685 high-resolution adult MRIs with strict quality control (OpenNeuro-HD) using cross-dataset validation. deepmriprep demonstrated robust tissue segmentation, achieving a median Dice score DSC_median_ of 95.0 across validation MRIs (Fig. 1a, Supplementary Fig. 1, Supplementary Table 1 (left) and Source Data Fig. 1). This high level of agreement with the ground truth—that is, CAT12 tissue segmentation maps derived from high-resolution MRIs (see ‘Preprocessing’ section in the Supplementary Information)—is an improvement compared with CAT12 (in original MRI resolution), which achieves a median Dice score DSC_median_ of 93.1.

**Fig. 2:**
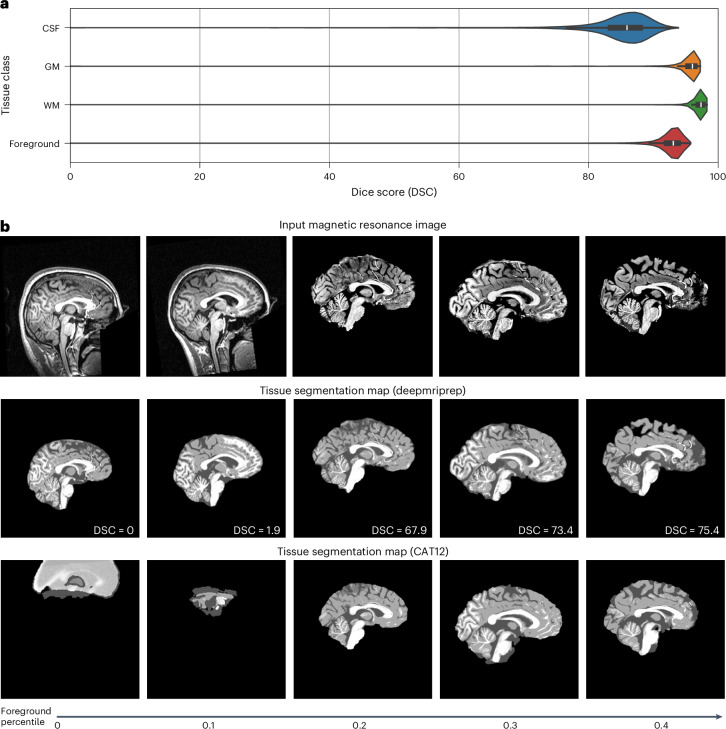
Dice scores of tissue segmentations of deepmriprep and CAT12 in OpenNeuro-Total with worst-case segmentation examples. **a**, Dice scores between deepmriprep and CAT12 across all 8,279 MRI scans from OpenNeuro with respect to the CSF, GM, WM and foreground (mean of CSF, GM and WM). Violin plot density traces terminate exactly at the observed minima and maxima, and the superimposed box plots represent 25th percentile (lower), median (center) and 75th percentile (upper) with whiskers extending to data points within 1.5× IQR of the quartiles. **b**, MRI input (first row) and tissue segmentation map (deepmriprep: second row, CAT12: third row) which resulted in the 0.0, 0.1, 0.2, 0.3 and 0.4 percentile foreground Dice scores across all 8,279 MRI scans. Source data

The high agreement of deepmriprep’s tissue segmentation with the ground truth in terms of the Dice score is confirmed by the foreground probabilistic Dice score (pDSC 84.7) and Jaccard score (JSC 90.6) shown in Supplementary Figs. 2 and 3 and Supplementary Tables 2 and 3. The segmentation of cerebrospinal fluid (CSF) resulted in the lowest median Dice scores ${\mathrm{DSC}}_{\mathrm{median}}^{\mathrm{CSF}}$ of 91.1 for deepmriprep and 85.6 for CAT12 (Fig. 2). Furthermore, the CSF Dice scores showed the strongest outliers across all 685 validation MRIs, with minimal Dice scores ${\mathrm{DSC}}_{\min }^{\mathrm{CSF}}$ of 73.6 for deepmriprep and 62.9 for CAT12. In the tissue maps that resulted in the minimal foreground metrics ${\mathrm{DSC}}_{\min }$, ${\mathrm{pDSC}}_{\min }$ and ${\mathrm{JSC}}_{\min }$ for each method (Fig. 1b and Supplementary Figs. 2 and 3), deepmriprep and CAT12 produced a thicker outer layer of CSF than the ground truth. With respect to gray matter (GM) and white matter (WM), the tissue maps of both methods did not show notable visual differences compared with the reference maps.

#### OpenNeuro-Total

To test robustness in a realistic, heterogeneous setting, we compared deepmriprep and CAT12 on 8,279 scans from 208 datasets with only minimal quality control (OpenNeuro-Total). Despite this challenging setting, the median Dice score DSC_median_ of 93.1 between deepmriprep and CAT12 tissue maps—not to be confused with Dice scores of each tool’s output compared with ground truths—showed high agreement for most of the respective tissue maps (Supplementary Fig. 4a). Again, GM and WM segmentation was most consistent with Dice scores of 96.0 for ${\mathrm{DSC}}_{\mathrm{median}}^{\mathrm{GM}}$ and 97.4 for ${\mathrm{DSC}}_{\mathrm{median}}^{\mathrm{WM}}$, while CSF segmentation resulted in a lower median Dice score of 85.9 for ${\mathrm{DSC}}_{\mathrm{median}}^{\mathrm{CSF}}$.

Despite the absence of ground truth, visually comparing tissue maps with low Dice scores—that is, low agreement between deepmriprep and CAT12—enables a qualitative assessment of each tool’s robustness.

Throughout the tissue maps with the 0.0th, 0.1th, 0.2th, 0.3th and 0.4th percentile foreground Dice scores DSC (Supplementary Fig. 4b), deepmriprep showed reasonable results with minor artifacts, while CAT12 was prone to errors. In the 0.0th and 0.1th percentile tissue maps, CAT12 produced unusable results with respective Dice scores of 0.0 for DSC^GWM^ and 1.9 for DSC^GWM^, compared with the reasonable tissue maps created by deepmriprep. In the 0.2th, 0.3th and 0.4th percentile tissue maps, CAT12 produced less detailed tissue maps than deepmriprep and misclassified tissue at the edge of the brain as background. deepmriprep properly classified the outer edge tissue, but misclassified areas of CSF as background in the 0.4th percentile tissue map. The same characteristic sources of errors could be found across the 16 tissue maps with the lowest agreement between deepmriprep and CAT12 (Supplementary Fig. 5), again measured by DSC. Finally, due to an error, CAT12 did not produce any tissue map for one MRI scan, while deepmriprep processed all 8,279 MRIs without any errors.

### Image registration

The registration of tissue probability maps with deepmriprep resulted in a median mean squared error MSE_median_ of 9.9 × 10^−3^ and a median linear elasticity LE_median_ of 250 during cross-dataset validation (Supplementary Figs. 6, left, and 7). These metrics indicate that deepmriprep performs on par with CAT12 (MSE_median_ 9.2 × 10^−3^, LE_median_ 240). While CAT12 showed slightly better median metrics, the supervised SYMNet used within deepmriprep resulted in a smaller maximal linear elasticity across MRIs, with an ${\mathrm{LE}}_{\max }$ of 366 (CAT12: ${\mathrm{LE}}_{\max }\,386$), and a smaller 95th percentile LE_95p_ of 280 (CAT12: LE_95p_ 283). This favorable linear elasticity indicates improved regularity of the deformation field for challenging probability maps—that is, maps that exhibit large voxel-wise differences from the template.

For both registration methods, the same probability map resulted in the largest voxel-wise mean squared error (MSE) after registration (Supplementary Fig. 6, right). Visual inspection of this warped probability map uncovers a small misalignment at the upper edge of the ventricles for deepmriprep, indicating less rigor in aligning the map with the template. Based on the absolute voxel-wise difference to the template, no apparent differences between deepmriprep and CAT12 could be found.

### VBM analyses

VBM analysis results for GM based on deepmriprep and CAT12 across all three datasets (Marburg-Münster Affective Disorders Cohort Study (MACS), Münster Neuroimaging Cohort (MNC) and BiDirect) demonstrated high similarity (Supplementary Fig. 8 and Supplementary Data 1), with strong correlation between the respective *t*-maps (Supplementary Table 7). The correlation of *t*-maps remained strong even for the psychometric variables—years of education, HC versus major depressive disorder (HC versus MDD) and intelligence quotient (IQ)—despite their smaller effects compared with the biological variables, namely age, sex and body mass index (BMI). The analyses that used all three datasets all resulted in correlation coefficients of *r* > 0.8, with BMI (*r* = 0.75) being the only exception. The equivalence between deepmriprep- and CAT12-based analysis outcomes is also supported by their similar maximal, absolute *t*-scores $| t{| }_{\max }$ (Supplementary Tables 8 and 9), especially for age and HC versus MDD. deepmriprep resulted in a larger maximal, absolute *t*-score for sex and age and smaller maximal, absolute *t*-scores for IQ, years of education and BMI. The difference in maximal values and the reduced *t*-map correlation for BMI was primarily driven by a large cluster in the outer cerebellum, which appeared only in the CAT12-preprocessed data (Supplementary Fig. 8).

The correlation coefficients of *r* > 0.8 also hold for the analyses using the MACS dataset and BiDirect dataset individually, again with BMI being the exception due to CAT12-based large clusters in the outer cerebellum (Supplementary Figs. 9 and 11). For analyses using only the MNC dataset, BMI-based *t*-maps strongly correlated with *r* = 0.83, while the sex-based *t*-maps resulted in a correlation coefficient of *r* = 0.72 (Supplementary Fig. 10).

The VBM results in WM (Supplementary Figs. 12–15) also exhibit strong correlations of *r* > 0.8 between most *t*-maps. Again, the BMI-based *t*-maps showed the lowest correlation caused by CAT12-based large clusters in the cerebellum. In addition, sex-based *t*-maps for the MNC dataset, BiDirect dataset and the pooled analysis showed lower correlation coefficients of 0.71, 0.71 and 0.73, respectively, and the HC versus MDD analysis for the BiDirect dataset resulted in a correlation coefficient of *r* = 0.74.

Finally, ROI-based GM volume measurements of deepmriprep and CAT12 exhibit strong correlations of *r* > 0.9 across all of the 29 ROIs of the LPBA40 atlas, with the only exception being the GM volumes of the brainstem region with a correlation of *r* = 0.75 (Supplementary Figs. 16–21). For WM, the caudate (*r* = 0.80), hippocampus (*r* = 0.81) and cerebellar lobe (*r* = 0.89) showed the lowest correlation coefficients, with all remaining regions resulting in correlations of *r* > 0.9.

### Processing time

deepmriprep achieved the highest processing speed on both low-end and high-end hardware (Supplementary Fig. 22) across the 8,279 scans from 208 datasets with only minimal quality control (OpenNeuro-Total). On high-end hardware, DeepMRIPrep took an average of 4.6 s per MRI using the graphics processing unit, whereas CAT12, parallelized across all 16 cores of the high-end processor, required an average of 173 s per MRI. On low-end hardware, deepmriprep and CAT12 take 209 s and 1,096 s per MRI, respectively.

## Discussion

We present deepmriprep, a neural network-based pipeline specifically built for VBM preprocessing. deepmriprep is 37× faster than CAT12, a leading toolbox known to be the more efficient preprocessing alternative to FreeSurfer for SBM. For preprocessing a large dataset containing 100,000 MRI scans such as the UK Biobank^11^, this translates into a reduction of computation time from 6 months to 5 days. While being faster, it delivers equivalent or better accuracy across tissue segmentation and registration. Most importantly, statistical maps based on deepmriprep preprocessing show strong correlations with respective CAT12-based VBM results.

It should be highlighted that assigning reliability to any statistical VBM map in the absence of gold standard or ground truth is inherently difficult^29^, aggravated by the large amounts of data required to achieve sufficient statistical power^10^. Therefore, the differences between the results of the deepmriprep- and CAT12-based VBM analyses should be interpreted with caution. Consequently, more research is needed to advance VBM from a scientific tool for detecting group-level differences to a reliable clinical application for individual patient diagnosis.

Furthermore, it should be noted that CAT12 is not solely a VBM toolbox but also offers SBM, while the current version of deepmriprep is limited to VBM, a shortcoming that should be addressed in a future version of deepmriprep to gain adoption in the broad neuroimaging community. Furthermore, the nonlinear registration could be improved by optimizing the affine matrix in conjunction with the warping field, thereby avoiding any potential biases of the initial affine registration. Finally, the training data quality could be improved to further increase deepmriprep’s accuracy. One promising, straightforward approach would be to use more training data with higher image quality, for instance, by using MRIs acquired with increased scan time, increased matrix size and reduced slice thickness.In addition, human expert annotations can be used to generate high-quality tissue segmentation maps and deformation fields, which can then be combined with lower-quality MRIs from the same session as input data. This would train the neural networks to predict high-quality tissue segmentation maps and deformation fields, even if the input MRI is of lower quality.

Although deepmriprep’s high processing speed and user-friendly interface are its main advantages, its underlying software design may hold even greater implications for future development (https://github.com/wwu-mmll/deepmriprep)^30^. The software is structured into small, modular components, each comprising fewer than 1,000 lines of code. This streamlined design improves long-term maintainability and reduces the likelihood of potentially far-reaching bugs^5,31^. Most importantly, the straightforward software architecture of deepmriprep reduces the barrier for researchers in VBM and other neuroimaging domains, making it easier to understand, adapt and reuse the code for various neuroimaging pipelines. We anticipate that the broader adoption of deepmriprep into other neuroimaging pipelines will advance the underlying methods, thus fostering progress in the broader neuroscience community.

## Methods

### Datasets

This study uses existing data from 225 datasets published on the OpenNeuro platform^32^ downloaded via the openneuro-py version 2023.1.0 Python package (https://github.com/hoechenberger/openneuro-py). OpenNeuro data from adult HCs were used for training and validation with cross-validation, while OpenNeuro data from HCs aged 2–12 years were used for testing, along with a Synthetic Atrophy dataset and three patient datasets: the Münster Neuroimaging Cohort (MNC), the Marburg-Münster Affective Disorders Cohort Study (FOR2107/MACS) and the BiDirect study. Data availability is governed by the respective consortia. No new data were acquired for this study.

#### Training and validation datasets

##### OpenNeuro-Total

Out of the over 700 datasets available at OpenNeuro at the time of compilation (10 November 2021), each dataset that contained at least five T_1_w MRIs from at least five adult HCs was included, resulting in 208 datasets. Based on a successive visual quality check, 30 MRIs were excluded, mainly due to improper masking (Supplementary Fig. 23a) and erroneous orientation (Supplementary Fig. 23b). The remaining compilation of 8,279 T_1_w MRIs is used as the OpenNeuro-Total dataset (Supplementary Fig. 24 and Supplementary Table 10).

##### OpenNeuro-HD

The 8,279 MRIs from OpenNeuro-Total were preprocessed using the commonly used CAT12 toolbox (https://neuro-jena.github.io/cat/) with default parameters^7^. To ensure high quality of the training data, strict quality thresholds were set based on the preprocessing quality ratings provided by the toolbox. To be included, all ratings had to be at least a B− grade, resulting in the following thresholds: a surface Euler number below 25, a surface defect area under 5.0, a surface intensity root mean square error below 0.1, and a surface position root mean square error below 1.0. All OpenNeuro datasets that contained fewer than ten adult HCs after this quality control were excluded. In the remaining datasets, MRIs were ranked according to the surface defect number, and finally the top five MRIs per dataset that passed a visual quality check were included in the dataset. This results in a total of 685 MRIs from 137 datasets called OpenNeuro-HD (Supplementary Fig. 24, middle, Supplementary Table 10 and Source Data Fig. 2).

#### Test datasets

##### OpenNeuro-Kids

Among the over 700 datasets available at OpenNeuro at the time of compilation (10 November 2021), each dataset that contained at least 5 T_1_w MRIs from at least 5 HCs in the age range from 2 to 12 years was included, resulting in 18 datasets. Based on a successive visual quality check, 300 MRIs were excluded, mainly due to strong motion artifacts (Supplementary Fig. 23c) and improper masking (Supplementary Fig. 23d). The remaining compilation of 867 T_1_w MRIs is used as the OpenNeuro-Kids dataset (Supplementary Fig. 24, right, Supplementary Table 10 and Source Data Fig. 1). The CAT12 preprocessing for OpenNeuro-Kids used the TMP_Age11.5 template.

##### Synthetic atrophy and synthetic artifacts

Published by Rusak et al.^33^, this dataset uses T_1_w MRIs of 20 HCs from the Alzheimer’s Disease Neuroimaging Initiative^34^ to synthetically introduce global neocortical atrophy. Simulating ten progressions of atrophy, ranging from 0.1 mm to 1 mm of global thickness reduction, the resulting dataset consists of 220 T_1_w MRIs (including the 20 originals) and their respective ground-truth tissue maps.

To additionally investigate the influence of scanner effects, we introduce artificial artifacts in the 20 original T_1_w MRIs using Rician noise, bias field, blurring, ghosting, motion, ringing and spike artifacts (Supplementary Fig. 25). Each of the seven artifacts is applied with medium and strong intensity, resulting in 480 synthetic MRIs.

##### VBM analysis datasets

For the VBM analyses, we use a total of 4,017 MRIs from three independent German cohorts (Supplementary Fig. 26): the Marburg-Münster Affective Disorders Cohort Study (MACS; *N* = 1,799), the Münster Neuroimaging Cohort (MNC; *N* = 1,194) and the BiDirect cohort (*N* = 1,024). All three cohorts include subsamples with both patients with MDD and HCs who are free from any lifetime mental disorder diagnoses according to DSM-IV criteria.

##### Marburg-Münster Affective Disorders Cohort Study (FOR2107/MACS)

Patients were recruited through psychiatric hospitals, while the control group was recruited via newspaper advertisements. Patients diagnosed with MDD showed varying levels of symptom severity and underwent various forms of treatment (inpatient, outpatient or none). The FOR2107/MACS was conducted at two scanning sites: University of Münster and University of Marburg. Inclusion criteria for the present study were availability of completed baseline MRI data with sufficient MRI quality. Further details about the structure of the FOR2107/MACS^35^ and MRI quality assurance protocol^36^ are provided elsewhere.

##### Münster Neuroimaging Cohort (MNC)

In MNC, patients were recruited from local psychiatric hospitals and underwent inpatient treatment due to a moderate or severe depressive disorder. Further information regarding this study can be found in refs. ^37,38^.

##### BiDirect

The BiDirect Study is a prospective project that comprises three distinct cohorts: patients hospitalized for an acute episode of major depression, patients up to 6 months after an acute cardiac event, and HCs randomly drawn from the population register of the city of Münster, Germany. Further details on the rationale, design and recruitment procedures of the BiDirect study have been described in refs. ^39,40^.

### Preprocessing

All datasets are preprocessed using the VBM pipeline of version 12.8.2 of the CAT12 toolbox, which was the latest version available at the time of analysis, with default parameters^7^. The affine transformation calculated during this initial CAT12 preprocessing is used such that tissue segmentation (see ‘Tissue segmentation’ section in the Methods) and image registration (see ‘Image registration’ section in the Methods) are consistently applied in the template coordinate space. Image registration is based on GM and WM probability maps in the standard resolution of 1.5 mm (113 × 137 × 113 voxels).

For tissue segmentation, unprocessed MRIs are affinely registered to the template in a high resolution of 0.5 mm (339 × 411 × 339 voxels) using B-spline interpolation, and the CAT12 preprocessing is repeated on the basis of these high-resolution MRIs. This circumvents any potential image degradation caused by additional resizing of the CAT12 tissue map. Because there exist no ground-truth tissue maps, these high-resolution tissue maps are used as reference maps for model training and validation. Because the MRIs are skull-stripped before tissue segmentation in deepmriprep’s prediction pipeline (see ‘Prediction pipeline’ section in the Methods), all voxels in the MRI that do not contain tissue in the respective tissue map are set to zero. Furthermore, the standard N4 bias correction^41^ is applied using the ANTS package^4^ to avoid interference with potential artificial bias fields introduced during data augmentation (see ‘Data augmentation’ section in the Methods). Finally, min–max scaling between the 0.5th and 99.5th percentile is used as proposed in ref. ^23^ with one modification: values above the maximum are not clipped to one but scaled via the function $f(x)=1+{\log }_{10}x$ to prevent any loss of crucial information in areas with extreme intensity values (for example, blood vessels). The code for the input preprocessing is publicly accessible at https://github.com/wwu-mmll/deepmriprep-train (ref. ^42^).

### Data augmentation

Data augmentation is used during training to artificially introduce image artifacts that may occur in real-world datasets. This increases model generalizability, because effects that are infrequent in the training data can be systematically oversampled with any desired intensity. Data augmentations for the image registration step would have to be consistent with equation (2), requiring specialized implementations. Hence, for the current version of deepmriprep, data augmentation is omitted during image registration model training.

The 12 different data augmentations used during model training (Supplementary Fig. 27) are implemented in the niftiai version 0.3.2 Python package (https://github.com/codingfisch/niftiai)^43^ and introduce artificial bias fields, motion artifacts, noise, blurring, ghosting, spike artifacts, downsampling, translation, flipping, brightness, contrast and Gibbs ringing. Bias fields are generated by applying an inverse Fourier transform to low-frequency Gaussian noise, whereas motion artifacts, ghosting^44^, spike artifacts^45^ and Gibbs ringing^46^ are achieved by introducing artifacts in the *k*-space of the T_1_w MRI. To be MRI-specific, noise is sampled out of a chi distribution^47^, a generalization of the Rician noise distribution^48^. Instead of using the full set of affine and nonlinear spatial transformations, only translation and flipping are applied via nearest-neighbor resampling to circumvent any potential image degradation.

### Tissue segmentation

To achieve high-quality tissue segmentation, a cascaded 3D UNet approach, inspired by Isensee et al.^23^, is applied to a cropped region of 336 × 384 × 336 voxels in the high-resolution MRI (see ‘Preprocessing’ section in the Methods). This specific cropping is chosen to make the image dimensions divisible by 16 (required by UNet), without excluding voxels which potentially contain tissue. The first stage of the cascaded UNet processes the whole image with a reduced resolution of 0.75 mm (224 × 256 × 224 voxels). In the second stage, the original resolution of 0.5 mm is processed using a patchwise approach, which incorporates the prediction from the first stage in its model input. For each patch position, an individual UNet is trained (see ‘Training procedure’ section in the Methods). Both stages of the UNet architecture are identical with respect to the use of the rectified linear unit (ReLU) activation function, instance normalization^49^, a depth of 4, and the doubling of the number of channels with increasing depth, starting with 8 channels. The implementation of the model and the training procedure is publicly accessible via GitHub at https://github.com/wwu-mmll/deepmriprep-train (ref. ^42^).

#### Patchwise UNet

The second stage of the cascaded UNet subdivides the 336 × 384 × 336 voxels of the high-resolution MRI into 27 patches, each containing 128 × 128 × 128 voxels (Supplementary Fig. 28). For each of these 27 patches, a specific UNet model is trained (see ‘Training procedure’ section in the Methods). To minimize the number of voxels in a patch that do not contain any tissue, the positions of the patches are optimized based on the tissue segmentation masks of the 685 MRIs in OpenNeuro-HD. This iterative optimization starts from a regular grid of 3 × 3 × 3 patches that covers the total volume. Then, each patch is moved stepwise by one voxel toward the image center until this would cause a tissue voxel in one of the 685 MRIs not to be covered by the patch. To exploit the brain’s bilateral symmetry, each of the patch on the left hemisphere is moved in lockstep with its corresponding patch on the right hemisphere.

Before applying patches from the right hemisphere to the UNet, we apply flipping along the sagittal axis such that they resemble left-hemisphere patches. The resulting prediction is then flipped back. This approach reduces the number of effective patch positions for which individual UNets need to be trained from 27 to 18.

Close to the border of a patch, the accuracy of the prediction typically decreases. Therefore, predictions close to the border of a patch are weighted less via Gaussian importance weighting^23^ during accumulation of the final prediction containing 336 × 384 × 336 voxels.

#### Multilevel activation function

Based on SPM^3^, the tissue maps produced by CAT12 contain continuous values ranging from 0 to 3. The values 1, 2 and 3 correspond to CSF, GM and WM, while 0 indicates that the respective voxel does not contain any tissue. The histogram of the template tissue map (Supplementary Fig. 29, right) shows that values close to 0, 1, 2 and 3 are more frequent than intermediate values. Furthermore, smaller peaks can be observed around the values 1.5 and 2.5, which correspond to the classes CSF–GM and GM–WM, respectively, which CAT12 introduces. To introduce an inductive bias toward this desired value distribution, the final layer of the tissue segmentation UNet utilizes a custom multilevel activation function inspired by Hu et al.^50^. This custom multilevel activation is achieved through the summation of six sigmoid functions,

$$f(x)=S(\alpha x)+\mathop{\sum }\limits_{i\in [1.5,2.,2.5,3.]}\frac{S(\alpha (x-i))}{2}\,\,\,\,{\mathrm{with}}\,\,\,\,S(x)=\frac{1}{1+{{\rm{e}}}^{-x}},$$

with *α* being a parameter of the neural network that is optimized during model training. This function (Supplementary Fig. 29, left) successfully maps a normal distribution—that is, the typical output distribution of a neural network—to the desired value distribution with peaks at 0, 1, 1.5, 2, 2.5 and 3 (Supplementary Fig. 29, middle). In combination with a MAE loss, this multilevel activation function facilitates the training of the tissue segmentation model.

#### Training procedure

The two stages of 3D UNets are trained in a cascaded fashion. The first stage model is trained for 60 epochs on full-view MRIs with a resolution of 0.75 mm (224 × 256 × 224 voxels) using a batch size of 1.

The training of the 3 × 3 × 3 patchwise approach in the second stage is more complex and results in 18 models, each dedicated to one of the 18 effective patch positions (3 × 3 × 3 = 27 minus the 9 flipped right hemisphere patches; see ‘Patchwise UNet’ section in the Methods). First, a UNet is trained on all effective patch positions for two epochs as a foundation model. For each of the 18 patch positions, this foundation model is finally fine-tuned, using solely the respective patch position for 20 epochs. For all patch-based training the batch size is set to 2, and the flip augmentation is disabled for patches located on the left and right hemispheres. The input of the second model consists of patches of the original MRI, concatenated with the respective patch of the first stage predictions upsampled to the image resolution of 0.5 mm. All models are trained with the one-cycle learning rate schedule^51^ using a maximal learning rate of 0.001, which follows the default settings of the fastai library^52^.

### Image registration

To introduce the neural network-based image registration used for deepmriprep, we first introduce the standard image registration approaches. In standard image registration such as DARTEL^27^, given an input image **I** and a template **J**, the sum of image dissimilarity *D* and a regularization metric *R* weighted with a regularization parameter *Λ*,

$$L({\bf{I}},{\bf{J}},{\boldsymbol{\Phi }})=D({\bf{I}}\cdot {\boldsymbol{\Phi }},{\bf{J}})+\Lambda R({\boldsymbol{\Phi }})$$

is minimized via the deformation field **Φ**. In the loss function *L* used in this standard approach, the regularization parameter *Λ* controls the trade-off between image similarity and the regularity of the deformation field. In CAT12, the default metric for image similarity is simply the MSE between the moving and the target image

$$D({\bf{I}}\cdot {\boldsymbol{\Phi }},{\bf{J}})=\mathrm{MSE}({\bf{I}}\cdot {\boldsymbol{\Phi }},{\bf{J}})=\frac{1}{| \varOmega | }\mathop{\sum }\limits_{{\bf{p}}\in \varOmega }| | {\bf{I}}\cdot {\boldsymbol{\Phi }}({\bf{p}})-{\bf{J}}({\bf{p}})| {| }^{2},$$

and the regularization term is the linear elasticity of the deformation field **Φ**

1 $$R({\boldsymbol{\Phi }})=\int \left(\mu \parallel \epsilon ({\boldsymbol{\Phi }}){\parallel }^{2}+\frac{\lambda }{2}{(\mathrm{tr}(\epsilon ({\boldsymbol{\Phi }})))}^{2}\right){\rm{d}}{\bf{x}},$$

where *μ* is the weight of the zoom elasticity and *λ* is the weight of the shearing elasticity.

To guarantee that the deformations are invertible, registration frameworks^27,53,54^ consider the deformation as the solution of an initial value problem of the form

2 $$\frac{{\rm{d}}{\boldsymbol{\Phi }}(s;{\bf{x}})}{{\rm{d}}s}={\bf{v}}({\boldsymbol{\Phi }}(s;{\bf{x}}),s)\,\,\,\,\mathrm{with}\,\,\,\,{\boldsymbol{\Phi }}(0;{\bf{x}})={\bf{x}}.$$

The mapping **x** → **Φ**(*s*; **x**) defines a family of diffeomorphisms for all time *s* ∈ [0, *τ*]. Hence, it is guaranteed that an inverse of the mapping exists, which can be computed through backward integration. As proposed in DARTEL (diffeomorphic anatomical registration through exponentiated lie algebra), a stationary velocity field framework instead of the large deformation diffeomorphic metric mapping (LDDMM) model^55,56^ allows the velocity field **v** to be constant over time. Using this simplification, the regularity of the deformation field—that is, smoothness and invertibility—is automatically reinforced via forward integration (also called shooting) of this constant velocity field. In this way, a smooth and invertible deformation field can be found by iteratively optimizing the velocity field **v** with respect to *L* using a gradient descent approach.

SyN registration^57^ additionally enforces symmetry between the forward (image to template) **Φ** and backward (template to image) deformation field **Φ**^−1^. SyN considers the full forward and backward deformations to be compositions of half deformations ${{\boldsymbol{\Phi }}}^{\frac{1}{2}}$ and ${{\boldsymbol{\Phi }}}^{-\frac{1}{2}}$ via

$${\boldsymbol{\Phi }}={{\boldsymbol{\Phi }}}^{\frac{1}{2}}\cdot -{{\boldsymbol{\Phi }}}^{-\frac{1}{2}}\,\,\,\,{\mathrm{and}}\,\,\,\,{{\boldsymbol{\Phi }}}^{-1}={{\boldsymbol{\Phi }}}^{-\frac{1}{2}}\cdot -{{\boldsymbol{\Phi }}}^{\frac{1}{2}}.$$

Based on this consideration, SyN adds the dissimilarity between the image and the backward deformed template *D*(**I**, **J** ⋅ **Φ**^−1^) and the dissimilarity between the half forward deformed image and the half backward deformed template $D({\bf{I}}\cdot {{\boldsymbol{\Phi }}}^{\frac{1}{2}},{\bf{J}}\cdot {{\boldsymbol{\Phi }}}^{-\frac{1}{2}})$ to arrive at the loss function

3 $$L({\bf{I}},{\bf{J}},\varPhi )=D({\bf{I}}\cdot \varPhi ,{\bf{J}})+D({\bf{I}},{\bf{J}}\cdot {\varPhi }^{-1})+D\left({\bf{I}}\cdot {\varPhi }^{\frac{1}{2}},{\bf{J}}\cdot {\varPhi }^{\frac{-1}{2}}\right)+\Lambda R(\varPhi ).$$

Using the diffeomorphic mapping in equation (2), velocity fields ${{\bf{v}}}^{\frac{1}{2}}$ and ${{\bf{v}}}^{-\frac{1}{2}}$ are used to generate the half deformations ${{\boldsymbol{\Phi }}}^{\frac{1}{2}}$ and ${{\boldsymbol{\Phi }}}^{-\frac{1}{2}}$.

#### Model architecture and training

The neural network-based image registration framework used for deepmriprep is based on SYMNet^22^ and uses a UNet to predict the forward and backward velocity field ${{\bf{v}}}^{\frac{1}{2}}$ and ${{\bf{v}}}^{-\frac{1}{2}}$. Analogous to the SyN registration, these velocity fields are integrated according to equation (2) to arrive at the half deformation fields ${{\boldsymbol{\Phi }}}^{\frac{1}{2}}$ and ${{\boldsymbol{\Phi }}}^{-\frac{1}{2}}$ via the scaling and squaring method^27,53^ with *τ* = 7 time steps (Supplementary Fig. 30).

Similar to the neural network architecture used for tissue segmentation (see ‘Tissue segmentation’ section in the Methods), the UNet uses instance normalization^49^, a depth of 4, and is doubling the number of channels with increasing depth, starting with 8 channels. However, we apply two modifications: (1) usage of LeakyReLU^58^ instead of ReLU activation layers, and (2) hyperbolic tangent (tanh) activation function in the final layer, ensuring that the UNet’s output conforms to the value range of −1 to 1 used for image coordinates by PyTorch^59^. The model is trained for 50 epochs using the one-cycle learning rate schedule with a maximal learning rate of 0.001.

During initial tests, training with the standard SyN loss function (equation (3)) led to major artifacts in the predicted velocity and deformation field (Supplementary Fig. 31b). To avoid these artifacts, we tested supervised approaches (Supplementary Fig. 31c–e) that utilize deformation fields created by CAT12 (Supplementary Fig. 31a). Using an iterative approach, we determined the velocity fields ${{\bf{v}}}_{{\rm{CAT}}}^{\frac{1}{2}}$ and ${{\bf{v}}}_{{\rm{CAT}}}^{-\frac{1}{2}}$ that produce these given deformation fields **Φ**_CAT_ and ${{\boldsymbol{\Phi }}}_{{\rm{CAT}}}^{-1}$ in our PyTorch-based implementation and used these velocity fields as targets. Using the MSE, the resulting loss function *L*_*v*_ measures disagreements between the predicted velocity fields ${{\bf{v}}}^{\frac{1}{2}}$ and ${{\bf{v}}}^{-\frac{1}{2}}$ and the targets via

$${L}_{{\bf{v}}}\left({{\bf{v}}}^{\frac{1}{2}},{{\bf{v}}}^{-\frac{1}{2}}\right)=\frac{1}{| \varOmega | }\mathop{\sum }\limits_{{\bf{p}}\in \varOmega }| | {{\bf{v}}}_{\mathrm{CAT}}^{\frac{1}{2}}({\bf{p}})-{{\bf{v}}}^{\frac{1}{2}}({\bf{p}})| {| }^{2}+| | {{\bf{v}}}_{\mathrm{CAT}}^{-\frac{1}{2}}({\bf{p}})-{{\bf{v}}}^{-\frac{1}{2}}({\bf{p}})| {| }^{2}.$$

Using this loss function, the predicted velocity fields show fewer artifacts, but based on the resulting Jacobi determinant field **J**_**Φ**_, some inaccuracies remain (Supplementary Fig. 31c). The Jacobi determinant indicates the volume change caused by the deformation for each voxel. By explicitly adding the MSE between the predicted and ground-truth Jacobi determinant **J**_**Φ**_, the loss function

$$\begin{array}{l}{L}_{{\bf{{v}}},{{\bf{{J}}}}_{\Phi }}\left({{\bf{v}}}^{\frac{1}{2}},{{\bf{v}}}^{-\frac{1}{2}}\right)=\frac{1}{|\Omega |}\mathop{\sum }\limits_{{\bf{p}}\in \varOmega }||{{\bf{v}}}_{\mathrm{CAT}}^{\frac{1}{2}}({\bf{p}})-{{\bf{v}}}^{\frac{1}{2}}({\bf{p}})|{|}^{2}\\ +||{{\bf{v}}}_{\mathrm{CAT}}^{-\frac{1}{2}}({\bf{p}})-{{\bf{v}}}^{-\frac{1}{2}}({\bf{p}})|{|}^{2}+||{{{\bf{J}}}_{{\boldsymbol{\Phi }}}}_{\mathrm{CAT}}({\bf{p}})-{{\bf{J}}}_{\Phi }({\bf{p}})|{|}^{2}\end{array}$$

improves regularity of the predicted velocity fields and the resulting Jacobi determinant field (Supplementary Fig. 31d). Finally, we reintroduce the original loss function *L*_SyN_ as

$${L}_{\mathrm{supervised}}\left({{\bf{v}}}^{\frac{1}{2}},{{\bf{v}}}^{-\frac{1}{2}}\right)={L}_{{\bf{v}},{{\bf{J}}}_{\Phi }}\left({{\bf{v}}}^{\frac{1}{2}},{{\bf{v}}}^{-\frac{1}{2}}\right)+\beta {L}_{\mathrm{SyN}}$$

with *β* set to 2 × 10^−5^. The fields predicted with this approach, called supervised SYMNet or sSYMNet, do not show any apparent artifacts (Supplementary Fig. 31e). The implementation of the model and the training procedure is publicly accessible via GitHub at https://github.com/wwu-mmll/deepmriprep-train (ref. ^42^).

### Cross-dataset validation and evaluation metrics

We follow best practices by applying a five-fold cross-dataset validation—that is, cross-validation with datasets grouped together—using the 137 datasets from OpenNeuro-HD (see ‘Training and validation datasets’ section in the Methods). Thereby, we enforce realistic performance measures because all reported results are achieved in datasets unseen during training of the respective model. We apply the same folds across tissue segmentation, image registration and GM masking (see ‘Gray matter masking’ in the Supplementary Information) to avoid data leakage between these processing steps. The test datasets (see ‘Test datasets’ section in the Methods) and the datasets in OpenNeuro-Total and OpenNeuro-Kids, which are not part of OpenNeuro-HD (see ‘OpenNeuro-Total’ section in the ‘Results’), are evaluated with an additional model trained with the full OpenNeuro-HD dataset.

Given that the distribution of performance metrics across images is often skewed, the median is used as a measure of central tendency, complemented by a visual inspection of negative outliers.

To evaluate tissue segmentation and GM masking performance, we use the Dice score DSC, the probabilistic Dice score pDSC and the Jaccard score JSC. The image registrations are evaluated based on the regularity of the deformation field and the dissimilarity between the warped input and the template image. This dissimilarity is measured using the voxel-wise MSE between the images. The deformation field’s regularity—that is, its physical legitimacy—is quantified via the linear elasticity LE (equation (1)).

### Prediction pipeline

The complete deepmriprep pipeline used before the GLM analysis in the ‘VBM analyses’ section in the ‘Results’ consists of six steps: brain extraction, affine registration, tissue segmentation, tissue separation, nonlinear registration and smoothing. After brain extraction using deepbet^14^ with default settings, affine registration is applied using the sum of the MSE (between image and template) and Dice loss (between image brain mask and template brain mask). The affine registration is implemented in torchreg^60^ with zero padding—sensible after brain extraction—and the default two-stage setting with 500 iterations in 12-mm^3^ and successive 100 iterations in 6-mm^3^ image resolution. After tissue segmentation (see ‘Tissue segmentation’ section in the Methods) and before image registration (see ‘Image registration’ section in the Methods), we apply GM masking in the ventricles and around the brain stem to conform the probability masks to an undocumented step in the existing CAT12 preprocessing (see ‘Gray matter masking’ section in the Supplementary Information). After image registration of the GM and WM probability masks, Gaussian smoothing with a 6 mm full width at half maximum kernel is applied. In line with all previous steps, smoothing (a simple convolution operation) is implemented in PyTorch, enabling graphics processing unit acceleration throughout the entire prediction pipeline.

### VBM analyses

To investigate the effect of different preprocessings on the VBM analyses, deepmriprep- and CAT12-preprocessed data are used to examine statistical associations with both biological variables (age, sex and BMI) and psychometric variables (years of education, MDD versus HC, and IQ). To ensure the reliability of results, each VBM analysis is repeated 100 times with a randomly selected 80% subset of the data. Finally, the median *t*-map across these 100 VBM analyses is used to compare the VBM results of deepmriprep and CAT12.

### Statistics and reproducibility

We follow best practices by applying a five-fold cross-dataset validation—that is, cross-validation with datasets grouped together—during training and extensive testing of the models across multiple test datasets. In addition, the number of datasets was maximized by collecting MRI data from OpenNeuro and performing quality checks, resulting in 225 datasets. Each VBM analysis is repeated 100 times with a randomly selected 80% subset of the data. No statistical method was used to predetermine sample size.

### Reporting summary

Further information on research design is available in the Nature Portfolio Reporting Summary linked to this article.

## Supplementary information

Supplementary Information Supplementary Results, Methods, Tables 1–10 and Figs. 1–41.

Reporting Summary

Supplementary Data 1 Zipped NifTI files containing VBM results (*t*-scores) with respect to GM (s6mwp1…) and WM (s6mwp2…) and .csv tables with filenames and Dice scores of tissue segmentations of deepmriprep (dmp) and CAT12 (cat) in the datasets OpenNeuro-Kids, Synthetic Atrophy and Synthetic Artifacts.

## Source data

Source Data Fig. 1 OpenNeuro ID, filepath, age, sex, scanner data, CAT12 quality metrics and Dice scores of deepmriprep and CAT12 tissue segmentations in the OpenNeuro-HD dataset.

Source Data Fig. 2 OpenNeuro ID, filepath, age, sex, scanner data, CAT12 quality metrics and Dice scores between deepmriprep and CAT12 tissue segmentations in the OpenNeuro-Total dataset.

## Data Availability

All raw data of the datasets OpenNeuro-HD, OpenNeuro-Total and OpenNeuro-Kids are publicly available at https://openneuro.org (the respective OpenNeuro dataset IDs and filepaths can be found in Supplementary Data 1). All raw data of the Synthetic Atrophy dataset are publicly available at https://data.csiro.au/collection/csiro:53241. With regard to the Marburg-Münster Affective Disorders Cohort Study (FOR2107/MACS), Münster Neuroimaging Cohort (MNC) and BiDirect dataset, individual raw data are not published due to current EU data protection regulations and the sensitive nature of clinical MRI data but can be made available in form of summary statistics or anonymized aggregation of voxel-wise data upon reasonable request to the corresponding author, within 4 weeks, depending on the required data or results derivates. The data availability of the FOR2107/MACS and MNC dataset is governed by U.D. and the availibility of the BiDirect dataset is governed by K.B. Source data are provided in this paper.

## Code availability

This Brief Communication describes deepmriprep version 0.3.1, which is publicly available via GitHub at https://github.com/wwu-mmll/deepmriprep (ref. ^30^). The implementation of the models and the respective training procedures are publicly available via GitHub at https://github.com/wwu-mmll/deepmriprep-train (ref. ^42^).

## References

[1] Ashburner, J. & Friston, K. J. Voxel-based morphometry—the methods. *NeuroImage* **11**, 805–821 (2000). 10860804 10.1006/nimg.2000.0582

[2] Friston, K. J. et al. Statistical parametric maps in functional imaging: a general linear approach. *Hum. Brain Mapping* **2**, 189–210 (1994).

[3] Ashburner, J. & Friston, K. J. Unified segmentation. *NeuroImage* **26**, 839–851 (2005). 15955494 10.1016/j.neuroimage.2005.02.018

[4] Avants, B. B. et al. A reproducible evaluation of ANTs similarity metric performance in brain image registration. *NeuroImage* **54**, 2033–2044 (2011). 20851191 10.1016/j.neuroimage.2010.09.025 PMC3065962

[5] Cox, R. W. AFNI: software for analysis and visualization of functional magnetic resonance neuroimages. *Comput. Biomed. Res.* **29**, 162–173 (1996). 8812068 10.1006/cbmr.1996.0014

[6] Fischl, B. FreeSurfer. *NeuroImage* **62**, 774–781 (2012). 22248573 10.1016/j.neuroimage.2012.01.021 PMC3685476

[7] Gaser, C. et al. CAT: a computational anatomy toolbox for the analysis of structural MRI data. *GigaScience* **13**, giae049 (2024). 39102518 10.1093/gigascience/giae049 PMC11299546

[8] Smith, S. M. et al. Advances in functional and structural MR image analysis and implementation as FSL. *NeuroImage* **23**, S208–S219 (2004). 15501092 10.1016/j.neuroimage.2004.07.051

[9] Winter, N. R. et al. Quantifying deviations of brain structure and function in major depressive disorder across neuroimaging modalities. *JAMA Psychiatry* **79**, 879–888 (2022). 35895072 10.1001/jamapsychiatry.2022.1780 PMC9330277

[10] Marek, S. et al. Reproducible brain-wide association studies require thousands of individuals. *Nature* **603**, 654–660 (2022). 35296861 10.1038/s41586-022-04492-9 PMC8991999

[11] Bycroft, C. et al. The UK Biobank resource with deep phenotyping and genomic data. *Nature* **562**, 203–209 (2018). 30305743 10.1038/s41586-018-0579-z PMC6786975

[12] Shen, D., Wu, G. & Suk, H. eung-I. l Deep learning in medical image analysis. *Annu. Rev. Biomed. Eng.* **19**, 221–248 (2017). 28301734 10.1146/annurev-bioeng-071516-044442 PMC5479722

[13] Ronneberger, O., Fischer, P. & Brox, T. U-Net: convolutional networks for biomedical image segmentation. In *Medical Image**Computing and Computer-Assisted Intervention* – *MICCAI 2015* (eds Navab, N. et al.) Vol. 9351, 234–241 (Springer, 2015).

[14] Fisch, L. et al. deepbet: fast brain extraction of T1-weighted MRI using convolutional neural networks. *Comput. Biol. Med.* **179**, 108845 (2024). 39002314 10.1016/j.compbiomed.2024.108845

[15] Hoopes, A., Mora, J. S., Dalca, A. V., Fischl, B. & Hoffmann, M. SynthStrip: skull-stripping for any brain image. *NeuroImage* **260**, 119474 (2022). 35842095 10.1016/j.neuroimage.2022.119474 PMC9465771

[16] Isensee, F. et al. Automated brain extraction of multisequence MRI using artificial neural networks. *Hum. Brain Mapping* **40**, 4952–4964 (2019). 10.1002/hbm.24750 PMC6865732 31403237

[17] Kumar, P., Nagar, P., Arora, C. & Gupta, A. U-Segnet: fully convolutional neural network based automated brain tissue segmentation tool. In *2018 25th IEEE**International Conference on Image Processing* 3503–3507 (IEEE, 2018).

[18] Moeskops, P. et al. Automatic segmentation of MR brain images of preterm infants using supervised classification. *NeuroImage* **118**, 628–641 (2015). 26057591 10.1016/j.neuroimage.2015.06.007

[19] Balakrishnan, G., Zhao, A., Sabuncu, M. R., Guttag, J. & Dalca, A. V. VoxelMorph: a learning framework for deformable medical image registration. *IEEE Trans. Med. Imaging* **38**, 1788–1800 (2019). 10.1109/TMI.2019.2897538 30716034

[20] Hoffmann, M., Hoopes, A., Fischl, B. & Dalca, A. V. Anatomy-specific acquisition-agnostic affine registration learned from fictitious images. In *Medical Imaging**2023: Image Processing* Vol. 12464 (eds Oliver, C. et al) 1246402 (SPIE, 2023).

[21] Iglesias, J. E. A ready-to-use machine learning tool for symmetric multi-modality registration of brain MRI. *Sci. Rep.* **13**, 6657 (2023). 37095168 10.1038/s41598-023-33781-0 PMC10126156

[22] Mok, T. C. W. & Chung, A. C. S. Fast symmetric diffeomorphic image registration with convolutional neural networks. In *Proc. IEEE/CVF Conference on Computer Vision and Pattern Recognition* 4644–4653 (IEEE, 2020).

[23] Isensee, F., Jaeger, P. F., Kohl, S. A. A., Petersen, J. & Maier-Hein, K. H. nnU-Net: a self-configuring method for deep learning-based biomedical image segmentation. *Nat. Methods* **18**, 203–211 (2021). 33288961 10.1038/s41592-020-01008-z

[24] Hoffmann, M. et al. SynthMorph: learning contrast-invariant registration without acquired images. *IEEE Trans. Med. Imaging* **41**, 543–558 (2022). 34587005 10.1109/TMI.2021.3116879 PMC8891043

[25] Mårtensson, G. et al. The reliability of a deep learning model in clinical out-of-distribution MRI data: a multicohort study. *Med. Image Anal.* **66**, 101714 (2020). 33007638 10.1016/j.media.2020.101714

[26] Hoffmann, M., Billot, B., Iglesias, J. E., Fischl, B. & Dalca, A. V. Learning MRI contrast-agnostic registration. In *2021 IEEE**18th International Symposium on Biomedical Imaging* 899–903 (IEEE, 2021). 10.1109/isbi48211.2021.9434113 PMC10782386 38213549

[27] Ashburner, J. A fast diffeomorphic image registration algorithm. *NeuroImage* **38**, 95–113 (2007). 17761438 10.1016/j.neuroimage.2007.07.007

[28] Shattuck, D. W. et al. Construction of a 3D probabilistic atlas of human cortical structures. *NeuroImage* **39**, 1064–80 (2008). 18037310 10.1016/j.neuroimage.2007.09.031 PMC2757616

[29] Zhou, X. et al. Choice of voxel-based morphometry processing pipeline drives variability in the location of neuroanatomical brain markers. *Commun. Biol.* **5**, 913 (2022). 36068295 10.1038/s42003-022-03880-1 PMC9448776

[30] Fisch, L. deepmriprep: version 0.3.1. *Zenodo*10.5281/zenodo.17748963 (2025).

[31] Eklund, A., Nichols, T. E. & Knutsson, H. Cluster failure: why fMRI inferences for spatial extent have inflated false-positive rates. *Proc. Natl Acad. Sci. USA* **113**, 7900–7905 (2016). 27357684 10.1073/pnas.1602413113 PMC4948312

[32] Markiewicz, C. J. et al. The OpenNeuro resource for sharing of neuroscience data. *Elife* **10**, e71774 (2021). 34658334 10.7554/eLife.71774 PMC8550750

[33] Rusak, F. et al. Quantifiable brain atrophy synthesis for benchmarking of cortical thickness estimation methods. *Med. Image Anal.* **82**, 102576 (2022). 36126404 10.1016/j.media.2022.102576

[34] Jack Jr, C. R. et al. The Alzheimer’s disease neuroimaging initiative (ADNI): MRI methods. *J. Magn. Reson. Imag.* **27**, 685–691 (2008). 10.1002/jmri.21049 PMC2544629 18302232

[35] Kircher, T. et al. Neurobiology of the major psychoses: a translational perspective on brain structure and function-the FOR2107 consortium. *Eur. Arch. Psychiatry Clin. Neurosci.* **269**, 949–962 (2019). 30267149 10.1007/s00406-018-0943-x

[36] Vogelbacher, C. et al. The Marburg-Münster Affective Disorders Cohort Study (MACS): a quality assurance protocol for MR neuroimaging data. *NeuroImage* **172**, 450–460 (2018). 29410079 10.1016/j.neuroimage.2018.01.079

[37] Dannlowski, U. et al. Disadvantage of social sensitivity: interaction of oxytocin receptor genotype and child maltreatment on brain structure. *Biol. Psychiatry* **80**, 398–405 (2016). 26858213 10.1016/j.biopsych.2015.12.010

[38] Opel, N. et al. Mediation of the influence of childhood maltreatment on depression relapse by cortical structure: a 2-year longitudinal observational study. *Lancet Psychiatry* **6**, 318–326 (2019). 30904126 10.1016/S2215-0366(19)30044-6

[39] Teismann, H. et al. Establishing the bidirectional relationship between depression and subclinical arteriosclerosis - rationale, design, and characteristics of the BiDirect Study. *BMC Psychiatry* **14**, 174 (2014). 24924233 10.1186/1471-244X-14-174 PMC4065391

[40] Teuber, A. et al. MR imaging of the brain in large cohort studies: feasibility report of the population- and patient-based BiDirect study. *Eur. Radiol.* **27**, 231–238 (2017). 27059857 10.1007/s00330-016-4303-9

[41] Tustison, N. J. et al. N4ITK: improved N3 bias correction. *IEEE Trans. Med. Imag.* **29**, 1310–1320 (2010). 10.1109/TMI.2010.2046908 PMC3071855 20378467

[42] Fisch, L. deepmriprep-train: version 0.1.0. *Zenodo*10.5281/zenodo.17749045 (2025).

[43] Fisch, L. niftiai: version 0.3.2. *Zenodo*10.5281/zenodo.17749166 (2025).

[44] Wood, M. L. & Henkelman, R. M. MR image artifacts from periodic motion. *Med. Phys.* **12**, 143–151 (1985). 4000069 10.1118/1.595782

[45] Graves, M. J. & Mitchell, D. G. Body MRI artifacts in clinical practice: a physicist’s and radiologist’s perspective. *J. Magn. Reson. Imag.* **38**, 269–287 (2013). 10.1002/jmri.24288 23960007

[46] Veraart, J., Fieremans, E., Jelescu, I. O., Knoll, F. & Novikov, D. S. Gibbs ringing in diffusion MRI. *Magn. Reson. Med.* **76**, 301–314 (2016). 26257388 10.1002/mrm.25866 PMC4915073

[47] Aja-Fernández, S., Vegas-Sánchez-Ferrero, G. & Tristán-Vega, A. Noise estimation in parallel MRI: GRAPPA and SENSE. *Magn. Reson. Imag.* **32**, 281–290 (2014). 10.1016/j.mri.2013.12.001 24418329

[48] Gudbjartsson, H.áK. on & Patz, S. The Rician distribution of noisy MRI data. *Magn. Reson. Med.* **34**, 910–914 (1995). 8598820 10.1002/mrm.1910340618 PMC2254141

[49] Ulyanov, D., Vedaldi, A. & Lempitsky, V. Instance normalization: the missing ingredient for fast stylization. Preprint at https://arxiv.org/abs/1607.08022 (2017).

[50] Hu, X. et al. Hierarchical multi-class segmentation of glioma images using networks with multi-level activation function. In *Brainlesion: Glioma**, Multiple Sclerosis, Stroke and Traumatic Brain Injuries, Lecture Notes in Computer Science* (eds Crimi, A. et al.) 116–127 (Springer, 2019).

[51] Smith, L. N. Cyclical learning rates for training neural networks. In *2017 IEEE**Winter Conference on Applications of Computer Vision* 464–472 (IEEE, 2017).

[52] Howard, J. & Gugger, S. Fastai: a layered API for deep learning. *Information* **11**, 108 (2020).

[53] Arsigny, V., Pennec, X. & Ayache, N. Polyrigid and polyaffine transformations: a novel geometrical tool to deal with non-rigid deformations—application to the registration of histological slices. *Med. Image Anal.* **9**, 507–523 (2005). 15948656 10.1016/j.media.2005.04.001

[54] Camion, V. & Younes, L. Geodesic interpolating splines. In *Energy Minimization**Methods in Computer Vision and Pattern Recognition, Lecture Notes in Computer Science* (Figueiredo, M. et al.) 513–527 (Springer, 2001).

[55] Beg, M. F., Miller, M. I., Trouvé, A. & Younes, L. Computing large deformation metric mappings via geodesic flows of diffeomorphisms. *Int. J. Comput. Vis.* **61**, 139–157 (2005).

[56] Vialard, F. rançois-X. avier, Risser, L., Rueckert, D. & Cotter, C. J. Diffeomorphic 3D image registration via geodesic shooting using an efficient adjoint calculation. *Int. J. Comput. Vis.* **97**, 229–241 (2012).

[57] Avants, B. B., Epstein, C. L., Grossman, M. & Gee, J. C. Symmetric diffeomorphic image registration with cross-correlation: evaluating automated labeling of elderly and neurodegenerative brain. *Med. Image Anal.* **12**, 26–41 (2008). 17659998 10.1016/j.media.2007.06.004 PMC2276735

[58] Xu, B., Wang, N., Chen, T. & Li, M. Empirical evaluation of rectified activations in convolutional network. Preprint at https://arxiv.org/abs/1505.00853 (2015).

[59] Paszke, A. et al. PyTorch: an imperative style, high-performance deep learning library. In *Proc. 33rd International Conference on Neural Information Processing Systems* 8026–8037 (Curran, 2019).

[60] Fisch, L. torchreg—Lightweight image registration library using PyTorch. *GitHub* https://github.com/codingfisch/torchreg (2023).

