# Supplementary material for: deepmriprep: voxel-based morphometry preprocessing via deep neural networks
Source: Nat Comput Sci. 2026 Jan 30;6(3):250–9. doi: 10.1038/s43588-026-00953-7 (PMC13021509; doi:10.1038/s43588-026-00953-7)
Supplement: Supplementary file 1 — Supplementary Results, Methods, Tables 1–10 and Figs. 1–41. [file 43588_2026_953_MOESM1_ESM.pdf]

nature computational science

Supplementary information https://doi.org/10.1038/s43588-026-00953-7

deepmriprep: voxel-based morphometry preprocessing via deep neural networks

In the format provided by the authors and unedited

Supplementary Information

Supplementary Table and Figure Legends

Supplementary Table 1: Median **Dice scores** of deepmriprep and CAT12 for CSF, GM, WM, and foreground across OpenNeuro-HD (top left), OpenNeuro-Total (top right), Synthetic Atrophy (bottom left), and OpenNeuro-Kids (bottom right).

Supplementary Table 2: Median **probabilistic Dice scores** of deepmriprep and CAT12 for CSF, GM, WM, and foreground across the same four datasets.

Supplementary Table 3: Median **Jaccard scores** of deepmriprep and CAT12 for CSF, GM, WM, and foreground across the same four datasets.

Supplementary Table 4: Dice scores of deepmriprep and CAT12 on 20 Synthetic Atrophy images with medium and strong variants of seven synthetic artifacts.

Supplementary Table 5: Probabilistic Dice scores of deepmriprep and CAT12 under the same synthetic artifact conditions.

Supplementary Table 6: Jaccard scores of deepmriprep and CAT12 under the same synthetic artifact conditions.

Supplementary Table 7: Correlation coefficients between deepmriprep- and CAT12-based unthresholded t-score maps (GM and WM) across pooled and three individual cohorts.

Supplementary Table 8: Maximum absolute t-scores from **gray-matter** GLM analyses versus age, sex, BMI, education, HC vs. MDD, and IQ.

Supplementary Table 9: Maximum absolute t-scores from **white-matter** GLM analyses versus the same variables.

Supplementary Table 10: MRI scanner models and subject counts in OpenNeuro-Total, OpenNeuro-HD, and OpenNeuro-Kids.

Supplementary Figure 1: Dice, probabilistic Dice, and Jaccard scores of deepmriprep across validation folds.

Supplementary Figure 2: Probabilistic Dice scores of deepmriprep and CAT12 with worst-case segmentation examples.

Supplementary Figure 3: Jaccard scores of deepmriprep and CAT12 with worst-case segmentation examples.

Supplementary Figure 4: Dice scores of tissue segmentations of deepmriprep and CAT12 in OpenNeuro-Total with worst-case segmentation examples.

Supplementary Figure 5: 16 OpenNeuro-Total images showing largest disagreement between deepmriprep and CAT12.

Supplementary Figure 6: Registration metrics and template warping examples on OpenNeuro-HD.

Supplementary Figure 7: Registration metrics of deepmriprep across validation folds.

Supplementary Figure 8: Gray-matter t-score maps of the **pooled data**.
Supplementary Figure 9: Gray-matter t-score maps of the **FOR2107/MACS cohort**.
Supplementary Figure 10: Gray-matter t-score maps of the **MNC cohort**.
Supplementary Figure 11: Gray-matter t-score maps of the **BiDirect cohort**.
Supplementary Figure 12: White-matter t-score maps of the **pooled data**.
Supplementary Figure 13: White-matter t-score maps of the **FOR2107/MACS cohort**.
Supplementary Figure 14: White-matter t-score maps of the **MNC cohort**.
Supplementary Figure 15: White-matter t-score maps **BiDirect cohort**.
Supplementary Figure 16: Bland-Altman plots of GM/WM volume differences in five LPBA40 ROIs with **lowest correlation**.
Supplementary Figure 17: Bland-Altman plots of GM/WM volume differences in five LPBA40 ROIs with **second-lowest correlation**.
Supplementary Figure 18: Bland-Altman plots of GM/WM volume differences in five LPBA40 ROIs with **third-lowest correlation**.
Supplementary Figure 19: Bland-Altman plots of GM/WM volume differences in five LPBA40 ROIs with **fourth-lowest correlation**.
Supplementary Figure 20: Bland-Altman plots of GM/WM volume differences in five LPBA40 ROIs with **fifth-lowest correlation**.
Supplementary Figure 21: Bland-Altman plots of GM/WM volume differences in five LPBA40 ROIs with **highest correlation**.
Supplementary Figure 22: Processing time comparison on high-end and low-end hardware.
Supplementary Figure 23: Examples of image dropouts due to orientation, masking, and motion artifacts.
Supplementary Figure 24: Demographic and acquisition parameter distributions in OpenNeuro datasets.
Supplementary Figure 25: Example images with medium and strong synthetic artifacts.
Supplementary Figure 26: Age and sex distributions in clinical cohorts.
Supplementary Figure 27: Data augmentation examples.
Supplementary Figure 28: Optimized patch positions on CAT12 template.
Supplementary Figure 29: Step activation function and tissue value distribution.
Supplementary Figure 30: Diffeomorphic registration pipeline.
Supplementary Figure 31: Ablation study of registration loss functions.
Supplementary Figure 32: Dice scores and worst-case examples on OpenNeuro-Kids.
Supplementary Figure 33: 16 OpenNeuro-Kids images with largest disagreement.
Supplementary Figure 34: Segmentation accuracy and volume differences on Synthetic Atrophy dataset.
Supplementary Figure 35: Probabilistic Dice and Jaccard on Synthetic Atrophy dataset.
Supplementary Figure 36: **Dice scores** under synthetic artifacts.
Supplementary Figure 37: **Probabilistic Dice scores** under synthetic artifacts.
Supplementary Figure 38: **Jaccard scores** under synthetic artifacts.
Supplementary Figure 39: Gray-matter masking region highlighted.

Supplementary Figure 40: Patches used for gray-matter masking.

Supplementary Figure 41: Gray-matter masking performance and worst-case example.

Supplementary Results

OpenNeuro-Kids

Despite only being trained with MRIs from healthy adults, the results show high agreement between deepmriprep and CAT12 for most of the respective tissue maps resulting in a median Dice score DSC_{median} of 90.2 (see Supplementary Figure 32 and Supplementary Table 1), a median probabilistic Dice score of 80.2 and a median Jaccard score of 83.4 (see Supplementary Table 2 and 3). With a minimal Dice score DSC_{median} of 80.6, there are no major errors like the unusable tissue maps that CAT12 produced for some OpenNeuro-Total MRIs. In the five tissue maps with the 0.0^{th}-0.4^{th} percentile Dice score, CAT12 shows a tendency to overestimate the amount of gray matter often omitting small areas of CSF (for instance, the sulci) and WM (for instance in the cerebellum). Furthermore, in the 16 tissue maps with the lowest Dice score, ringing artifacts close to the skull often result in erroneous strands of WM or GM in the respective CAT12 tissue maps (see Supplementary Figure 33). These ringing artifacts are less pronounced in the tissue maps produced by deepmriprep.

Synthetic Atrophy

In the Synthetic Atrophy dataset, deepmriprep matches the ground truth tissue maps with higher median foreground metrics ($DSC_{\text{median}} = 85.0$, $pDSC_{\text{median}} = 82.3$, $JSC_{\text{median}} = 74.8$) than CAT12 ($DSC_{\text{median}} = 82.4$, $pDSC_{\text{median}} = 80.2$, $JSC_{\text{median}} = 71.6$). Especially with respect to CSF, deepmriprep consistently shows higher median segmentation metrics than CAT12, while CAT12 and deepmriprep show similar median metrics for GM and WM (see Supplementary Figure 34 and 35 and Supplementary Table 1, 2, and 3).

Comparison of measured with ground truth tissue volumes confirms that especially in CSF deepmriprep matches ground truth better than CAT12. Although both tools measure less CSF volume than the ground truth, deepmriprep results in an MAE of 273 mL while CAT12 results in an MAE of 333 mL. GM and WM measured with deepmriprep resulted in a lower 24 mL MAE compared to 39 mL for CAT12 and a higher R^2 of 0.86 compared to 0.68 with CAT12 overestimating GM and underestimating WM volumes.

Finally, the volume differences introduced by synthetic atrophy (see Section 'Test Datasets' in the Methods) reveal a tendency of CAT12 and deepmriprep to underestimate atrophy. For CSF and WM the metrics of deepmriprep ($\text{MAE}^{\text{CSF}} = 33$ mL, $\text{MAE}^{\text{WM}} = 3$ mL, $R^{2\,\text{CSF}} = 0.03$) again indicate a better performance than CAT12 ($\text{MAE}^{\text{CSF}} = 38$ mL, $\text{MAE}^{\text{WM}} = 6$ mL, $R^{2\,\text{CSF}} = 0.08$), while the GM differences result in a lower MAE of 9 mL for CAT12 and a higher R^2 of 0.96 compared to deepmriprep's MAE of 12 mL and R^2 of 0.94.

Synthetic Scanner Artifacts

Across all seven synthetic scanner artifacts, deepmriprep's tissue segmentation maps resulted in higher median foreground metrics than CAT12 (see Supplementary Figure 36, 37, and 38 and Supplementary Table 4, 5, and 6). Especially for MRIs with stronger artifacts, deepmriprep maintains higher foreground metrics.

Consistent with previous results, the Dice score of CSF shows the largest variability across MRIs, with deepmriprep consistently achieving higher median metrics. The GM and WM metrics show less variability and do not consistently favor one tool over the other across all scanner artifacts. With respect to GM and WM, the metrics indicate that deepmriprep retains higher performance for motion, ringing and spike artifacts while CAT12 retains higher performance for Rician noise.

Supplementary Methods

Gray Matter Masking

Based on SPM, CAT12 outputs tissue segmentation maps (file-prefix "p0") with continuous values ranging from 0 to 3. The values 0, 1, 2 and 3 encode the segmentation classes background, cerebrospinal fluid (CSF), gray matter (GM), and white matter (WM). Intermediate values, like for instance 2.4, can thereby easily be mapped to the respective voxel containing 40% gray matter and 60% white matter. Applying this mapping to all voxels results in the tissue probability maps for GM, WM, and CSF (file-prefixes "p1", "p2", and "p3"). By comparing these probability maps with the respective segmentation map, we found voxels positioned on the edge of the ventricles and the brain stem that did not follow this mapping (see Supplementary Figure 39). The GM probability in these voxels is set to zero, and the WM and CSF probabilities are each increased by half of the original GM probability to ensure a tissue probability sum of 100%.

To make deepmriprep conformant with this GM masking, we employed the UNet architecture already used for tissue segmentation (see Section 'Tissue Segmentation' in the Methods) to predict the corresponding voxels that would be masked in CAT12. As model input, a 224x288x256 voxel region of the tissue segmentation map with a resolution of 0.5 mm is used. We follow a patchwise approach similar to the tissue segmentation model with two instead of 27 static patch positions (see Supplementary Figure 40). The two patches each cover 128x288x256 voxels and are placed symmetrically on the left and right hemispheres. We flip all right hemisphere patches along the sagittal axis during model training so that the resulting model can be used to predict the GM mask in both hemispheres (see Supplementary Figure 41).

Supplementary Tables

Supplementary Table 1: Median Dice score (± standard deviation) of deepmriprep and CAT12 with respect to the cerebrospinal fluid (CSF), gray matter (GM), white matter (WM) and foreground (mean of CSF, GM and WM) in the OpenNeuro-HD (top left), OpenNeuro-Total (top right), Synthetic Atrophy (bottom left), and OpenNeuro-Kids dataset (bottom right).

**OpenNeuro-HD**	deepmriprep	CAT12
CSF	91.1 ± 2.9	85.6 ± 4.3
GM	96.4 ± 0.6	96.3 ± 0.5
WM	97.5 ± 0.4	97.4 ± 0.3
Foreground	95.0 ± 1.0	93.1 ± 1.4

**OpenNeuro-Total**	deepmriprep vs. CAT12
CSF	85.9 ± 6.5
GM	96.0 ± 3.7
WM	97.4 ± 3.5
Foreground	93.1 ± 4.1

**Synthetic Atrophy**	deepmriprep	CAT12
CSF	73.7 ± 5.7	66.2 ± 6.1
GM	87.6 ± 1.7	87.7 ± 1.6
WM	93.6 ± 0.5	93.5 ± 0.6
Foreground	85.0 ± 1.8	82.4 ± 1.8

**OpenNeuro-Kids**	deepmriprep vs. CAT12
CSF	77.8 ± 4.7
GM	96.0 ± 0.8
WM	96.9 ± 0.9
Foreground	90.2 ± 1.9

Supplementary Table 2: Median probabilistic Dice score (± standard deviation) of deepmriprep and CAT12 with respect to the cerebrospinal fluid (CSF), gray matter (GM), white matter (WM) and foreground (mean of CSF, GM and WM) in the OpenNeuro-HD (top left), OpenNeuro-Total (top right), Synthetic Atrophy (bottom left), and OpenNeuro-Kids dataset (bottom right).

**OpenNeuro-HD**	deepmriprep	CAT12
CSF	79.2 ± 3.9	71.1 ± 5.2
GM	86.3 ± 1.1	83.9 ± 1.4
WM	90.0 ± 0.6	88.4 ± 0.7
Foreground	85.1 ± 1.2	81.1 ± 1.7

**OpenNeuro-Total**	deepmriprep vs. CAT12
CSF	73.5 ± 5.8
GM	86.0 ± 3.5
WM	89.5 ± 3.2
Foreground	83.0 ± 3.6

**Synthetic Atrophy**	deepmriprep	CAT12
CSF	71.7 ± 5.7	64.6 ± 6.1
GM	83.7 ± 0.6	84.4 ± 1.9
WM	91.6 ± 0.4	92.0 ± 0.6
Foreground	82.3 ± 1.7	80.2 ± 1.8

**OpenNeuro-Kids**	deepmriprep vs. CAT12
CSF	65.6 ± 4.3
GM	88.0 ± 1.0
WM	88.6 ± 1.0
Foreground	80.7 ± 1.6

Supplementary Table 3: Median Jaccard score (± standard deviation) of deepmriprep and CAT12 with respect to the cerebrospinal fluid (CSF), gray matter (GM), white matter (WM) and foreground (mean of CSF, GM and WM) in the OpenNeuro-HD (top left), OpenNeuro-Total (top right), Synthetic Atrophy (bottom left), and OpenNeuro-Kids dataset (bottom right).

**OpenNeuro-HD**	deepmriprep	CAT12
CSF	83.7 ± 4.6	74.9 ± 6.2
GM	93.0 ± 1.1	92.8 ± 1.0
WM	95.0 ± 0.8	94.9 ± 0.6
Foreground	95.0 ± 1.7	87.6 ± 2.1

**OpenNeuro-Total**	deepmriprep vs. CAT12
CSF	75.3 ± 8.0
GM	92.4 ± 4.3
WM	94.9 ± 3.7
Foreground	87.5 ± 4.6

**Synthetic Atrophy**	deepmriprep	CAT12
CSF	58.4 ± 7.1	49.5 ± 6.9
GM	77.9 ± 2.7	78.0 ± 2.4
WM	87.9 ± 0.9	87.9 ± 1.0
Foreground	74.8 ± 2.2	71.6 ± 2.0

**OpenNeuro-Kids**	deepmriprep vs. CAT12
CSF	63.7 ± 6.0
GM	92.2 ± 1.5
WM	93.9 ± 1.7
Foreground	83.3 ± 2.6

Supplementary Table 4: Dice scores of deepmriprep and CAT12 across 20 original images from the Synthetic Atrophy dataset with medium and strong variants of seven synthetic image artifacts with respect to the cerebrospinal fluid (CSF), gray matter (GM), white matter (WM), and foreground (mean of CSF, GM and WM).

**Artifact**	**Tool**	Foreground	CSF	GM	WM
Medium Rician Noise	deepmriprep	86.2 ± 2.1	76.8 ± 6.7	88.9 ± 0.7	92.9 ± 0.6
	CAT12	82.2 ± 2.1	63.9 ± 6.9	89.1 ± 0.7	93.2 ± 0.6
Strong Rician Noise	deepmriprep	86.2 ± 1.7	79.3 ± 5.7	87.2 ± 0.9	91.5 ± 0.8
	CAT12	81.5 ± 2.4	63.6 ± 8.1	88.3 ± 0.9	92.5 ± 0.7
Medium Bias Field	deepmriprep	85.2 ± 2.1	71.9 ± 6.6	89.4 ± 0.6	93.6 ± 0.5
	CAT12	82.2 ± 3.0	63.9 ± 9.2	89.5 ± 1.5	93.6 ± 0.6
Strong Bias Field	deepmriprep	83.6 ± 2.0	69.4 ± 6.4	89.1 ± 1.3	93.2 ± 0.7
	CAT12	80.7 ± 5.8	60.4 ± 8.7	88.4 ± 7.5	92.6 ± 4.5
Medium Blurring	deepmriprep	85.9 ± 2.0	74.3 ± 6.3	89.3 ± 0.6	93.5 ± 0.5
	CAT12	81.7 ± 2.2	62.9 ± 7.5	89.3 ± 0.6	93.5 ± 0.6
Strong Blurring	deepmriprep	84.7 ± 1.5	75.5 ± 5.2	86.4 ± 0.9	92.0 ± 0.7
	CAT12	77.7 ± 2.2	55.0 ± 8.0	85.8 ± 1.1	91.5 ± 0.9
Medium Ghosting	deepmriprep	85.9 ± 1.9	75.2 ± 5.9	88.9 ± 0.6	93.1 ± 0.6
	CAT12	81.3 ± 2.5	61.5 ± 8.3	88.8 ± 0.7	93.0 ± 0.6
Strong Ghosting	deepmriprep	83.2 ± 1.5	75.4 ± 4.9	84.3 ± 0.9	89.7 ± 0.7
	CAT12	76.1 ± 1.9	57.7 ± 7.0	81.9 ± 1.4	87.8 ± 1.2
Medium Motion	deepmriprep	71.9 ± 1.0	63.3 ± 4.2	70.0 ± 1.6	81.8 ± 0.9
	CAT12	66.8 ± 1.8	48.5 ± 6.9	70.2 ± 1.6	81.4 ± 1.0
Strong Motion	deepmriprep	62.8 ± 0.9	54.7 ± 2.7	60.5 ± 2.3	73.7 ± 1.1
	CAT12	57.9 ± 1.1	39.6 ± 5.4	59.3 ± 2.1	73.6 ± 1.4
Medium Ringing	deepmriprep	83.2 ± 2.0	70.8 ± 6.6	86.8 ± 0.8	91.8 ± 0.7
	CAT12	80.3 ± 2.9	63.7 ± 9.1	85.7 ± 1.1	90.8 ± 0.9
Strong Ringing	deepmriprep	80.0 ± 1.9	67.5 ± 6.4	83.4 ± 1.0	89.6 ± 0.8
	CAT12	73.7 ± 2.3	54.7 ± 8.1	78.2 ± 1.5	86.0 ± 1.3
Medium Spike	deepmriprep	84.3 ± 2.1	71.3 ± 6.6	88.8 ± 0.8	93.0 ± 0.8
	CAT12	81.3 ± 3.6	67.0 ± 8.6	87.3 ± 3.0	91.7 ± 2.6
Strong Spike	deepmriprep	83.6 ± 2.5	69.8 ± 6.9	87.4 ± 1.6	92.0 ± 1.2
	CAT12	69.3 ± 11.9	52.2 ± 16.2	69.9 ± 14.8	81.7 ± 7.5

Supplementary Table 5: Probabilistic Dice scores of deepmriprep and CAT12 across 20 original images from the Synthetic Atrophy dataset with medium and strong variants of seven synthetic image artifacts with respect to the cerebrospinal fluid (CSF), gray matter (GM), white matter (WM), and foreground (mean of CSF, GM and WM).

**Artifact**	**Tool**	Foreground	CSF	GM	WM
Medium Rician Noise	deepmriprep	83.4 ± 2.0	74.2 ± 6.6	85.3 ± 0.8	90.7 ± 0.6
	CAT12	79.8 ± 2.0	61.9 ± 6.8	86.0 ± 0.9	91.3 ± 0.7
Strong Rician Noise	deepmriprep	83.2 ± 1.6	76.8 ± 5.6	83.3 ± 1.1	89.2 ± 0.8
	CAT12	79.1 ± 2.3	61.5 ± 8.0	84.9 ± 1.1	90.4 ± 0.8
Medium Bias Field	deepmriprep	82.6 ± 2.0	69.6 ± 6.5	86.0 ± 0.7	91.5 ± 0.5
	CAT12	79.9 ± 2.9	61.8 ± 9.0	86.4 ± 1.5	91.8 ± 0.6
Strong Bias Field	deepmriprep	81.1 ± 2.0	67.1 ± 6.3	85.6 ± 1.4	91.1 ± 0.8
	CAT12	78.5 ± 5.7	58.2 ± 8.6	85.2 ± 7.5	90.8 ± 4.3
Medium Blurring	deepmriprep	83.0 ± 1.9	71.9 ± 6.2	85.7 ± 0.7	91.3 ± 0.6
	CAT12	78.9 ± 2.1	60.4 ± 7.4	85.3 ± 0.8	91.1 ± 0.7
Strong Blurring	deepmriprep	81.8 ± 1.4	73.3 ± 5.1	82.2 ± 1.0	89.6 ± 0.8
	CAT12	74.2 ± 2.2	52.7 ± 7.9	81.0 ± 1.1	88.4 ± 0.9
Medium Ghosting	deepmriprep	83.2 ± 1.8	72.8 ± 5.8	85.4 ± 0.7	91.0 ± 0.6
	CAT12	78.8 ± 2.4	59.4 ± 8.2	85.6 ± 0.8	90.9 ± 0.7
Strong Ghosting	deepmriprep	80.6 ± 1.5	73.3 ± 4.9	80.9 ± 1.0	87.7 ± 0.7
	CAT12	73.7 ± 1.9	55.8 ± 6.9	78.4 ± 1.5	85.7 ± 1.2
Medium Motion	deepmriprep	70.6 ± 1.0	62.3 ± 4.3	68.0 ± 1.6	80.8 ± 0.9
	CAT12	65.8 ± 1.8	47.8 ± 7.0	68.2 ± 1.5	80.4 ± 1.0
Strong Motion	deepmriprep	62.0 ± 0.8	54.3 ± 2.8	59.1 ± 2.1	72.9 ± 1.1
	CAT12	57.0 ± 1.2	39.1 ± 5.6	58.1 ± 1.8	72.7 ± 1.4
Medium Ringing	deepmriprep	80.4 ± 1.9	68.3 ± 6.4	83.0 ± 0.9	89.6 ± 0.7
	CAT12	77.9 ± 2.8	61.7 ± 9.0	82.5 ± 1.1	88.8 ± 0.9
Strong Ringing	deepmriprep	77.3 ± 1.8	65.1 ± 6.3	79.5 ± 1.1	87.5 ± 0.8
	CAT12	72.2 ± 2.3	53.6 ± 8.0	75.9 ± 1.4	84.5 ± 1.3
Medium Spike	deepmriprep	81.5 ± 2.1	68.8 ± 6.5	85.1 ± 1.1	90.7 ± 0.9
	CAT12	78.9 ± 3.4	65.2 ± 8.6	83.8 ± 2.9	89.5 ± 2.6
Strong Spike	deepmriprep	80.4 ± 2.6	67.2 ± 6.8	83.4 ± 1.9	89.6 ± 1.3
	CAT12	67.7 ± 11.0	51.1 ± 15.8	68.9 ± 13.4	81.3 ± 6.6

Supplementary Table 6: Jaccard scores of deepmriprep and CAT12 across 20 original images from the Synthetic Atrophy dataset with medium and strong variants of seven synthetic image artifacts with respect to the cerebrospinal fluid (CSF), gray matter (GM), white matter (WM), and foreground (mean of CSF, GM and WM).

**Artifact**	**Tool**	Foreground	CSF	GM	WM
Medium Rician Noise	deepmriprep	76.4 ± 2.7	62.3 ± 8.7	80.0 ± 1.0	86.7 ± 1.0
	CAT12	71.8 ± 2.2	47.0 ± 7.7	80.3 ± 1.1	87.3 ± 1.1
Strong Rician Noise	deepmriprep	76.1 ± 2.2	65.7 ± 7.7	77.4 ± 1.4	84.3 ± 1.3
	CAT12	70.9 ± 2.6	46.6 ± 9.2	79.0 ± 1.4	86.0 ± 1.2
Medium Bias Field	deepmriprep	75.3 ± 2.4	56.2 ± 8.0	80.9 ± 0.9	87.9 ± 0.9
	CAT12	72.0 ± 3.3	47.0 ± 9.9	81.0 ± 2.3	88.0 ± 1.1
Strong Bias Field	deepmriprep	73.5 ± 2.4	53.1 ± 7.5	80.3 ± 2.0	87.3 ± 1.3
	CAT12	70.4 ± 7.6	43.2 ± 9.9	79.2 ± 10.1	86.3 ± 7.0
Medium Blurring	deepmriprep	76.1 ± 2.4	59.1 ± 8.0	80.7 ± 0.9	87.8 ± 0.9
	CAT12	71.4 ± 2.4	45.8 ± 8.3	80.7 ± 1.0	87.8 ± 1.0
Strong Blurring	deepmriprep	74.0 ± 1.9	60.7 ± 6.7	76.0 ± 1.3	85.2 ± 1.2
	CAT12	66.2 ± 2.1	38.0 ± 8.4	75.1 ± 1.6	84.3 ± 1.5
Medium Ghosting	deepmriprep	75.9 ± 2.3	60.2 ± 7.5	80.0 ± 1.0	87.1 ± 1.0
	CAT12	70.8 ± 2.7	44.4 ± 9.2	79.8 ± 1.1	86.9 ± 1.1
Strong Ghosting	deepmriprep	71.7 ± 1.9	60.6 ± 6.3	72.8 ± 1.3	81.3 ± 1.2
	CAT12	63.2 ± 1.9	40.5 ± 7.2	69.3 ± 2.0	78.2 ± 1.9
Medium Motion	deepmriprep	56.7 ± 1.0	46.3 ± 4.5	53.8 ± 1.9	69.3 ± 1.2
	CAT12	51.9 ± 1.5	32.0 ± 6.3	54.1 ± 1.9	68.6 ± 1.4
Strong Motion	deepmriprep	46.3 ± 0.9	37.7 ± 2.6	43.3 ± 2.3	58.4 ± 1.3
	CAT12	42.2 ± 0.9	24.7 ± 4.5	42.2 ± 2.2	58.2 ± 1.8
Medium Ringing	deepmriprep	72.1 ± 2.3	54.8 ± 7.8	76.7 ± 1.2	84.9 ± 1.1
	CAT12	68.4 ± 3.0	46.7 ± 9.4	75.0 ± 1.6	83.2 ± 1.5
Strong Ringing	deepmriprep	67.6 ± 2.1	50.9 ± 7.3	71.5 ± 1.5	81.2 ± 1.3
	CAT12	60.0 ± 2.3	37.7 ± 8.2	64.2 ± 2.0	75.4 ± 1.9
Medium Spike	deepmriprep	74.0 ± 2.6	55.4 ± 8.0	79.9 ± 1.4	86.9 ± 1.3
	CAT12	70.6 ± 4.5	50.4 ± 9.2	77.5 ± 4.6	84.7 ± 4.3
Strong Spike	deepmriprep	72.6 ± 3.1	53.6 ± 8.1	77.6 ± 2.5	85.1 ± 2.1
	CAT12	54.7 ± 13.5	35.3 ± 15.1	53.7 ± 17.6	69.1 ± 11.0

Supplementary Table 7: Correlation coefficients between deepmriprep- and CAT12-based unthresholded t-score maps resulting from GLM analysis between tissue volume (gray matter and white matter) and age, sex, body mass index (BMI), years of education, HC vs. MDD (healthy control vs. major depressive disorder) and intelligence quotient (IQ) in the Marburg-Münster Affective Disorders Cohort Study (FOR2107/MACS), the Münster Neuroimaging Cohort Study (MNC), the BiDirect study and the pooled data.

**R**	**Gray Matter**				**White Matter**			
**Variable of interest**	Pooled	FOR2107 /MACS	MNC	BiDirect	Pooled	FOR2107 /MACS	MNC	BiDirect
Age	0.96	0.95	0.94	0.93	0.90	0.92	0.80	0.87
Sex	0.89	0.93	0.72	0.85	0.73	0.86	0.71	0.71
BMI	0.75	0.77	0.83	0.73	0.52	0.68	0.75	0.60
Education Years	0.86	0.85	0.82	0.86	0.82	0.83	0.87	0.80
HC vs. MDD	0.89	0.89	0.85	0.85	0.85	0.90	0.81	0.74
IQ	0.84	0.86	0.81	-	0.88	0.87	0.89	-

Supplementary Table 8: Maximum of absolute t-scores of deepmriprep- and CAT12-based GLM analyses between gray matter volume and age, sex, body mass index (BMI), years of education, HC vs. MDD (healthy control vs. major depressive disorder) and intelligence quotient (IQ) in the Marburg-Münster Affective Disorders Cohort Study (FOR2107/MACS), the Münster Neuroimaging Cohort Study (MNC), the BiDirect study and the pooled data.

**Gray Matter** $\|t\|_{max}$	**Pooled**		**FOR2107/MACS**		**MNC**		**BiDirect**	
**Variable of interest**	deep-mriprep	CAT12	deep-mriprep	CAT12	deep-mriprep	CAT12	deep-mriprep	CAT12
Age	39.52	39.01	31.91	30.68	25.24	24.79	13.78	12.80
Sex	20.39	20.07	18.66	17.92	10.18	13.17	9.41	9.04
BMI	7.25	12.80	6.97	9.56	5.07	5.80	6.25	10.86
Education Years	5.39	5.58	4.21	4.51	4.54	5.30	5.76	5.30
HC vs. MDD	6.37	6.30	4.55	4.70	4.51	4.77	4.91	5.03
IQ	4.04	4.22	3.73	4.09	4.13	4.49	-	-

Supplementary Table 9: Maximum of absolute t-scores of deepmriprep- and CAT12-based GLM analyses between white matter volume and age, sex, body mass index (BMI), years of education, HC vs. MDD (healthy control vs. major depressive disorder) and intelligence quotient (IQ) in the Marburg-Münster Affective Disorders Cohort Study (FOR2107/MACS), the Münster Neuroimaging Cohort Study (MNC), the BiDirect study and the pooled data.

**White Matter** $\|t\|_{max}$	**Pooled**		**FOR2107/MACS**		**MNC**		**BiDirect**	
**Variable of interest**	deep-mriprep	CAT12	deep-mriprep	CAT12	deep-mriprep	CAT12	deep-mriprep	CAT12
Age	33.43	34.84	24.39	28.95	20.67	18.88	16.93	14.54
Sex	16.04	18.40	10.60	12.94	10.53	13.99	9.14	8.02
BMI	9.71	12.80	5.82	8.92	5.47	6.16	6.62	10.28
Education Years	5.19	6.76	4.30	4.00	5.94	6.96	4.45	4.50
HC vs. MDD	5.05	4.92	5.01	4.38	4.86	3.69	3.91	4.59
IQ	4.62	4.55	4.21	4.10	4.56	5.55	-	-

Supplementary Table 10: Magnetic Resonance Imaging (MRI) scanner models occurring in the compilations of datasets OpenNeuro-Total, OpenNeuro-HD, and OpenNeuro-Kids.

	**OpenNeuro-Total**		**OpenNeuro-HD**		**OpenNeuro-Kids**	
**MRI scanner model**	No. of datasets	No. of subjects	No. of datasets	No. of subjects	No. of datasets	No. of subjects
General Electric Discovery	15	360	8	40	1	45
General Electric Excite	1	15	0	0	1	47
General Electric Signa	3	77	0	0	1	9
General Electric Unknown	1	38	0	0	2	25
Philips Achieva	18	1840	10	50	1	11
Philips Achieva X	4	149	4	20	0	0
Philips Ingenia	5	237	4	20	0	0
Philips Intera	5	97	1	5	0	0
Siemens Allegra	14	64	0	0	2	22
Siemens Avanto	4	175	4	20	0	0
Siemens Biograph	3	105	1	5	0	0
Siemens Magnetom	5	95	0	0	0	0
Siemens Prisma	43	1531	33	165	1	34
Siemens Skyra	18	944	18	90	3	217
Siemens Trio	55	1964	47	235	6	457
Siemens Unknown	1	49	1	5	0	0
Siemens Verio	8	166	4	20	0	0
Siemens Vida	1	50	1	5	0	0
Unknown	4	122	1	5	0	0

Supplementary Figures

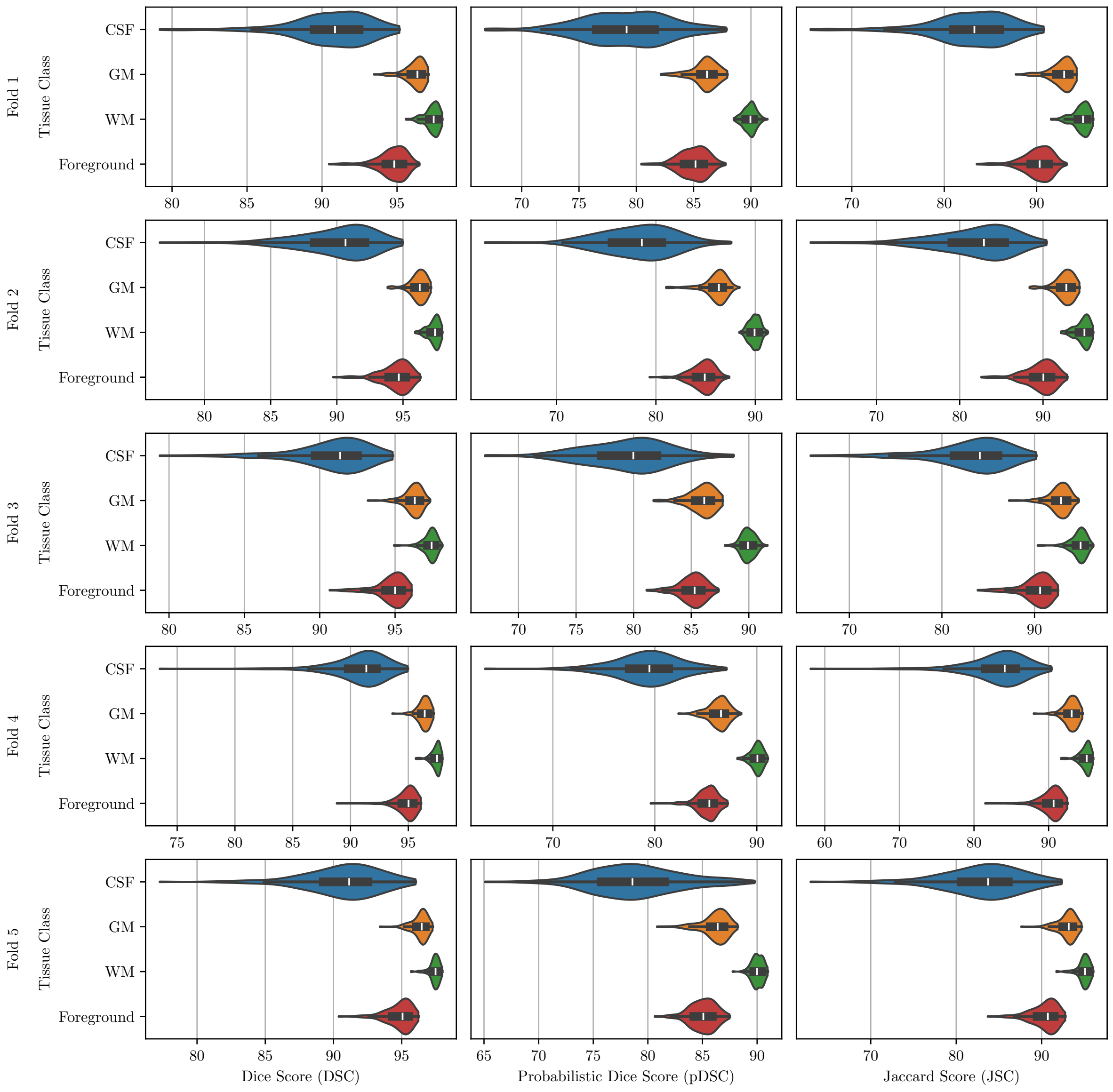

Supplementary Figure 1: Dice score (left), Probabilistic Dice score (center) and Jaccard score (right) of deepmriprep with respect to the cerebrospinal fluid (CSF), gray matter (GM), white matter (WM) and foreground (mean of CSF, GM and WM) across 685 MRIs from OpenNeuro-HD splitted by validation folds. Violin plot density traces terminate exactly at the observed minima and maxima and the superimposed box plots represent 25th percentile (lower), median (center), and 75th percentile (upper) with whiskers extending to data points within 1.5 × IQR of the quartiles.

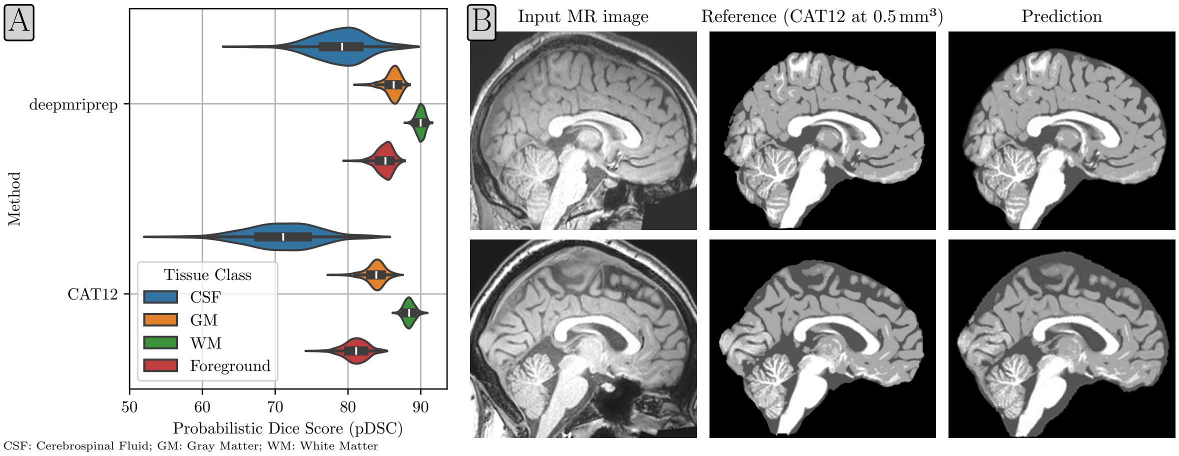

Supplementary Figure 2: Left: Probabilistic Dice scores of deepmriprep and CAT12 with respect to the cerebrospinal fluid (CSF), gray matter (GM), white matter (WM) and foreground (mean of CSF, GM and WM) across 685 MRIs from OpenNeuro-HD. Violin plot density traces terminate exactly at the observed minima and maxima and the superimposed box plots represent 25th percentile (lower), median (center), and 75th percentile (upper) with whiskers extending to data points within 1.5 × IQR of the quartiles. Right: Sagittal slice of the T_1-weighted MR image, the reference tissue map (CAT12 at $0.5\,\mathrm{mm}^3$ resolution) and the predicted tissue segmentation map in the sample which resulted in the lowest foreground Dice score for deepmriprep (first row) and CAT12 (second row).

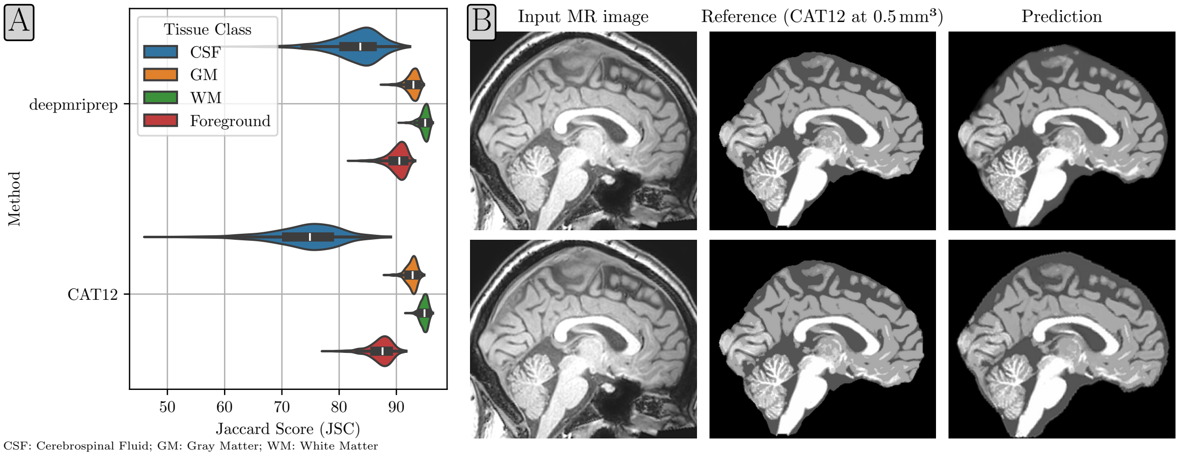

Supplementary Figure 3: Left: Jaccard scores of deepmriprep and CAT12 with respect to the cerebrospinal fluid (CSF), gray matter (GM), white matter (WM) and foreground (mean of CSF, GM and WM) across 685 MRIs from OpenNeuro-HD. Violin plot density traces terminate exactly at the observed minima and maxima and the superimposed box plots represent 25th percentile (lower), median (center), and 75th percentile (upper) with whiskers extending to data points within 1.5 × IQR of the quartiles. Right: Sagittal slice of the T_1-weighted MR image, the reference tissue map (CAT12 at $0.5\,\mathrm{mm}^3$ resolution) and the predicted tissue segmentation map in the sample which resulted in the lowest foreground Dice score for deepmriprep (first row) and CAT12 (second row).

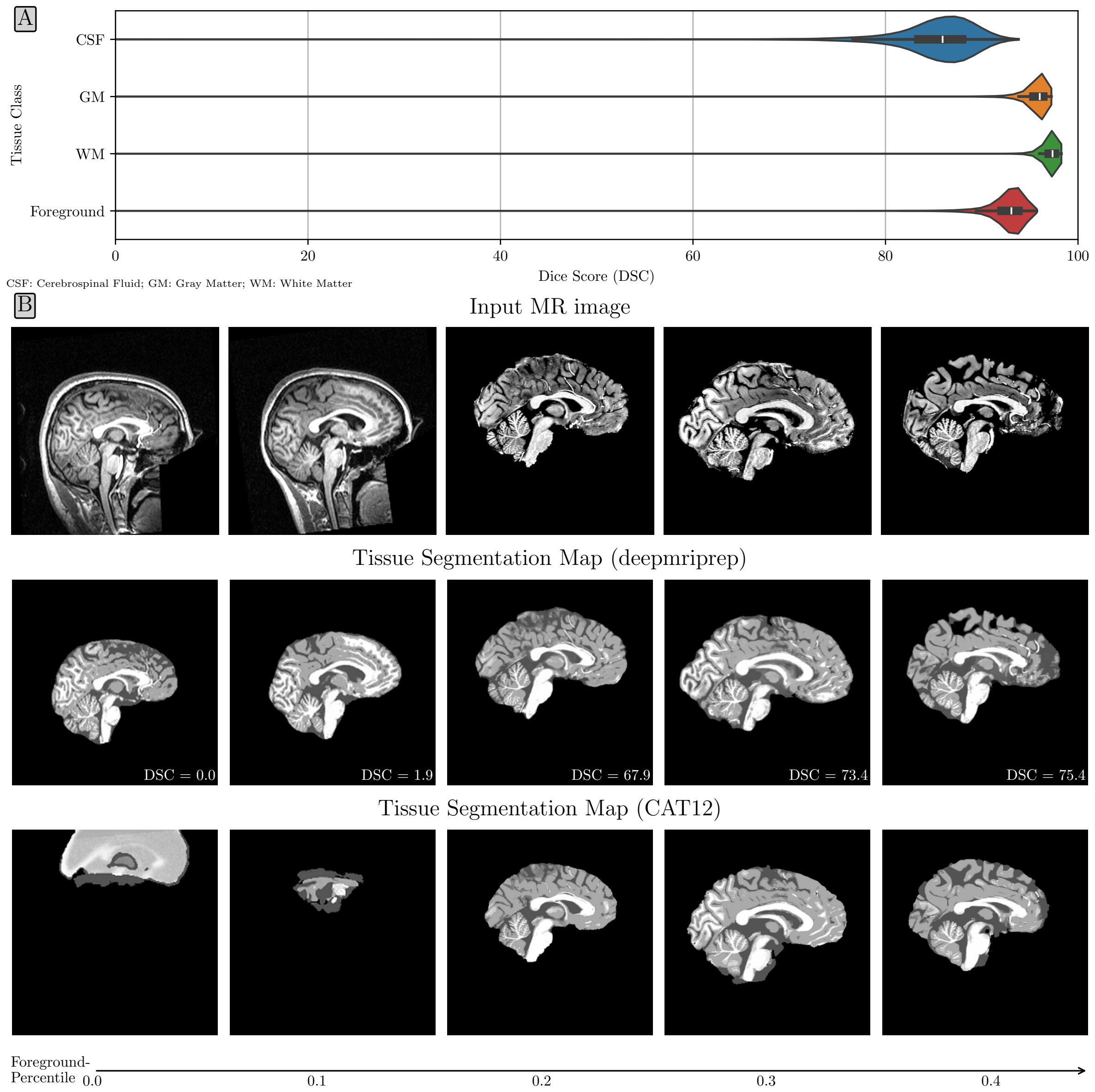

Supplementary Figure 4: A: Dice scores between deepmriprep and CAT12 across all 8,279 MRIs from OpenNeuro with respect to the cerebrospinal fluid (CSF), gray matter (GM), white matter (WM), and foreground (mean of CSF, GM and WM). Violin plot density traces terminate exactly at the observed minima and maxima and the superimposed box plots represent 25th percentile (lower), median (center), and 75th percentile (upper) with whiskers extending to data points within 1.5 × IQR of the quartiles. B: MRI input (first row) and tissue segmentation map (deepmriprep: second row, CAT12: third row) which resulted in the 0.0, 0.1, 0.2, 0.3 and 0.4 percentile foreground Dice scores across all 8,279 MRIs.

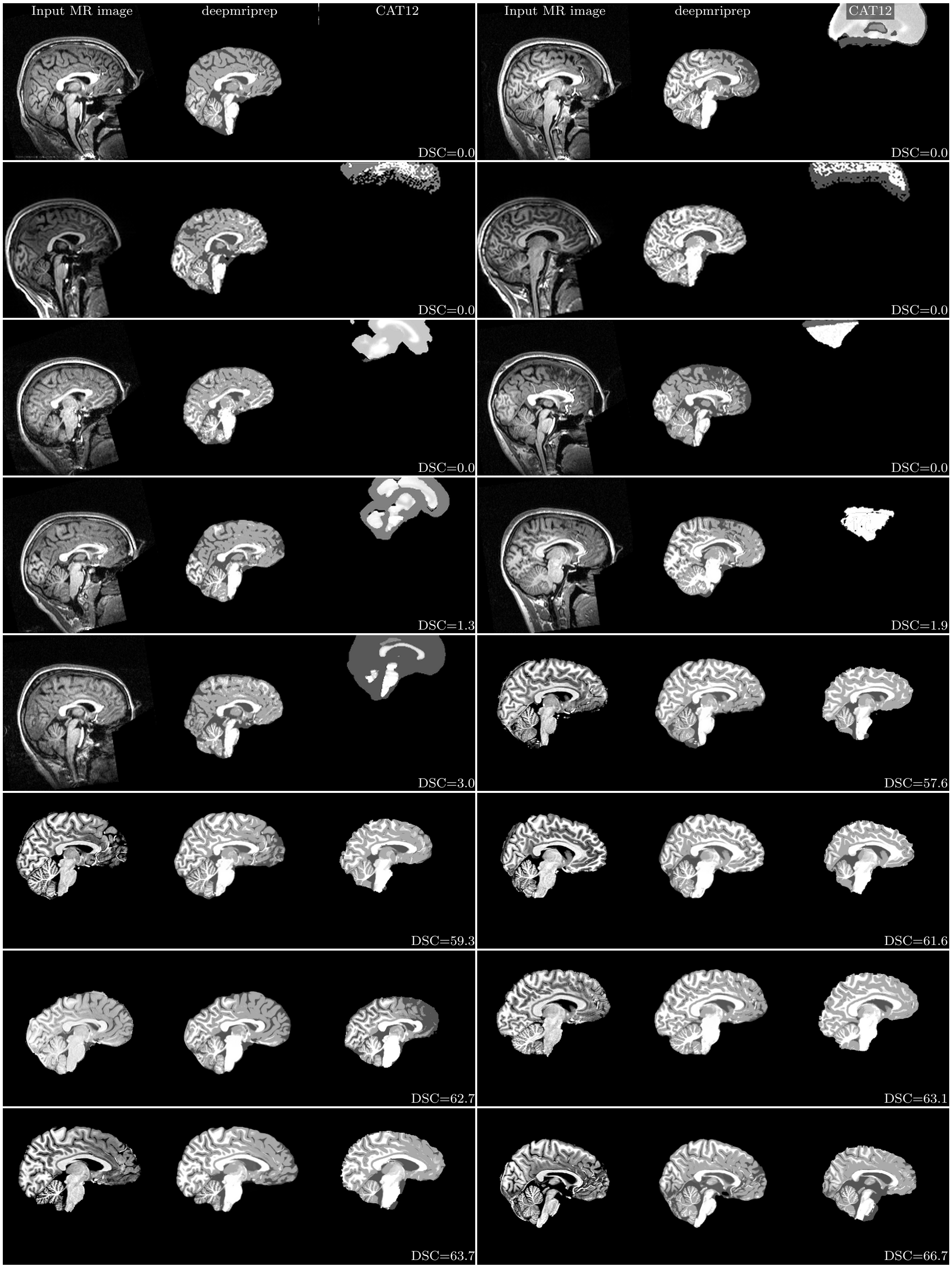

Supplementary Figure 5: 16 out of 8,279 MR images from OpenNeuro-Total which resulted in the tissue maps with the largest disagreement - meaning, lowest foreground Dice score - between deepmriprep and CAT12.

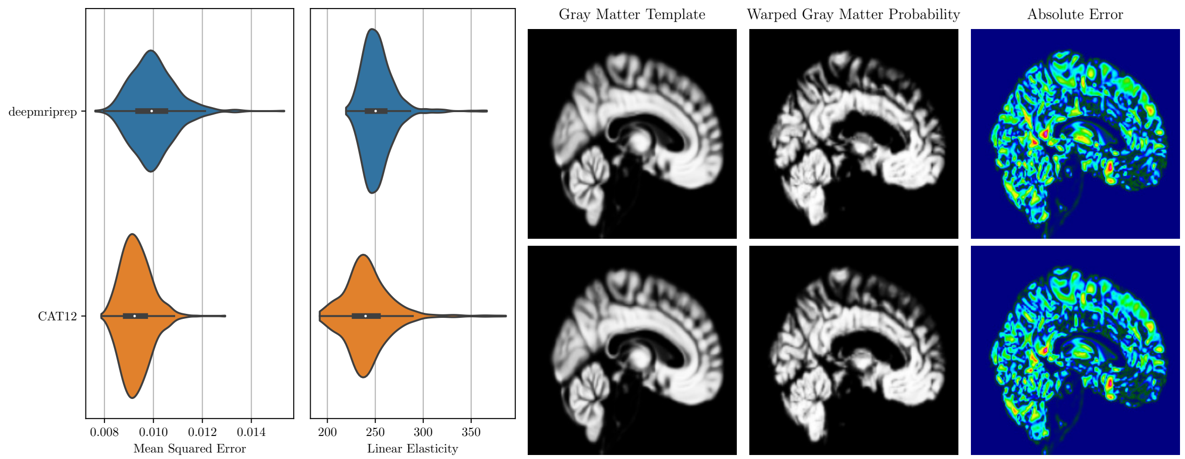

Supplementary Figure 6: Left: Image registration metrics Mean Squared Error and Linear Elasticity of deepmriprep and CAT12 across all 685 images in OpenNeuro-HD. Violin plot density traces terminate exactly at the observed minima and maxima and the superimposed box plots represent 25th percentile (lower), median (center), and 75th percentile (upper) with whiskers extending to data points within 1.5 $\times$ IQR of the quartiles. Right: Gray matter template, next to warped output and the resulting absolute error between template and output of deepmriprep (top) and CAT12 (bottom).

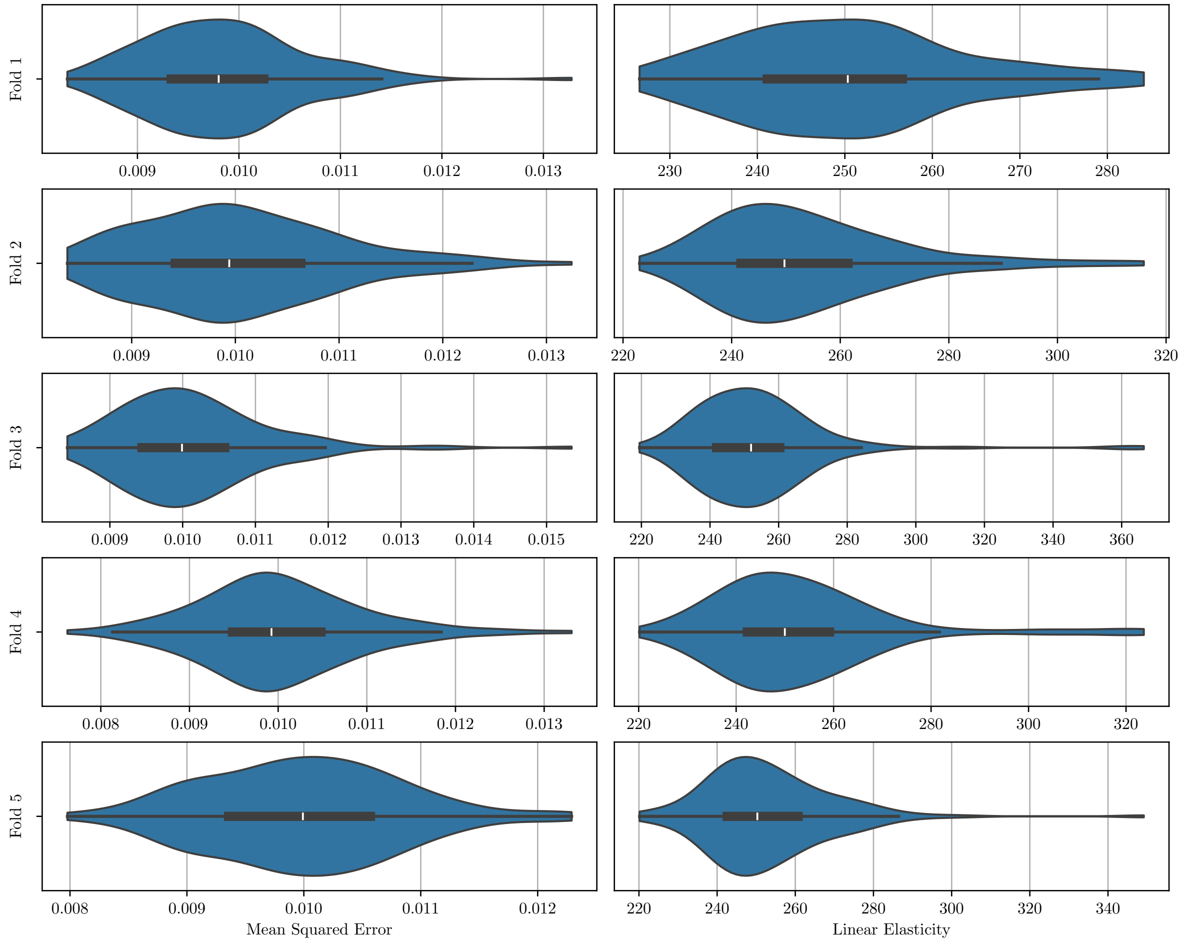

Supplementary Figure 7: Image registration metrics Mean Squared Error (left) and Linear Elasticity (right) of deepmriprep across 685 MRIs from OpenNeuro-HD splitted by validation folds. Violin plot density traces terminate exactly at the observed minima and maxima and the superimposed box plots represent 25th percentile (lower), median (center), and 75th percentile (upper) with whiskers extending to data points within 1.5 × IQR of the quartiles.

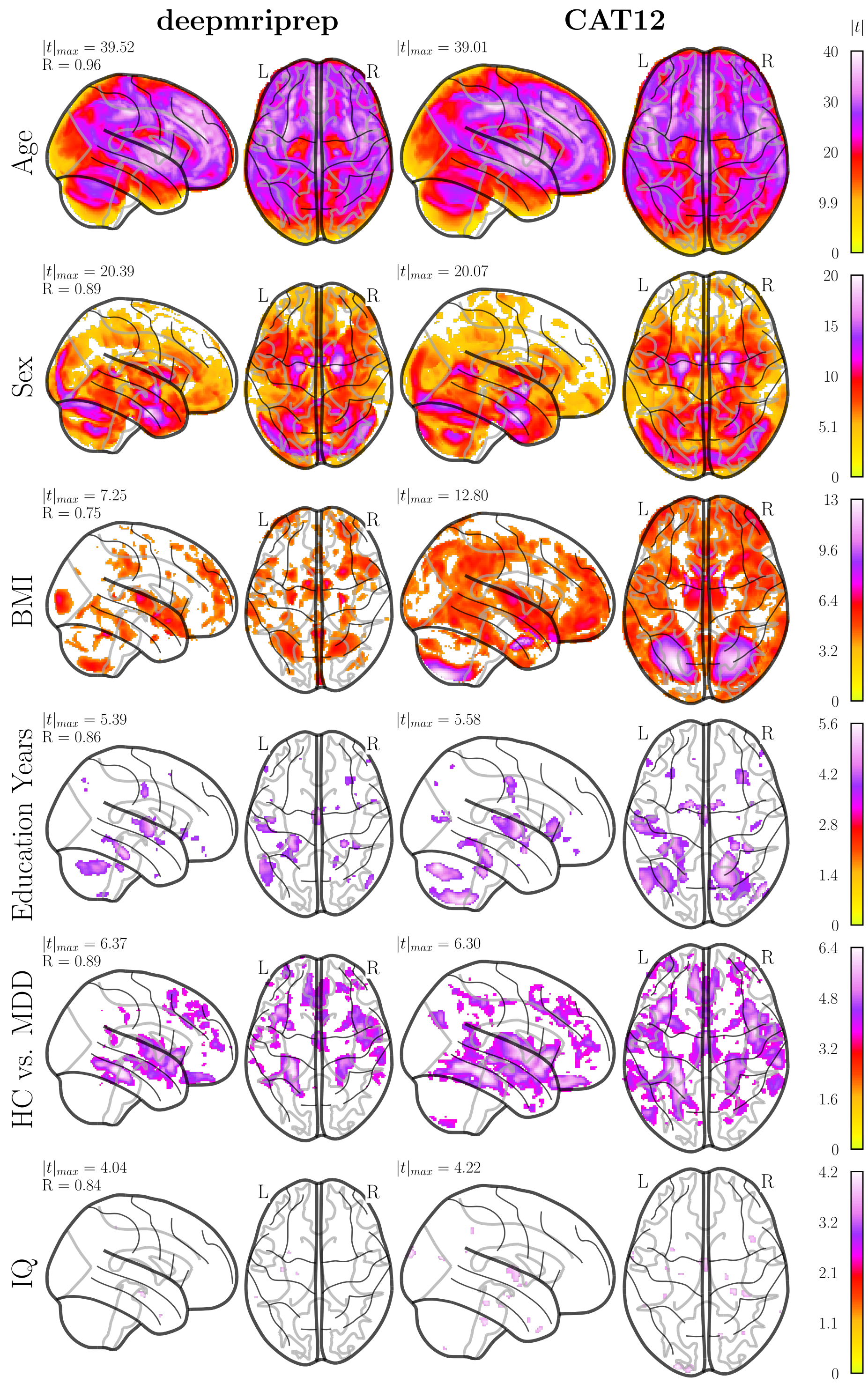

Supplementary Figure 8: Absolute, uncorrected t-scores of GLM analysis between gray matter volume and age, sex, body mass index (BMI), years of education, HC vs. MDD (healthy control vs. major depressive disorder) and intelligence quotient (IQ) based on deepmriprep- (left) and CAT12-preprocessing (right) thresholded at $p < 0.001$ (two-sided t-tests). The respective maximum values and correlation coefficients between deepmriprep and CAT12 are based on unthresholded absolute t-scores.

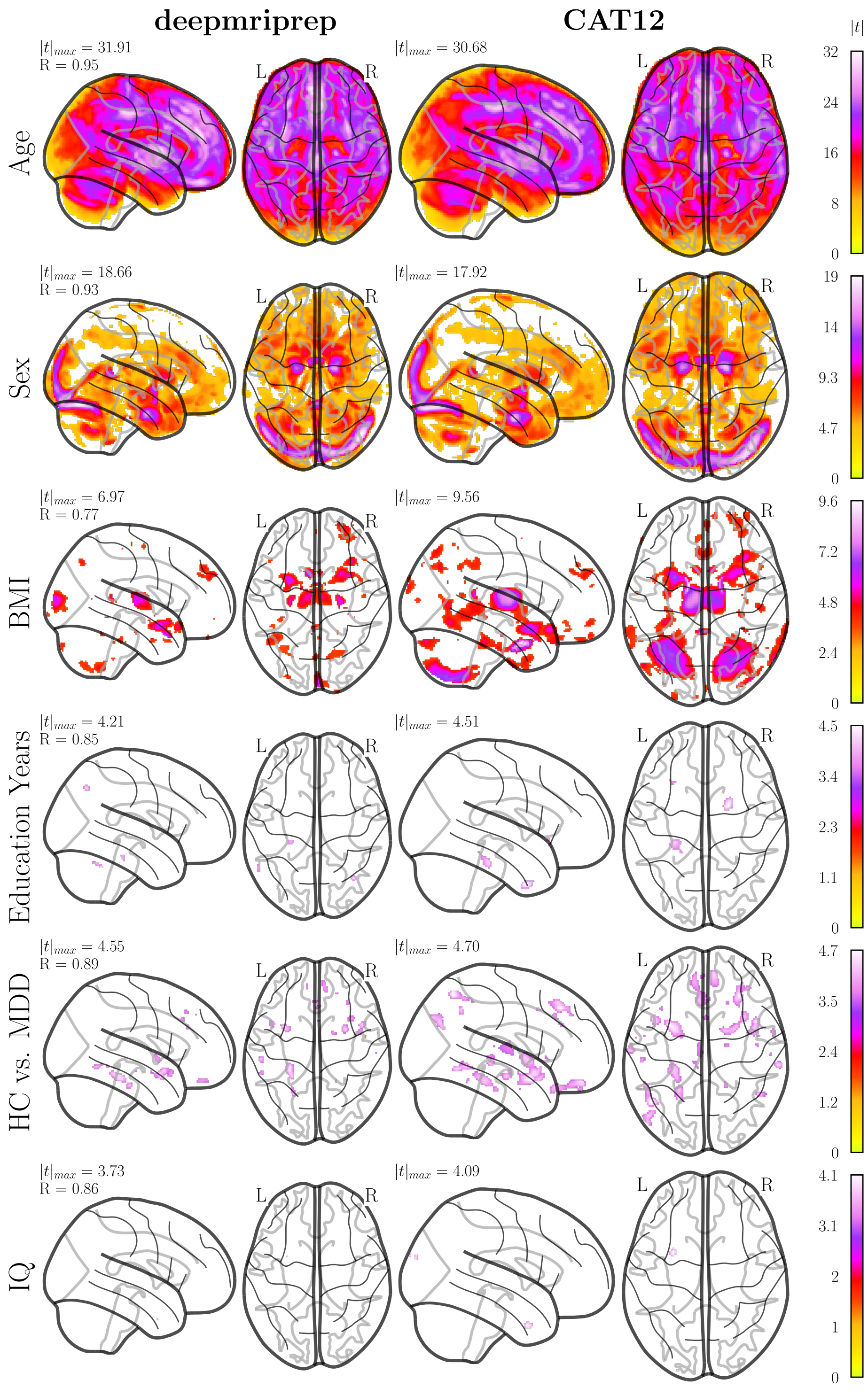

Supplementary Figure 9: Absolute, uncorrected t-scores of GLM analysis in the Marburg-Münster Affective Disorders Cohort Study (FOR2107/MACS) between gray matter volume and age, sex, body mass index (BMI), years of education, HC vs. MDD (healthy control vs. major depressive disorder) and intelligence quotient (IQ) based on deepmriprep- (left) and CAT12-preprocessing (right) thresholded at $p < 0.001$ (two-sided t-tests). The respective maximum values and correlation coefficients between deepmriprep and CAT12 are based on unthresholded absolute t-scores.

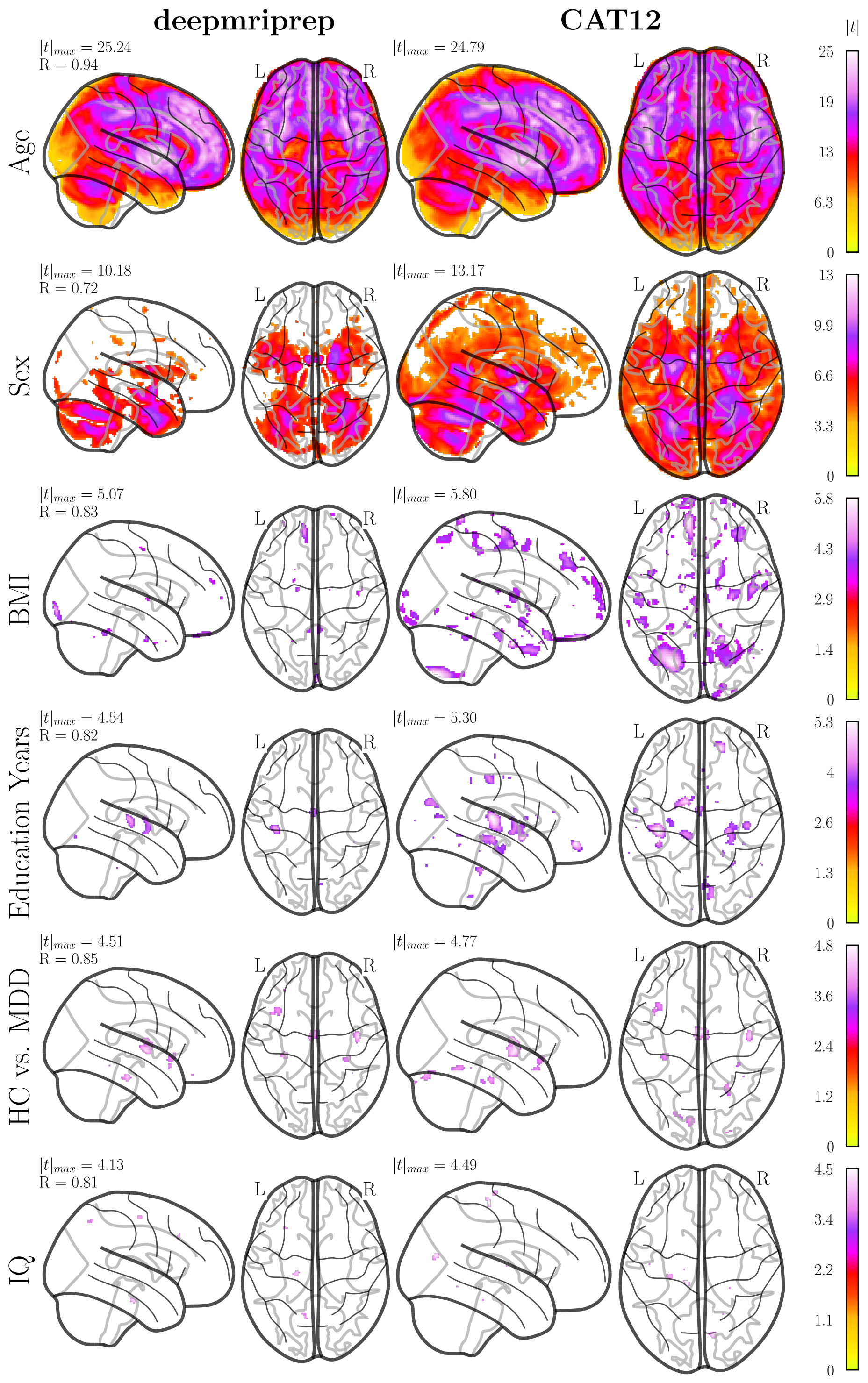

Supplementary Figure 10: Absolute, uncorrected t-scores of GLM analysis in the Münster Neuroimaging Cohort Study (MNC) between gray matter volume and age, sex, body mass index (BMI), years of education, HC vs. MDD (healthy control vs. major depressive disorder) and intelligence quotient (IQ) based on deepmriprep- (left) and CAT12-preprocessing (right) thresholded at $p < 0.001$ (two-sided t-tests). The respective maximum values and correlation coefficients between deepmriprep and CAT12 are based on unthresholded absolute t-scores.

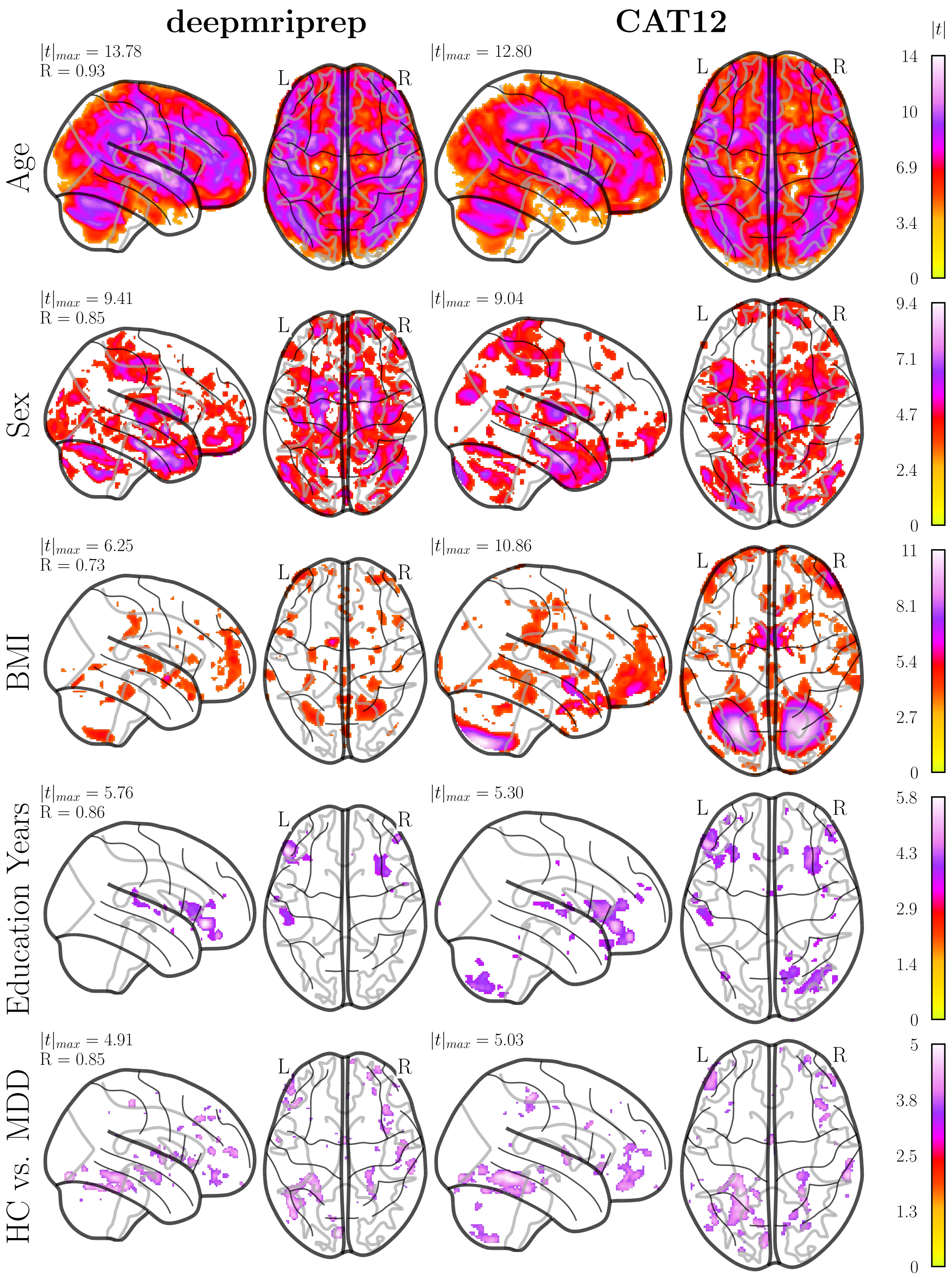

Supplementary Figure 11: Absolute, uncorrected t-scores of GLM analysis in the BiDirect between gray matter volume and age, sex, body mass index (BMI), years of education, HC vs. MDD (healthy control vs. major depressive disorder) and intelligence quotient (IQ) based on deepmriprep- (left) and CAT12-preprocessing (right) thresholded at $p < 0.001$ (two-sided t-tests). The respective maximum values and correlation coefficients between deepmriprep and CAT12 are based on unthresholded absolute t-scores.

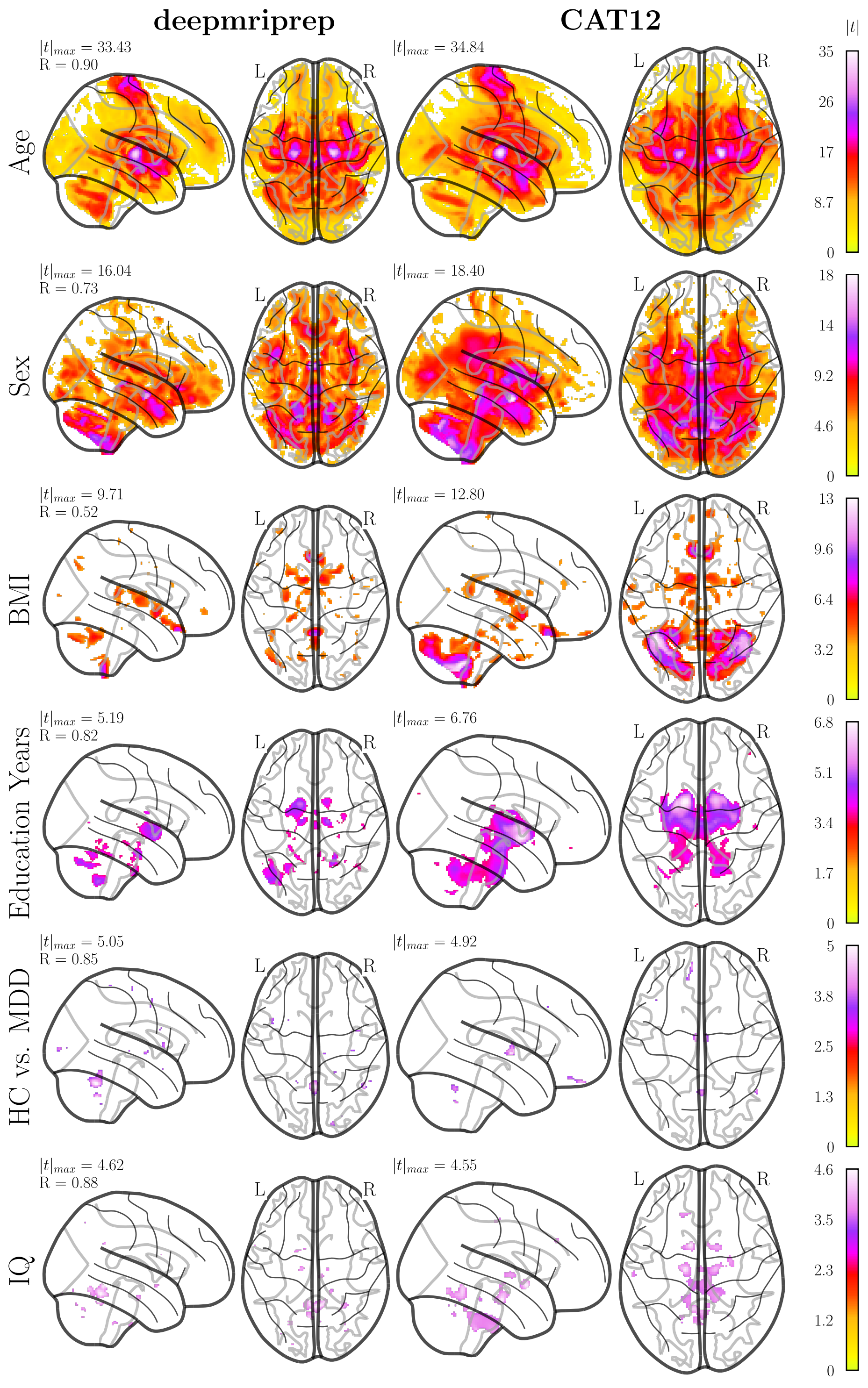

Supplementary Figure 12: Absolute, uncorrected t-scores of GLM analysis between white matter volume and age, sex, body mass index (BMI), years of education, HC vs. MDD (healthy control vs. major depressive disorder) and intelligence quotient (IQ) based on deepmriprep- (left) and CAT12-preprocessing (right) thresholded at $p < 0.001$ (two-sided t-tests). The respective maximum values and correlation coefficients between deepmriprep and CAT12 are based on unthresholded absolute t-scores.

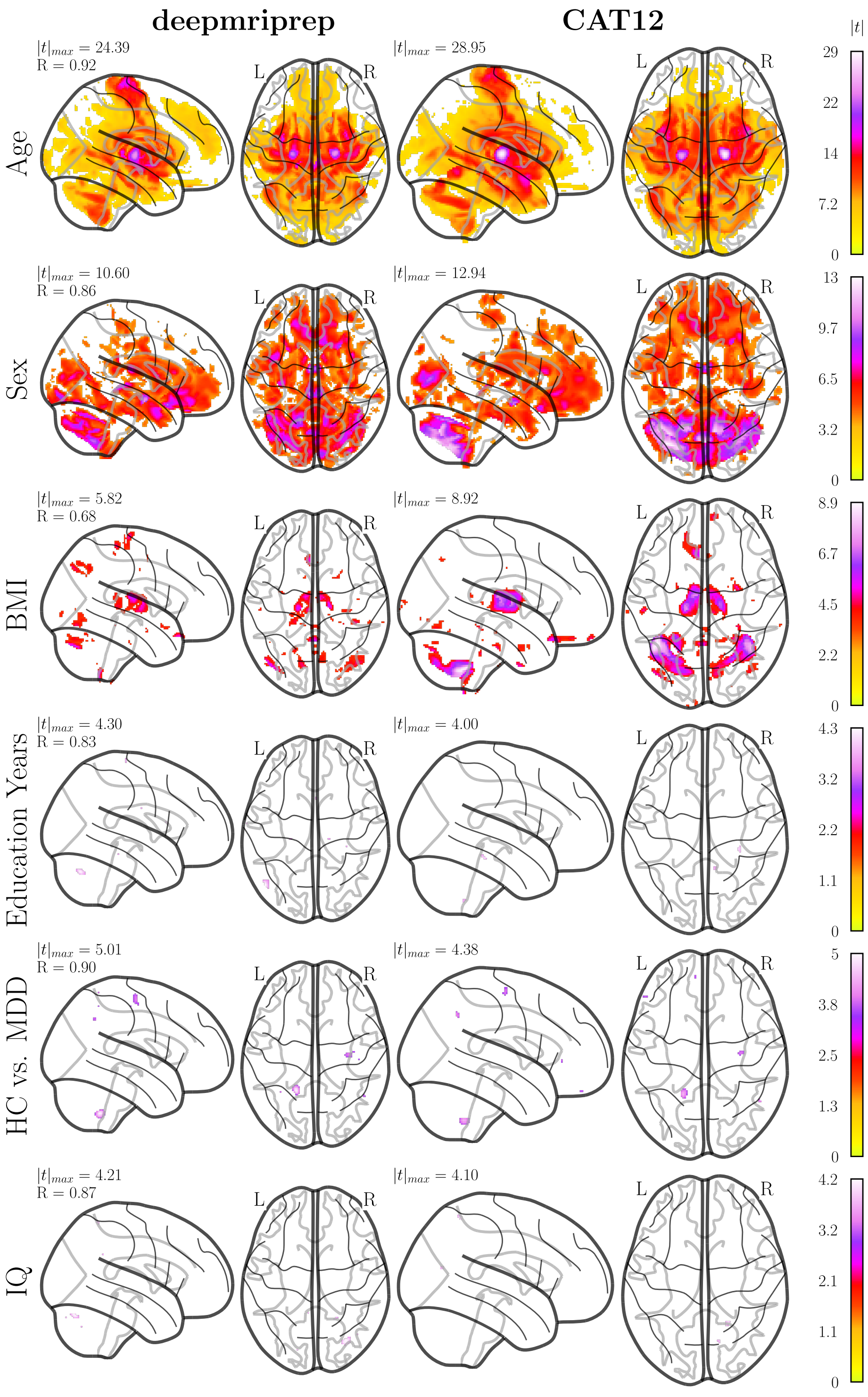

Supplementary Figure 13: Absolute, uncorrected t-scores of GLM analysis in the Marburg-Münster Affective Disorders Cohort Study (FOR2107/MACS) between white matter volume and age, sex, body mass index (BMI), years of education, HC vs. MDD (healthy control vs. major depressive disorder) and intelligence quotient (IQ) based on deepmriprep- (left) and CAT12-preprocessing (right) thresholded at $p < 0.001$ (two-sided t-tests). The respective maximum values and correlation coefficients between deepmriprep and CAT12 are based on unthresholded absolute t-scores.

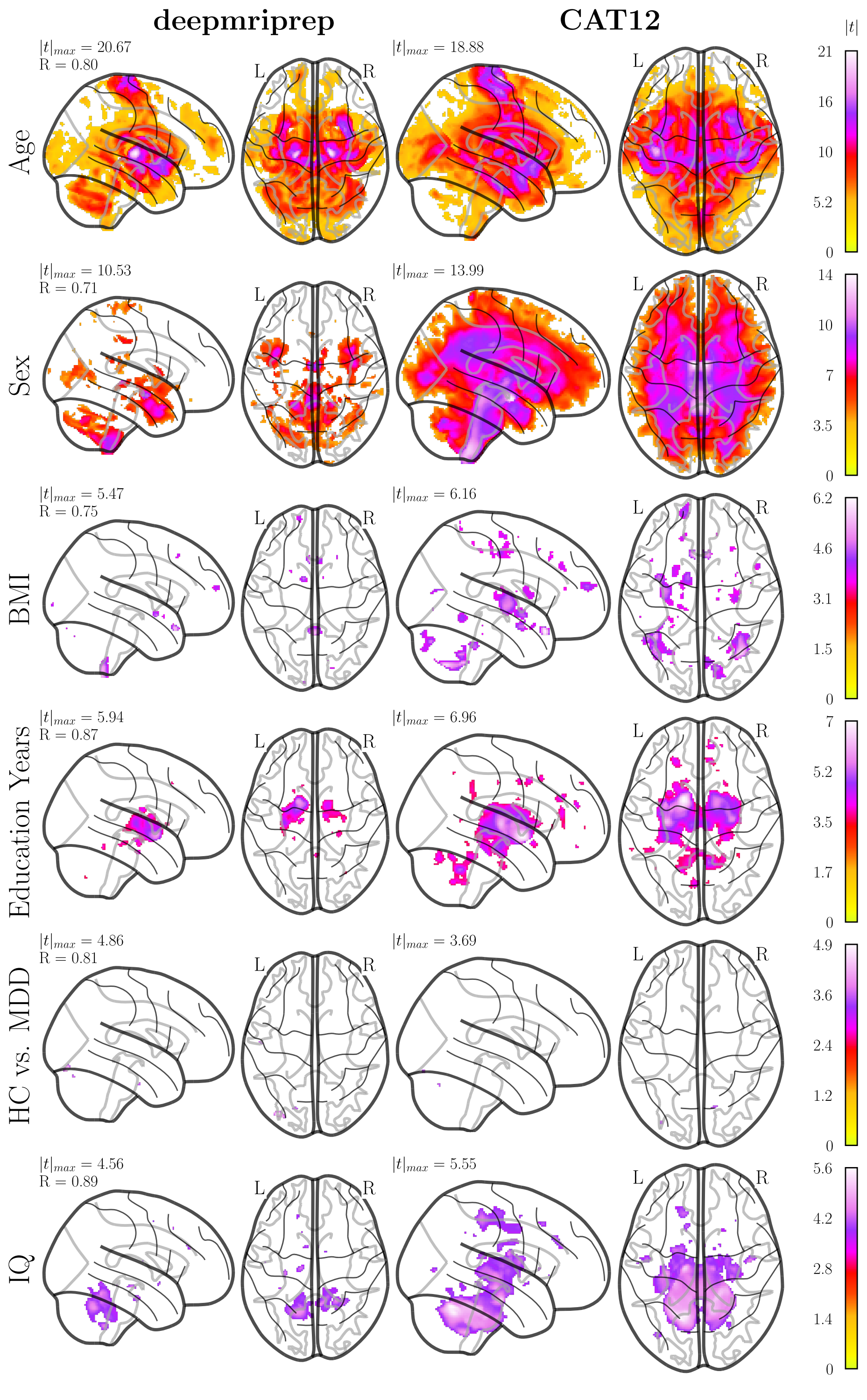

Supplementary Figure 14: Absolute, uncorrected t-scores of GLM analysis in the Münster Neuroimaging Cohort Study (MNC) between white matter volume and age, sex, body mass index (BMI), years of education, HC vs. MDD (healthy control vs. major depressive disorder) and intelligence quotient (IQ) based on deepmriprep- (left) and CAT12-preprocessing (right) thresholded at $p < 0.001$ (two-sided t-tests). The respective maximum values and correlation coefficients between deepmriprep and CAT12 are based on unthresholded absolute t-scores.

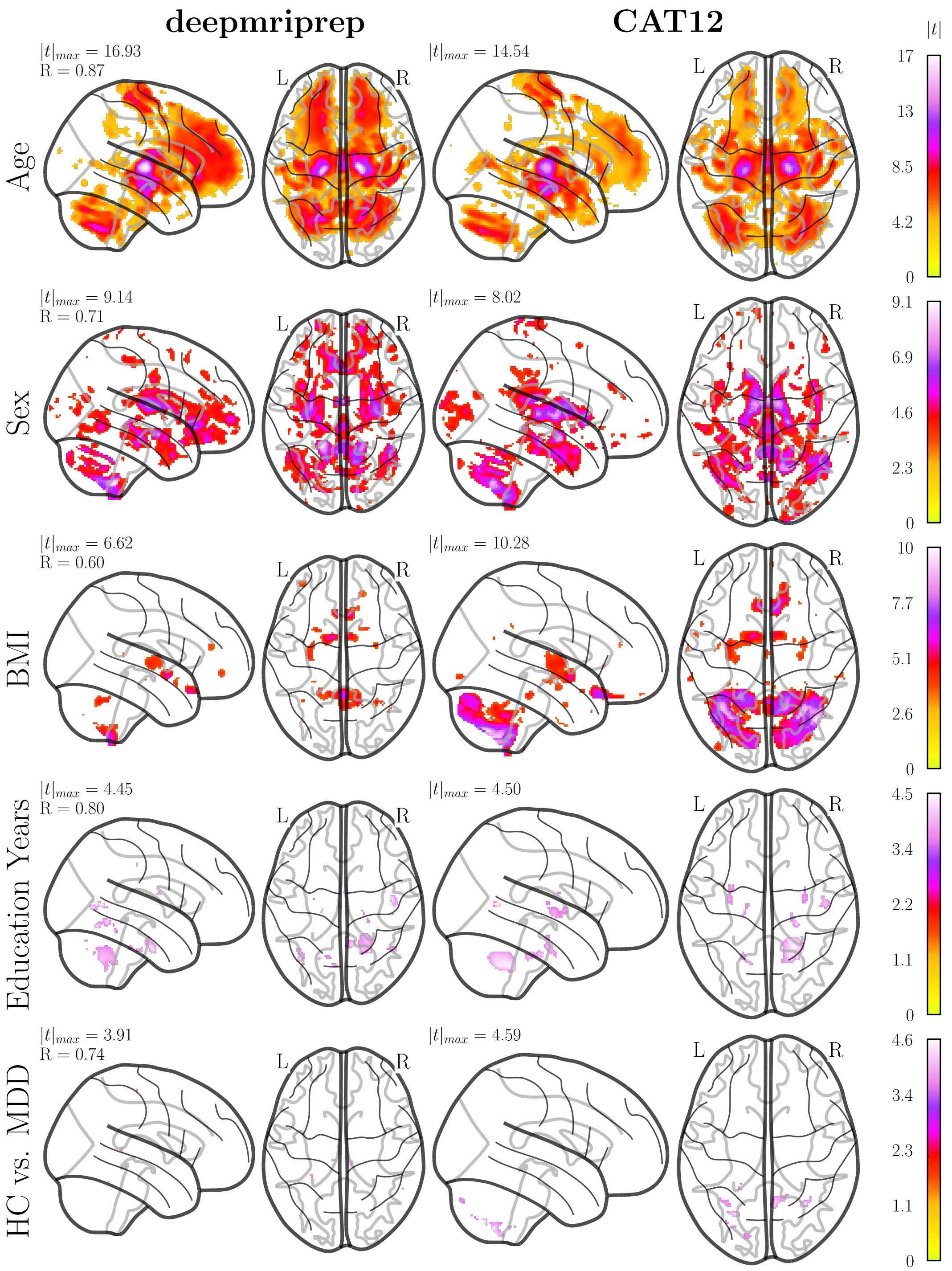

Supplementary Figure 15: Absolute, uncorrected t-scores of GLM analysis in the BiDirect between white matter volume and age, sex, body mass index (BMI), years of education, HC vs. MDD (healthy control vs. major depressive disorder) and intelligence quotient (IQ) based on deepmriprep- (left) and CAT12-preprocessing (right) thresholded at $p < 0.001$ (two-sided t-tests). The respective maximum values and correlation coefficients between deepmriprep and CAT12 are based on unthresholded absolute t-scores.

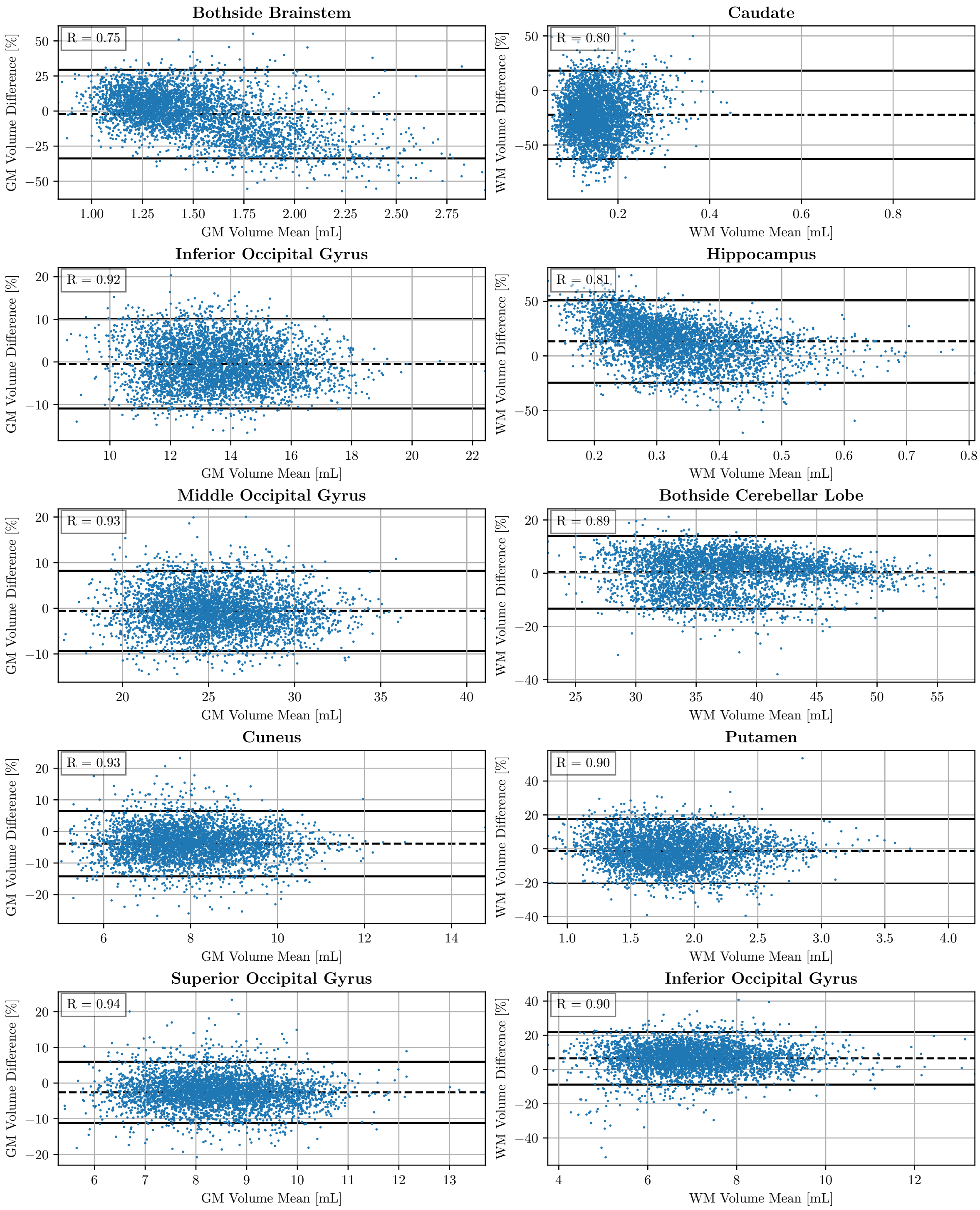

GM: Gray Matter; WM: White Matter

Supplementary Figure 16: Bland-Altman plots of the relative gray matter (left) and white matter (right) volume differences between deepmriprep and CAT12 across the mean volume in five regions of interest of the LPBA40 atlas with respective 95% confidence intervals (black). Shown are the five regions with the lowest correlation coefficients R of volume measurements between deepmriprep and CAT12.

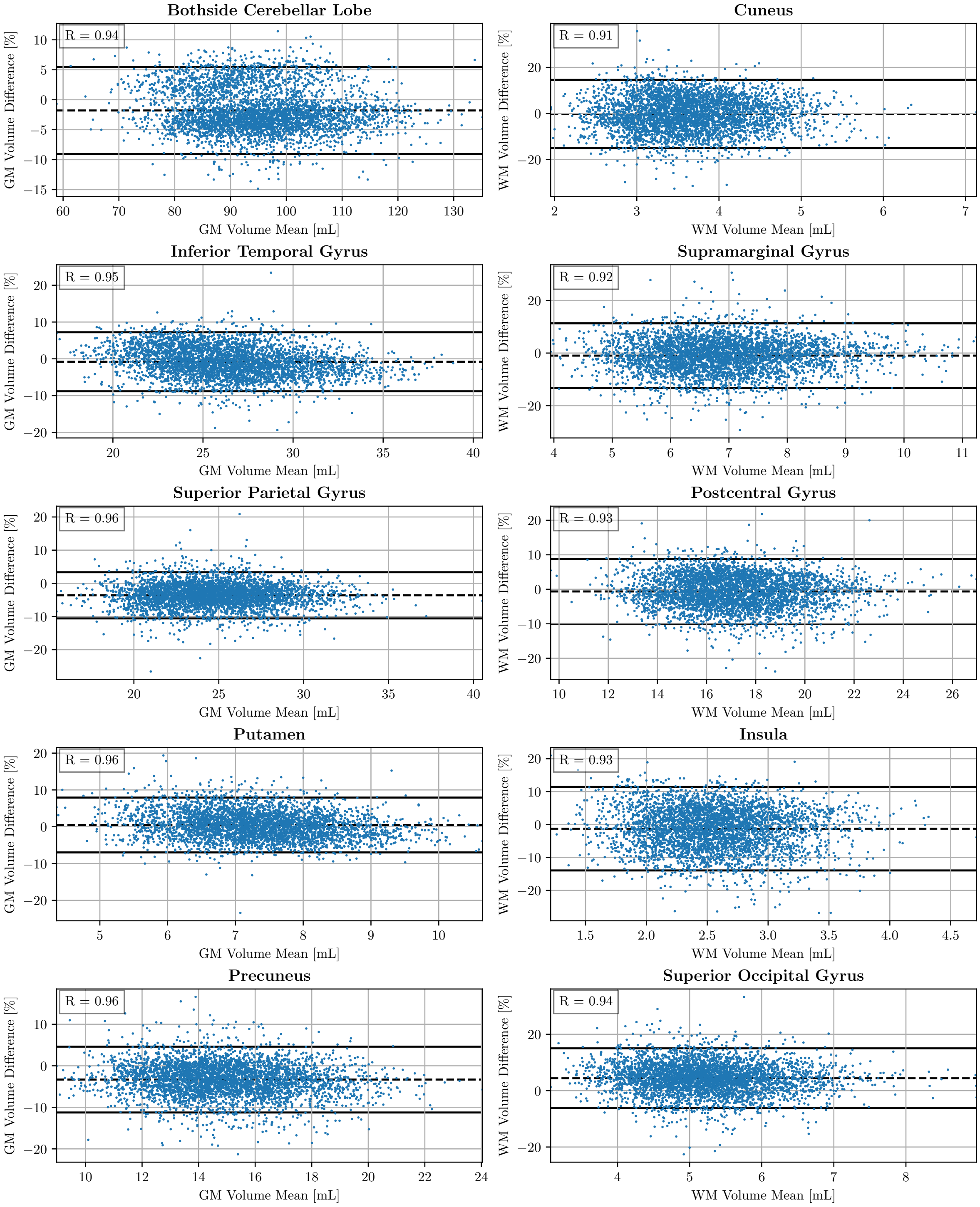

GM: Gray Matter; WM: White Matter

Supplementary Figure 17: Bland-Altman plots of the relative gray matter (left) and white matter (right) volume differences between deepmriprep and CAT12 across the mean volume in five regions of interest of the LPBA40 atlas with respective 95% confidence intervals (black). Shown are the five regions with the second lowest correlation coefficients R of volume measurements between deepmriprep and CAT12.

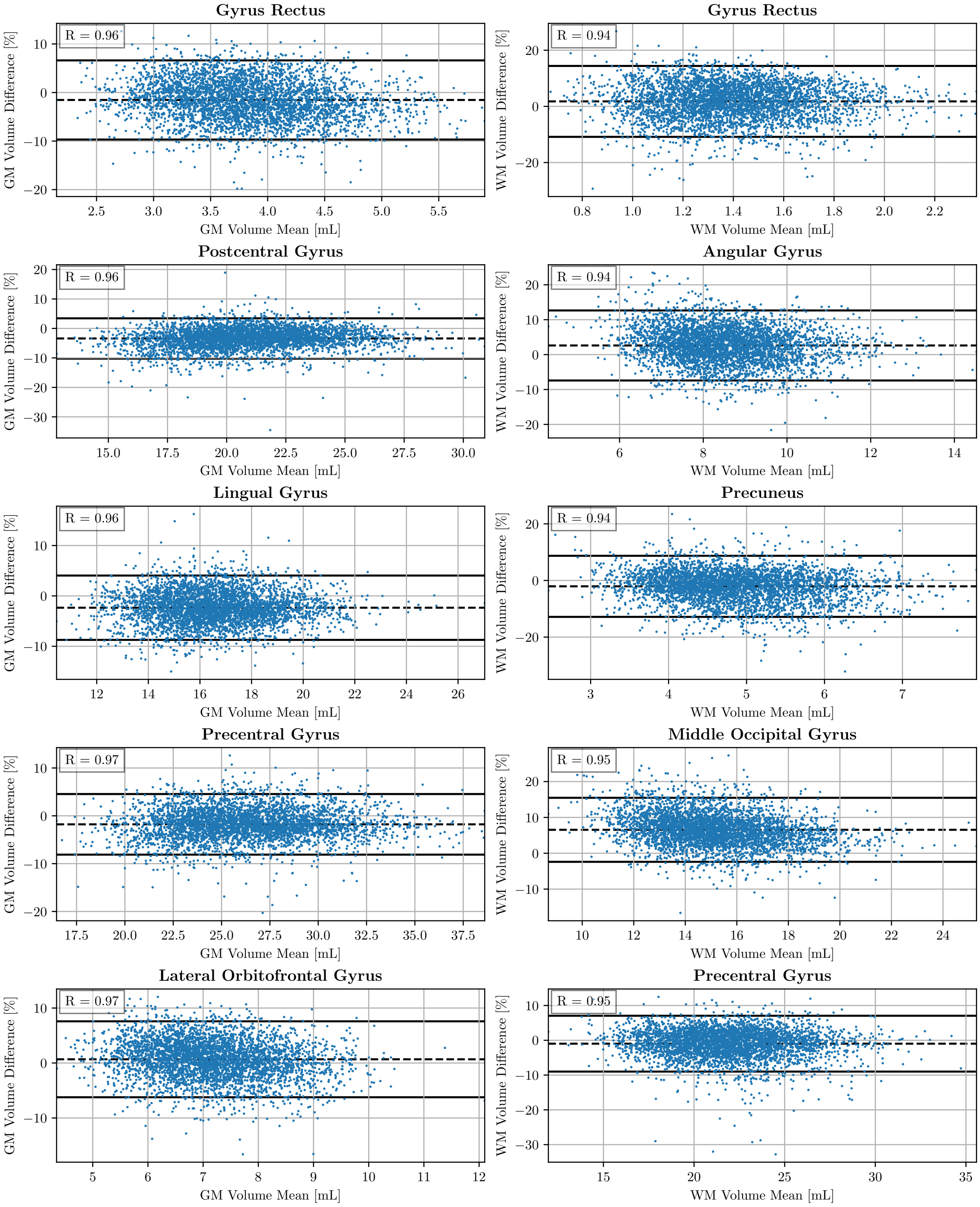

GM: Gray Matter; WM: White Matter

Supplementary Figure 18: Bland-Altman plots of the relative gray matter (left) and white matter (right) volume differences between deepmriprep and CAT12 across the mean volume in five regions of interest of the LPBA40 atlas with respective 95% confidence intervals (black). Shown are the five regions with the third lowest correlation coefficients R of volume measurements between deepmriprep and CAT12.

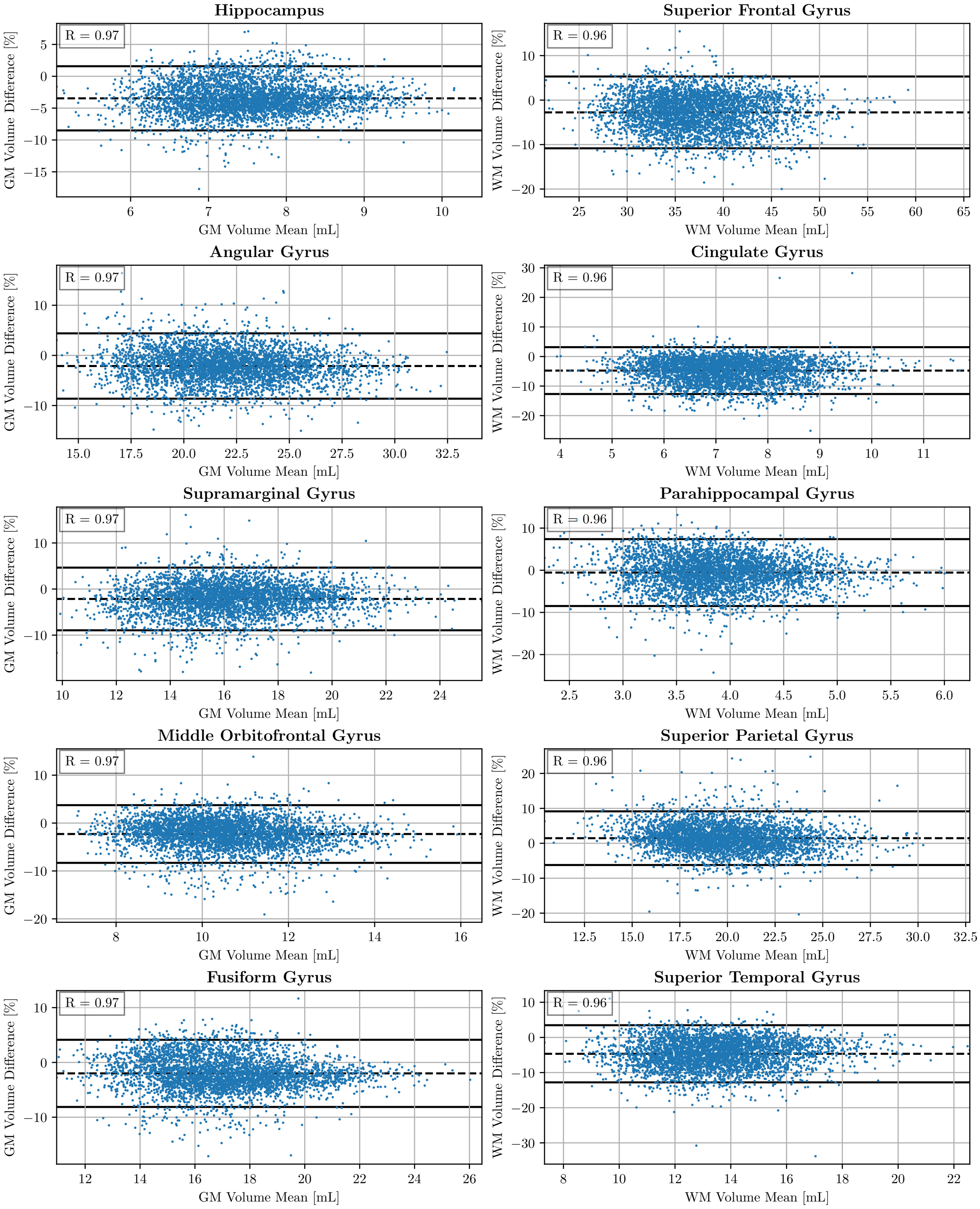

GM: Gray Matter; WM: White Matter

Supplementary Figure 19: Bland-Altman plots of the relative gray matter (left) and white matter (right) volume differences between deepmriprep and CAT12 across the mean volume in five regions of interest of the LPBA40 atlas with respective 95% confidence intervals (black). Shown are the five regions with the fourth lowest correlation coefficients R of volume measurements between deepmriprep and CAT12.

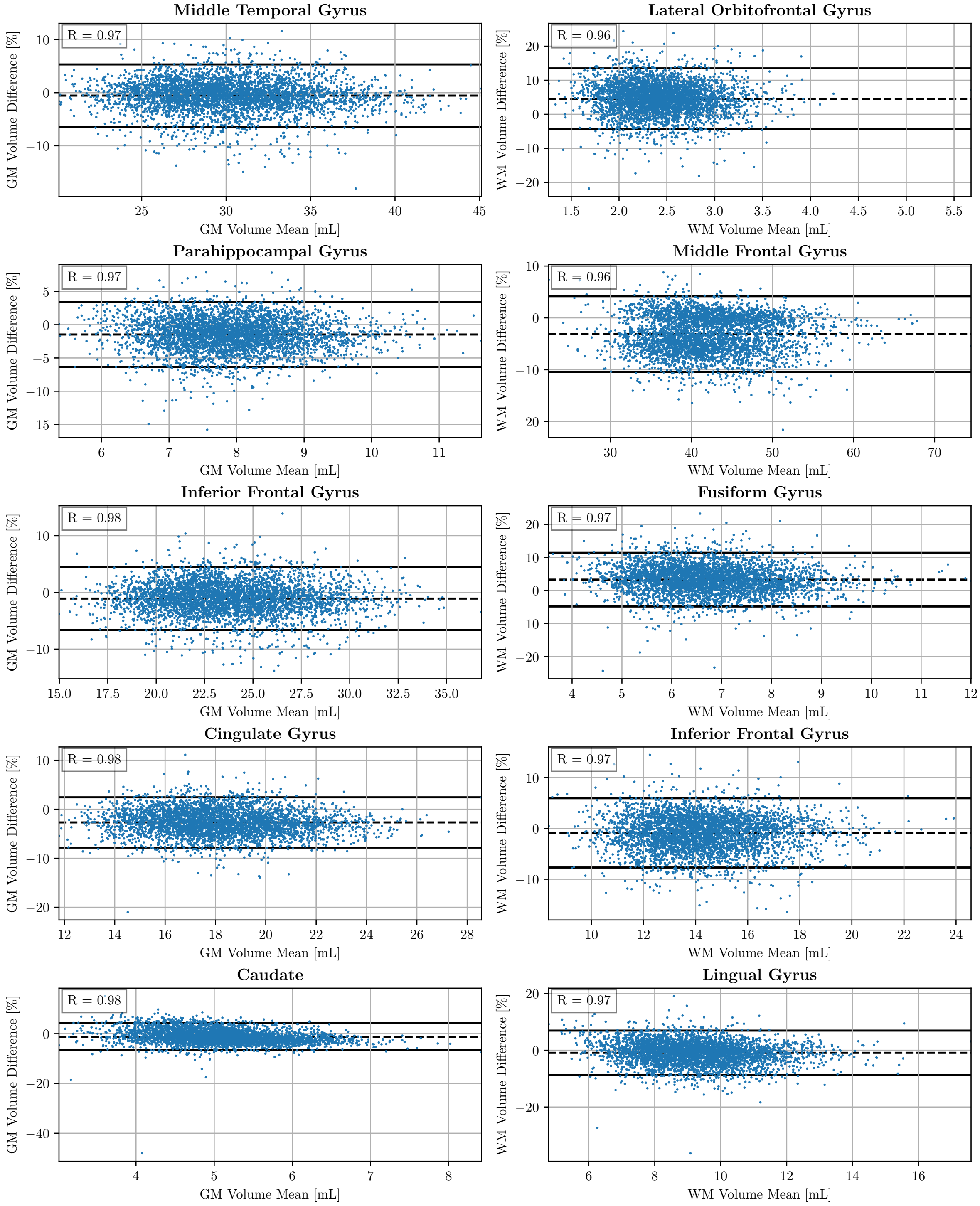

GM: Gray Matter; WM: White Matter

Supplementary Figure 20: Bland-Altman plots of the relative gray matter (left) and white matter (right) volume differences between deepmriprep and CAT12 across the mean volume in five regions of interest of the LPBA40 atlas with respective 95% confidence intervals (black). Shown are the five regions with the fifth lowest correlation coefficients R of volume measurements between deepmriprep and CAT12.

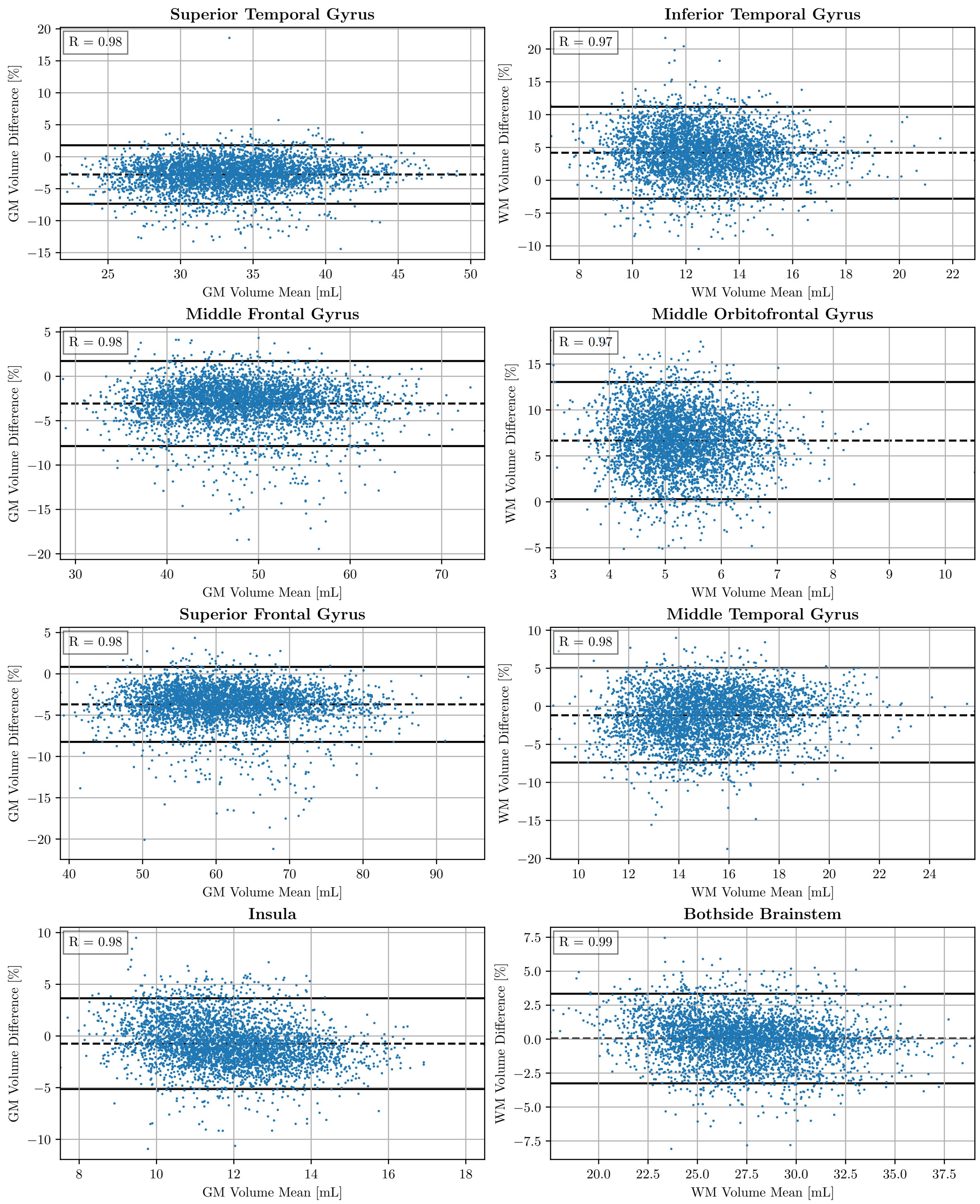

GM: Gray Matter; WM: White Matter

Supplementary Figure 21: Bland-Altman plots of the relative gray matter (left) and white matter (right) volume differences between deepmriprep and CAT12 across the mean volume in five regions of interest of the LPBA40 atlas with respective 95% confidence intervals (black). Shown are the five regions with the highest correlation coefficients R of volume measurements between deepmriprep and CAT12.

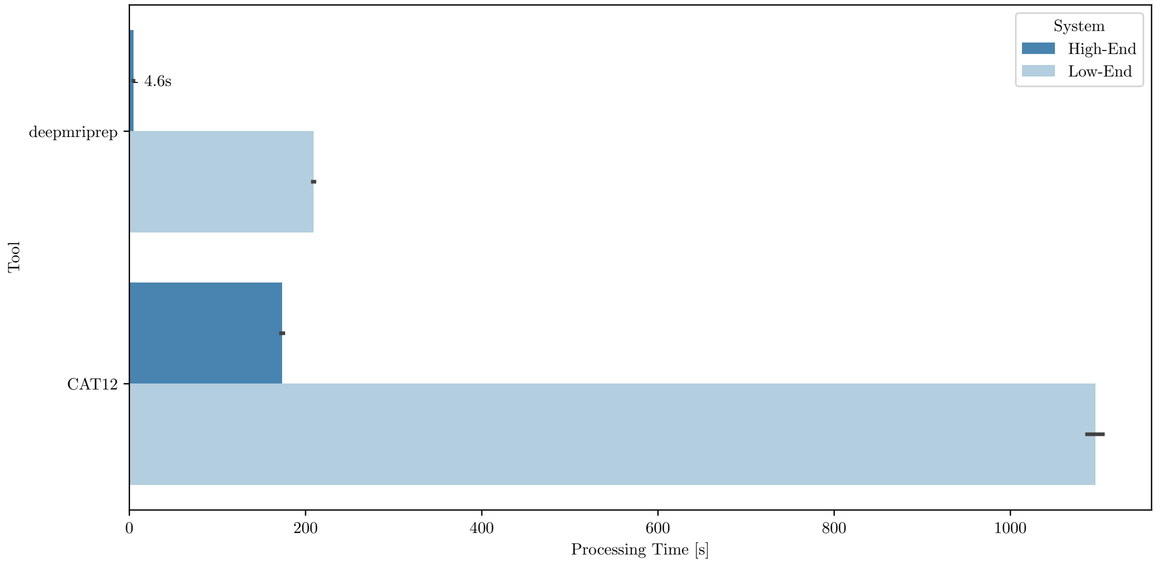

Supplementary Figure 22: Average processing time in seconds s of deepmriprep and CAT12 across 8,279 MRIs from OpenNeuro-Total. The high-end system utilizes a AMD Ryzen 9 5950X, a NVIDIA GeForce RTX 3090 Graphics Processing Unit (GPU) and 128GB of memory while the low-end system uses a Intel i7-8565U without a dedicated GPU and 16GB of memory. Error bars indicate the respective 95% confidence interval.

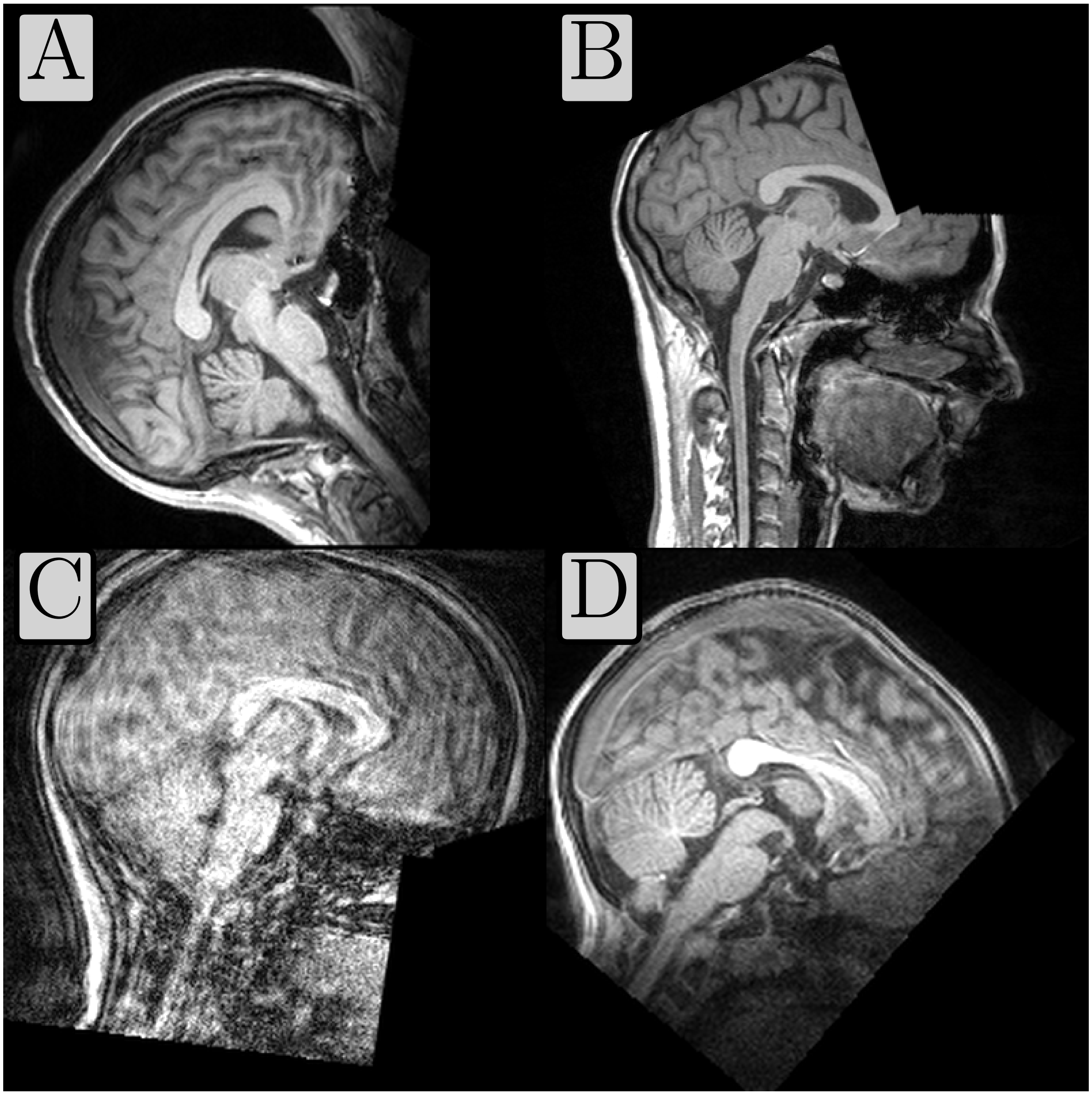

Supplementary Figure 23: Image dropouts caused by erroneous orientation (A), and improper masking (B) in OpenNeuro-Total and strong motion artifacts (C) and improper masking (D) in OpenNeuro-Kids.

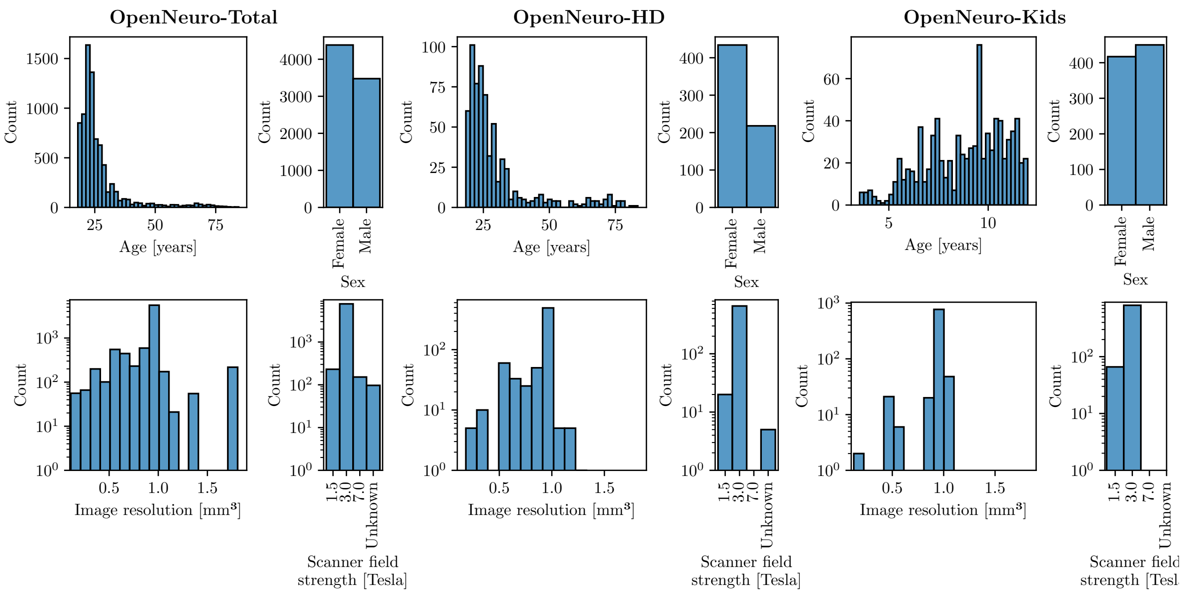

Supplementary Figure 24: Distribution of age, sex, image resolution, and scanner field strength across 8,279 subjects from OpenNeuro-Total (left), 685 subjects from OpenNeuro-HD (center), and 867 subjects from OpenNeuro-Kids (right).

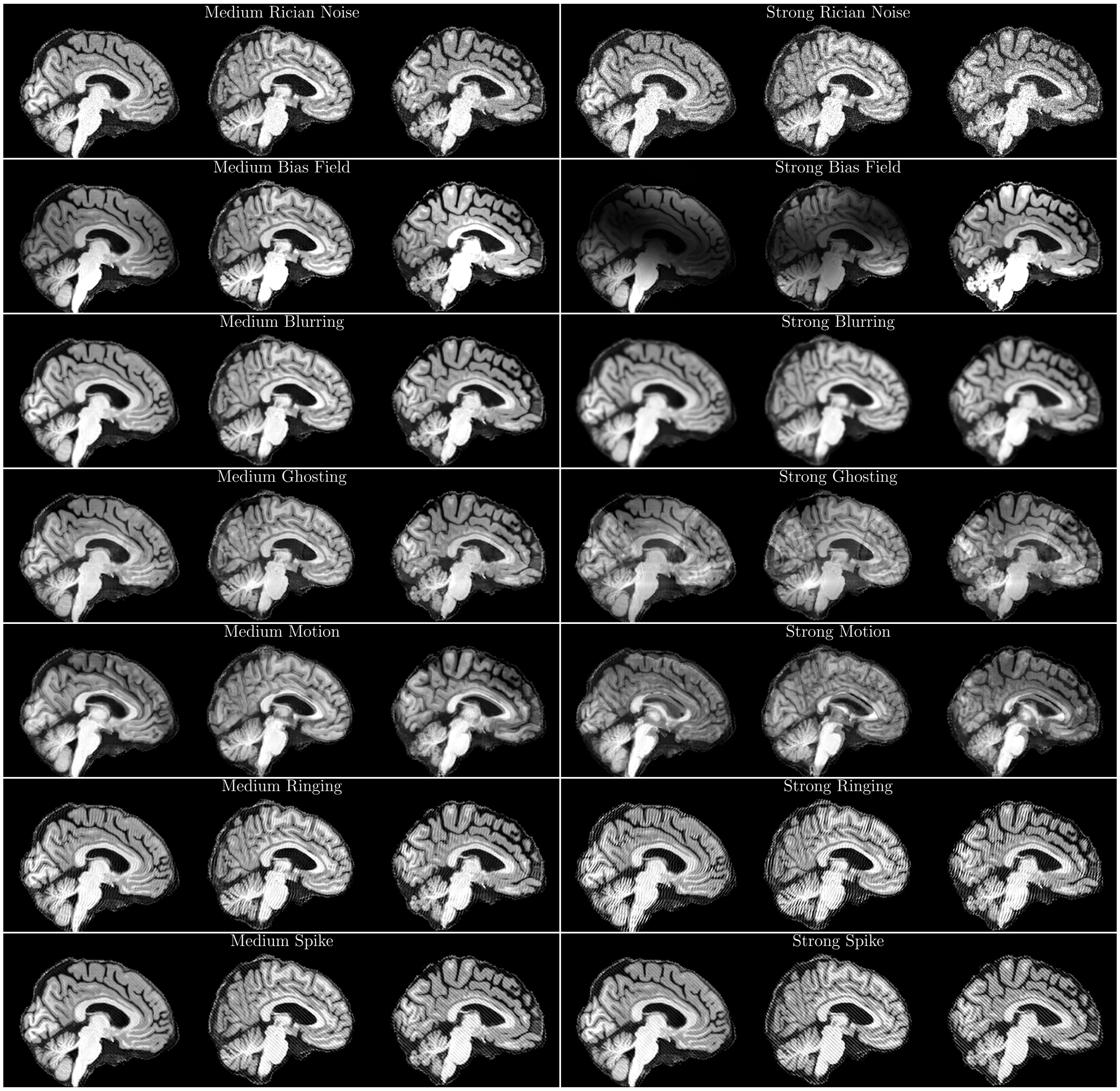

Supplementary Figure 25: Three example images of the Synthetic Atrophy dataset with medium image artifacts (left) and strong image artifacts (right).

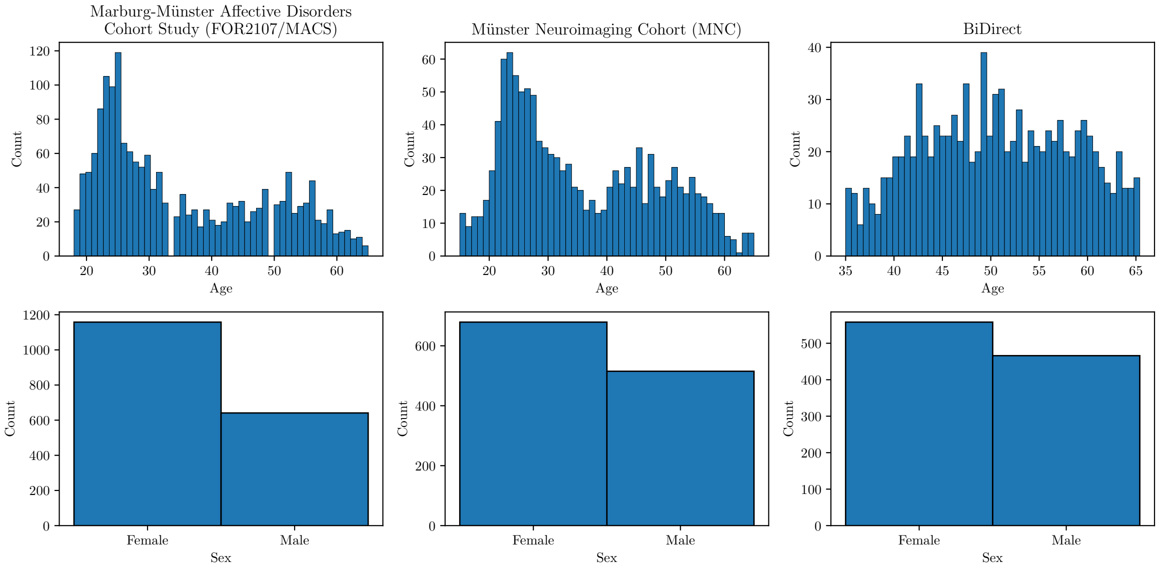

Supplementary Figure 26: Distribution of age and sex across 1,799 subjects from the Marburg-Münster Affective Disorders Cohort Study (left), 1,194 samples from the Münster Neuroimaging Cohort, and 1,024 subjects from the BiDirect cohort (right).

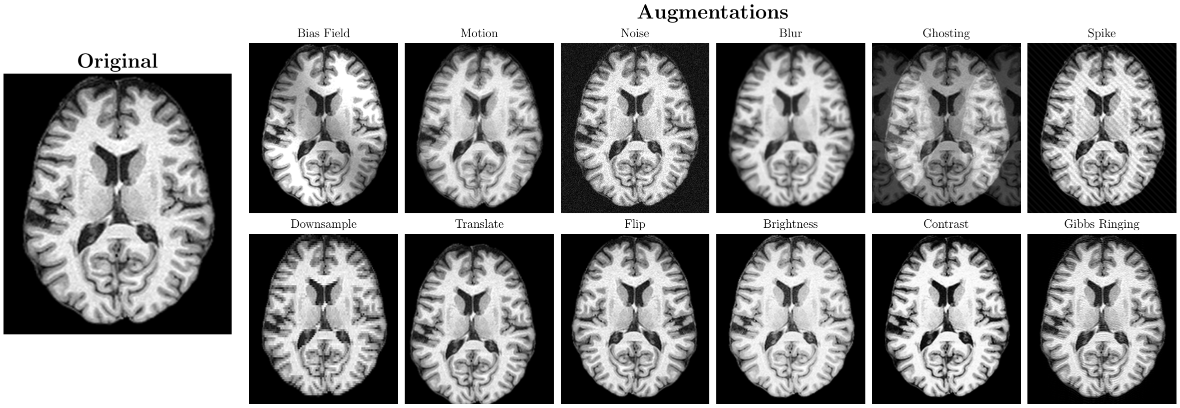

Supplementary Figure 27: Axial slice of an example image (left) and the respective slice after the respective augmentation was applied (right).

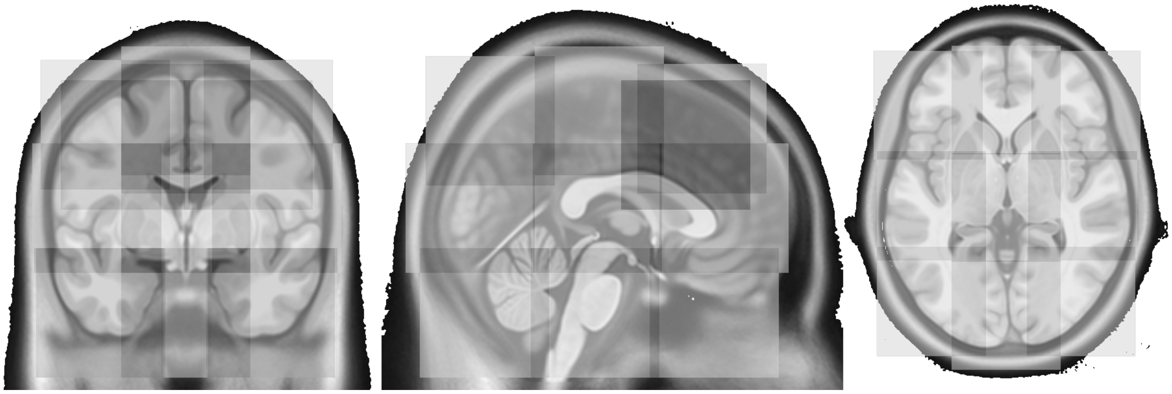

Supplementary Figure 28: Coronal (left), sagittal (center), and axial slice (right) of the 128x128x128 voxel patches resulting from the optimized patch positioning. For reference, the T1 template of CAT12, upsampled to the utilized resolution of 0.5 mm, is shown in the background.

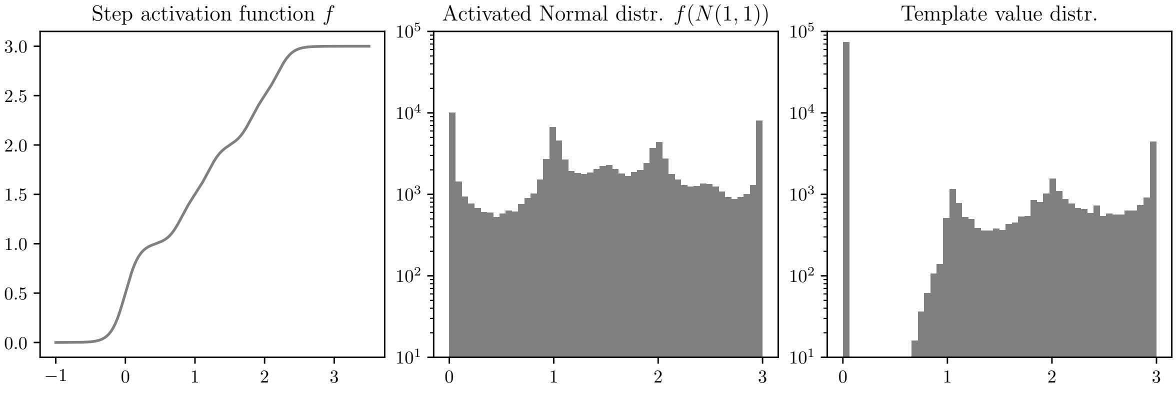

Supplementary Figure 29: Left: Step activation function f which maps logits to tissue values between 0 and 3. Center: Activation function f applied to 100,000 values drawn from the normal distribution $N(1,1)$. Right: Tissue value distribution in the MNI template.

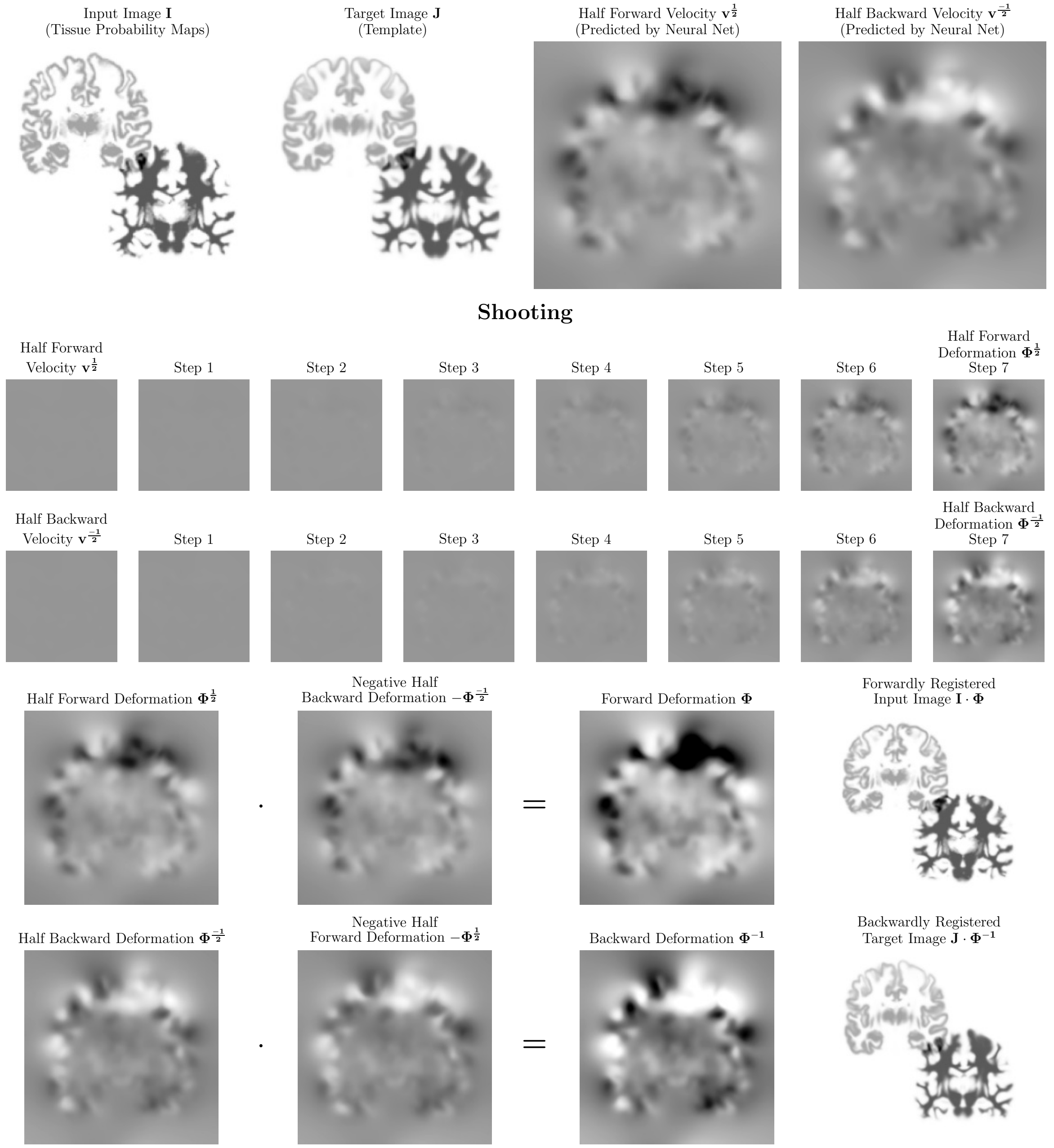

Supplementary Figure 30: Visualization of diffeomorphic image registration with neural networks. Top: Tissue probability map and registration template (gray matter top left, white matter bottom right) and velocity fields, which are predicted by the neural network. Center: Stepwise integration (Shooting) of the scaled velocity fields to arrive at the half deformation fields. Bottom: Composition of half deformation fields to form the full forward and backward deformation field. The tissue map is moved into template space (and vice versa) by applying these full deformation fields.

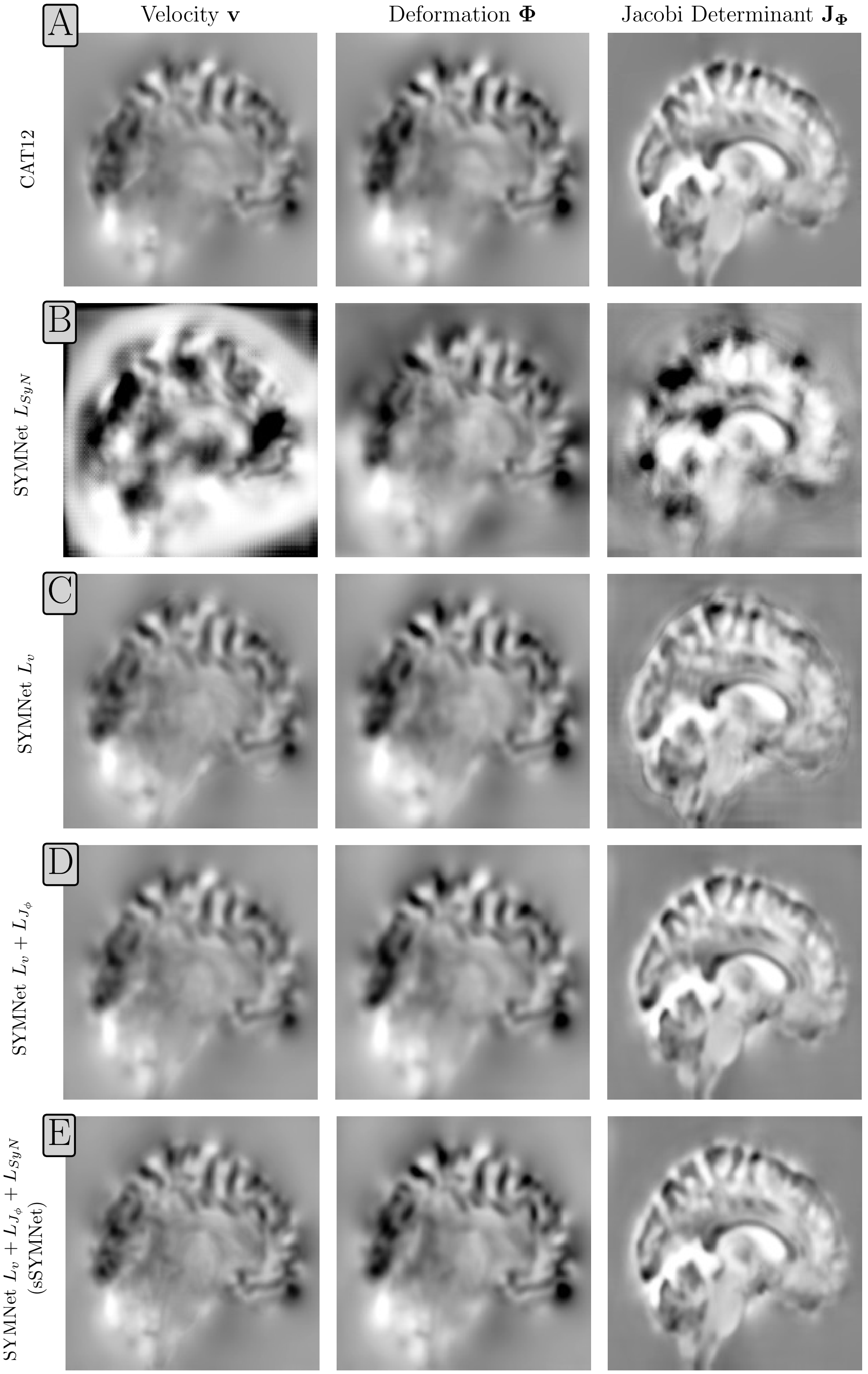

Supplementary Figure 31: Sagittal displacements in the velocity field, deformation field, and Jacobi determinant of one example image produced by CAT12 (A) and four variants of SYMNet trained with different loss functions (B-E).

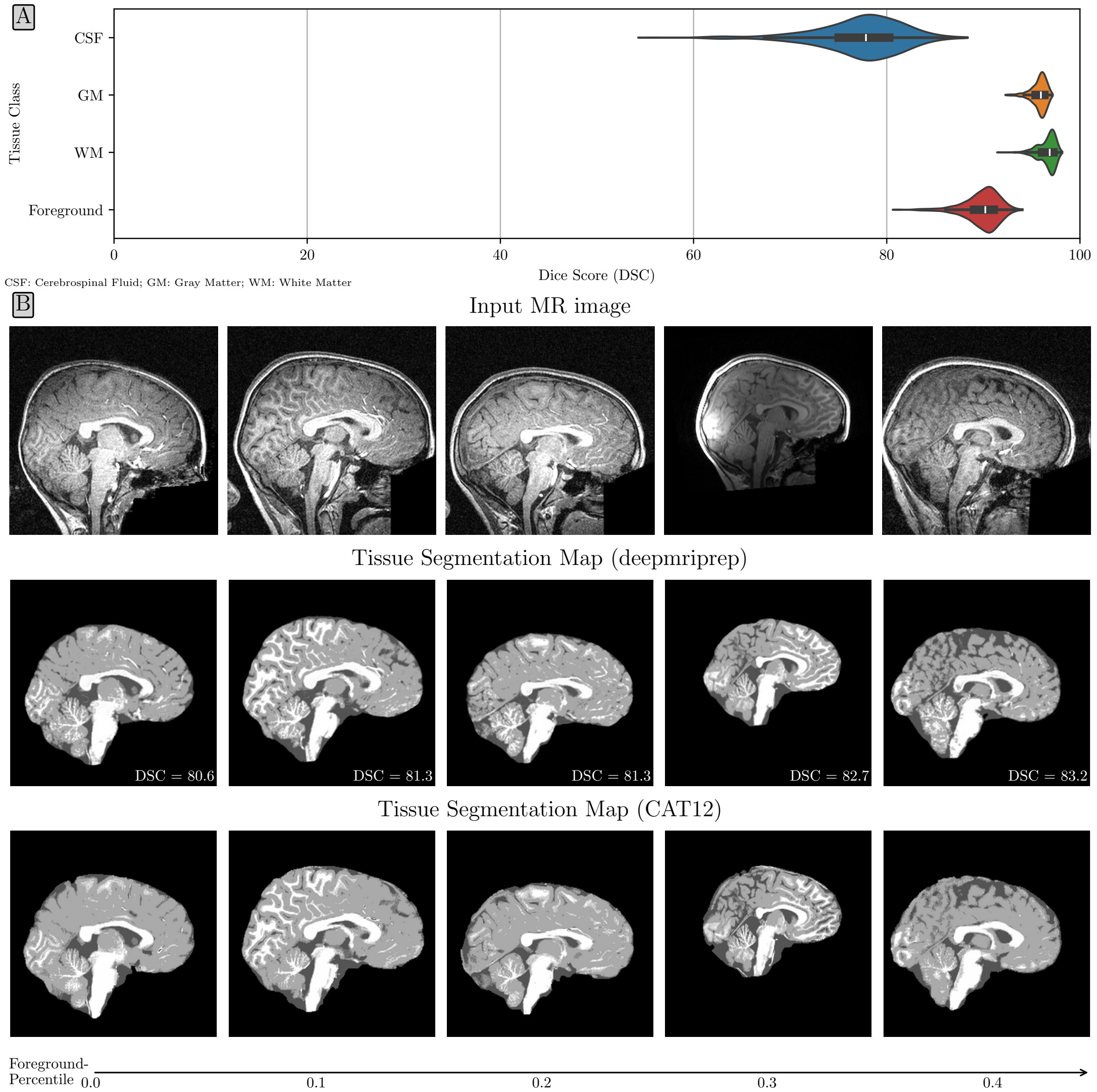

Supplementary Figure 32: Top: Dice scores between deepmriprep and CAT12 across 867 images from OpenNeuro-Kids with respect to the cerebrospinal fluid (CSF), gray matter (GM), white matter (WM), and foreground (mean of CSF, GM and WM). Bottom: MR image input (first row) and tissue segmentation map (deepmriprep: second row, CAT12: third row) which resulted in the 0.0, 0.1, 0.2, 0.3 and 0.4 percentile foreground Dice scores across all 867 images.

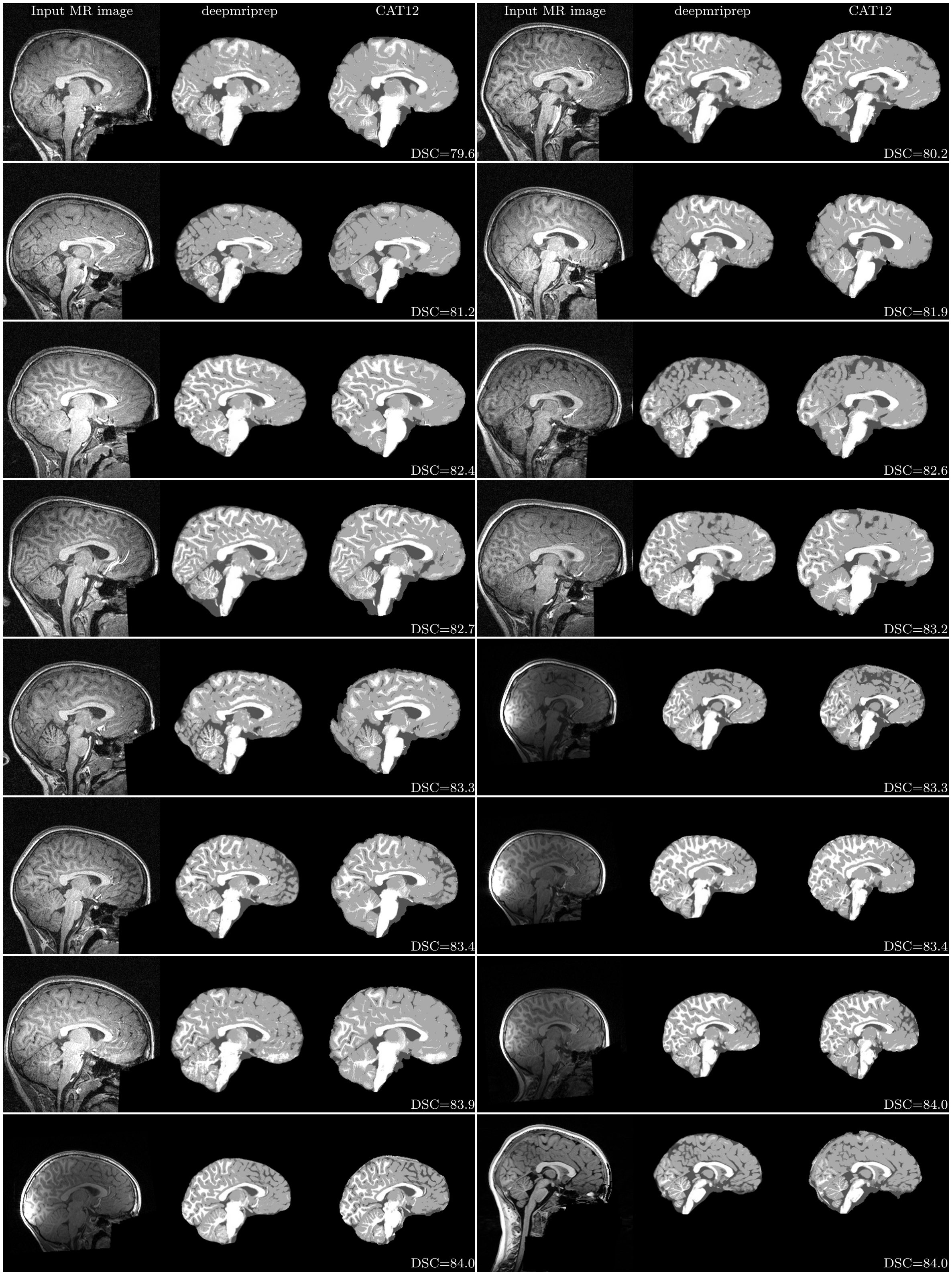

Supplementary Figure 33: 16 out of 867 MR images from OpenNeuro-Kids which resulted in the tissue maps with the largest disagreement - meaning, lowest foreground Dice score - between deepmriprep and CAT12.

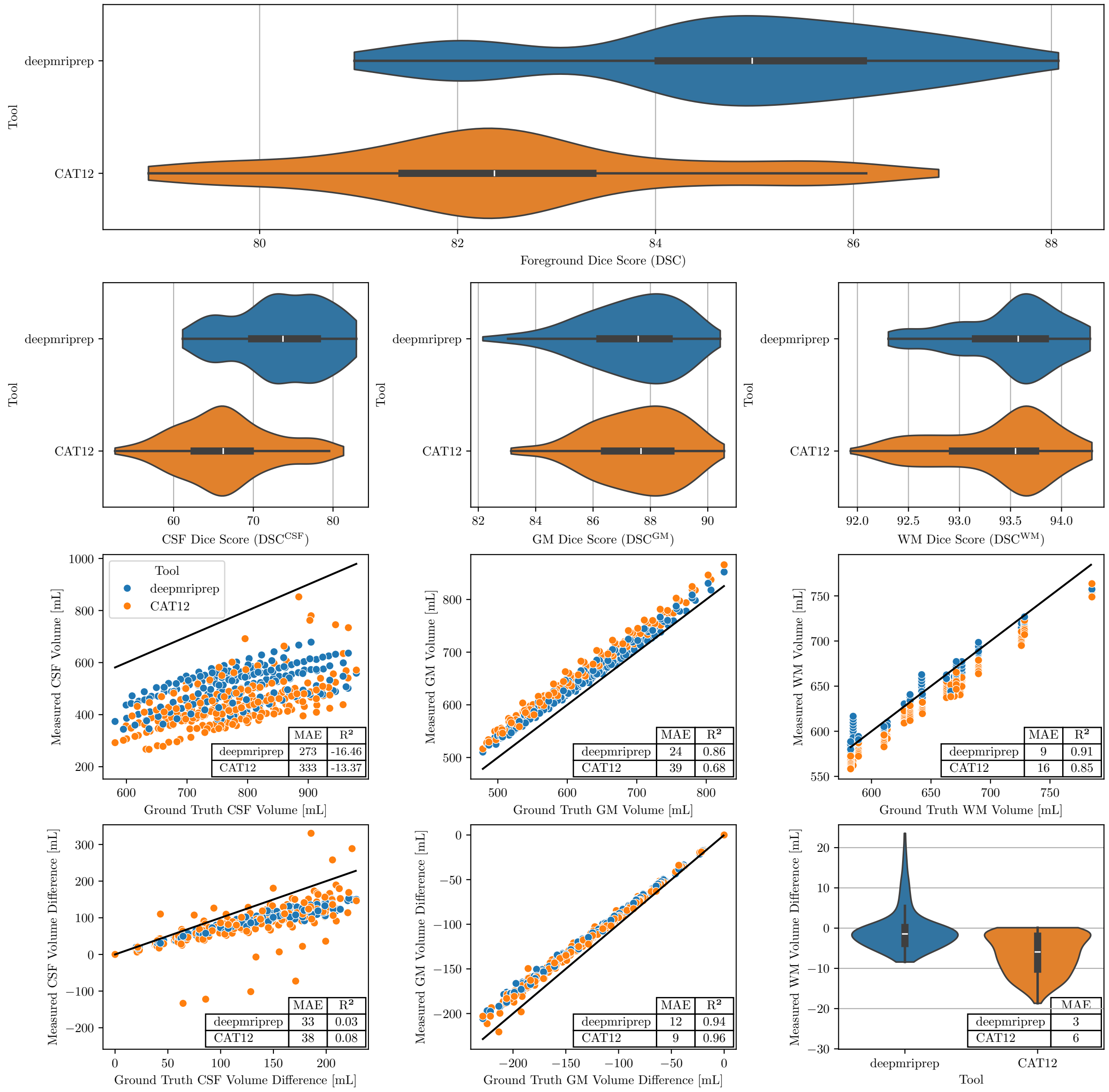

CSF: Cerebrospinal Fluid; GM: Gray Matter; WM: White Matter

Supplementary Figure 34: Top: Dice scores of deepmriprep and CAT12 across 220 images from the Synthetic Atrophy dataset with respect to the cerebrospinal fluid (CSF), gray matter (GM), white matter (WM), and foreground (mean of CSF, GM and WM). Bottom: Measured tissue volumes and tissue volume differences (MR image with synthetic atrophy vs. respective original MR image). Since the synthetic atrophy did not affect WM, the respective ground truth volume difference is always zero, hence R^2 is not reported for WM volume differences. Violin plot density traces terminate exactly at the observed minima and maxima and the superimposed box plots represent 25th percentile (lower), median (center), and 75th percentile (upper) with whiskers extending to data points within 1.5 × IQR of the quartiles.

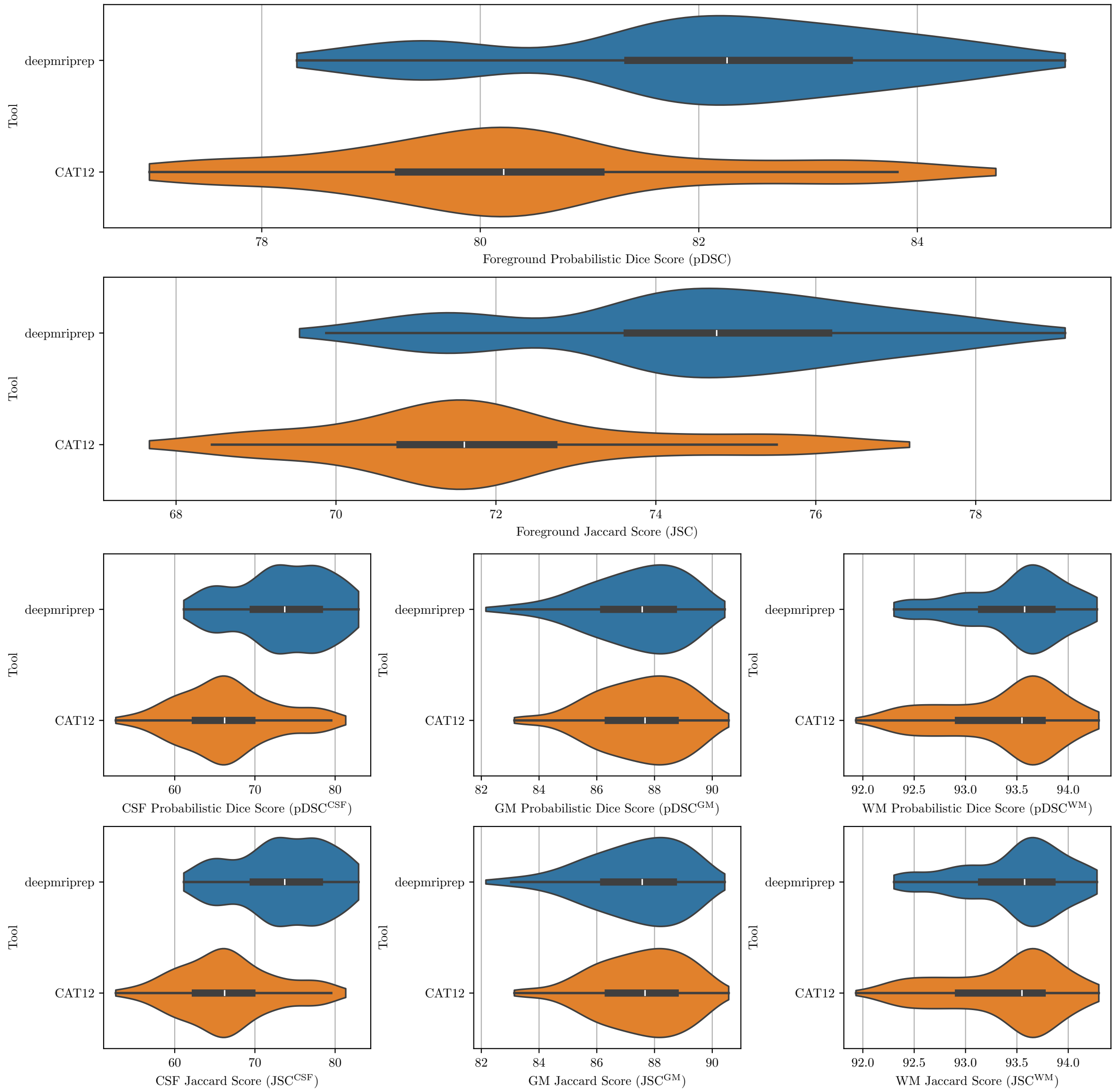

CSF: Cerebrospinal Fluid; GM: Gray Matter; WM: White Matter

Supplementary Figure 35: Probabilistic dice scores and Jaccard score of deepmriprep and CAT12 across 220 images from the Synthetic Atrophy dataset with respect to the cerebrospinal fluid (CSF), gray matter (GM), white matter (WM), and foreground (mean of CSF, GM and WM). Violin plot density traces terminate exactly at the observed minima and maxima and the superimposed box plots represent 25th percentile (lower), median (center), and 75th percentile (upper) with whiskers extending to data points within 1.5 × IQR of the quartiles.

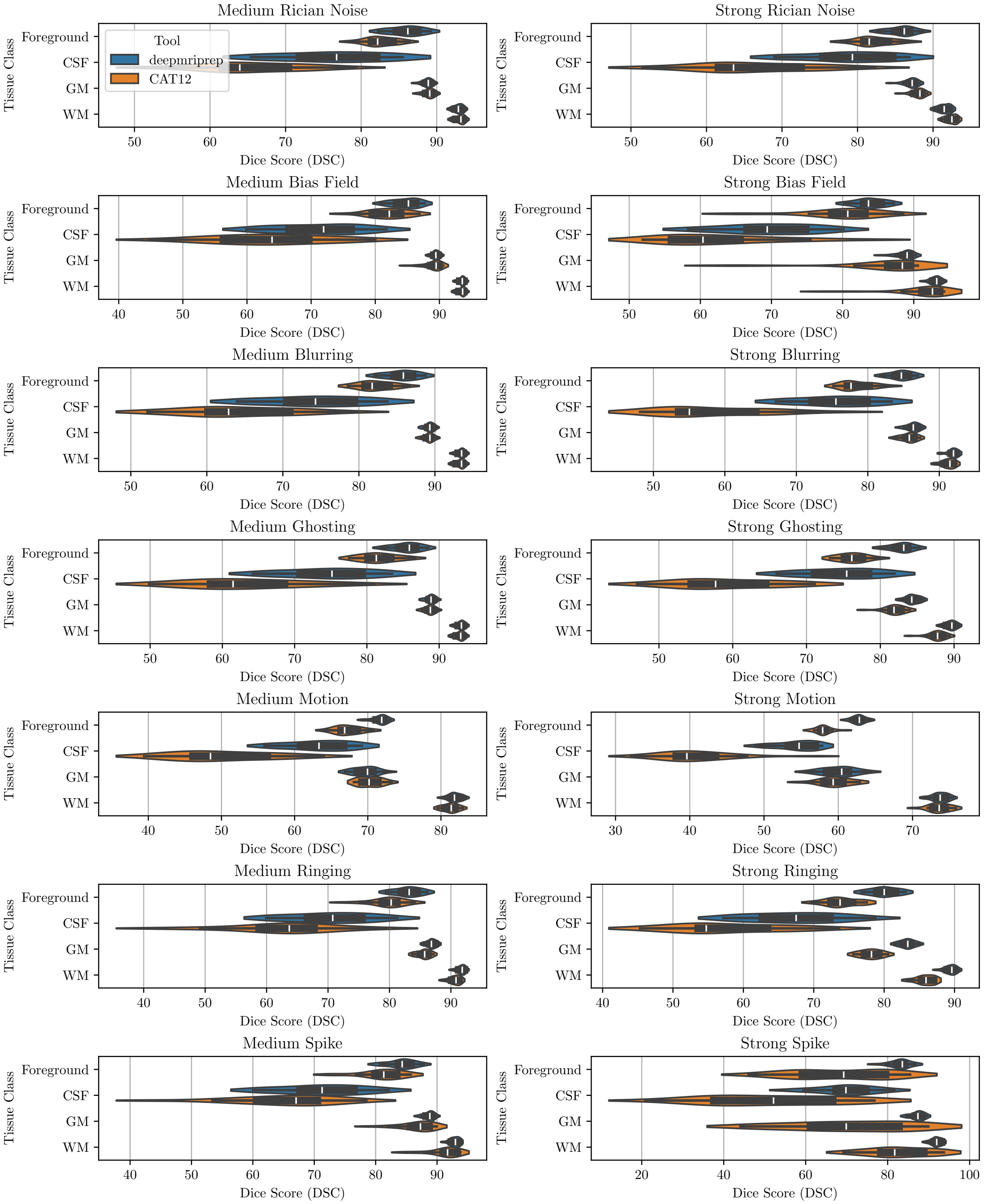

CSF: Cerebrospinal Fluid; GM: Gray Matter; WM: White Matter

Supplementary Figure 36: Dice scores of deepmriprep and CAT12 across 20 original images from the Synthetic Atrophy dataset with medium (left) and strong (right) synthetic image artifacts. Violin plot density traces terminate exactly at the observed minima and maxima and the superimposed box plots represent 25th percentile (lower), median (center), and 75th percentile (upper) with whiskers extending to data points within 1.5 × IQR of the quartiles.

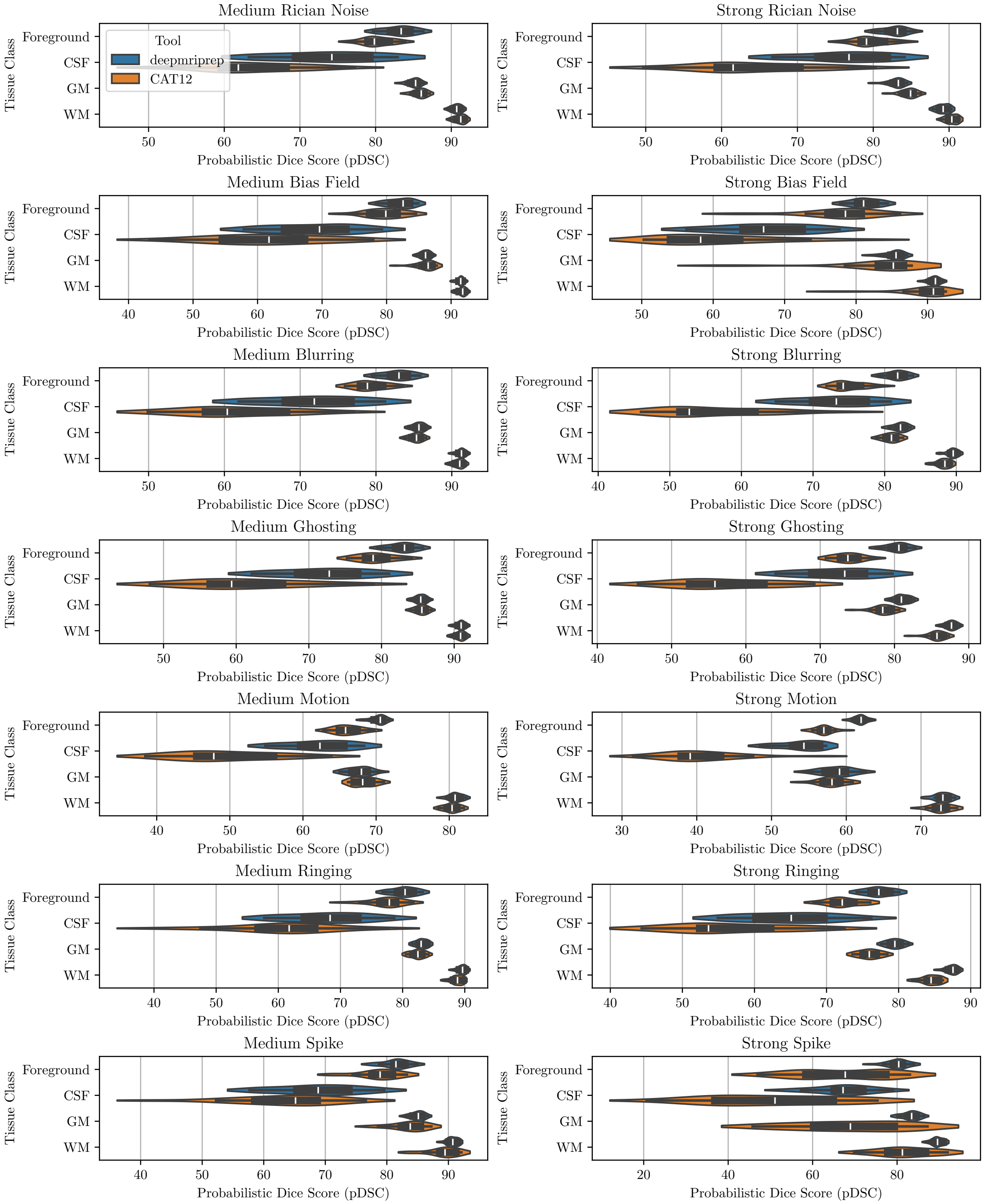

CSF: Cerebrospinal Fluid; GM: Gray Matter; WM: White Matter

Supplementary Figure 37: Probabilistic Dice scores of deepmriprep and CAT12 across 20 original images from the Synthetic Atrophy dataset with medium (left) and strong (right) synthetic image artifacts. Violin plot density traces terminate exactly at the observed minima and maxima and the superimposed box plots represent 25th percentile (lower), median (center), and 75th percentile (upper) with whiskers extending to data points within 1.5 × IQR of the quartiles.

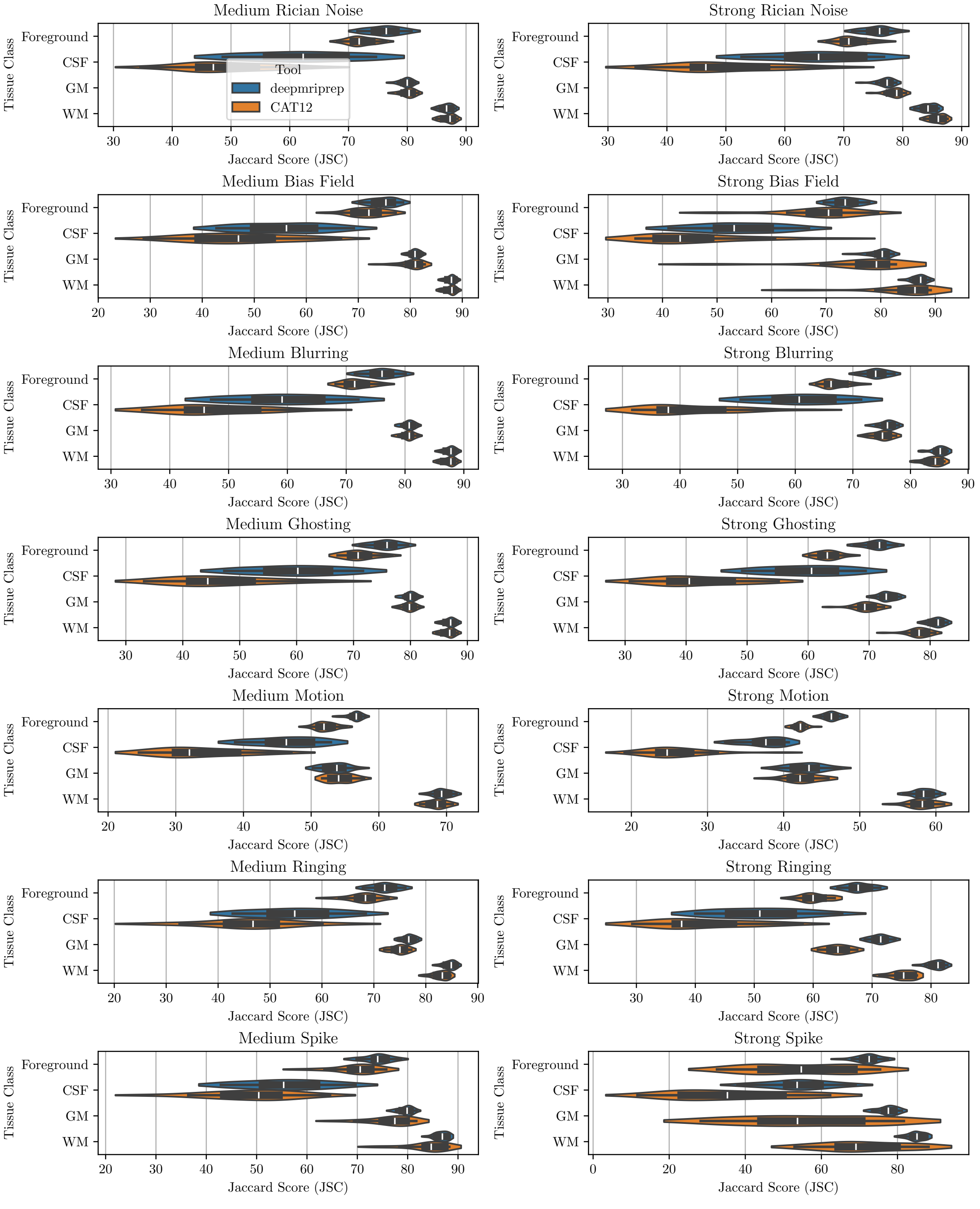

CSF: Cerebrospinal Fluid; GM: Gray Matter; WM: White Matter

Supplementary Figure 38: Jaccard scores of deepmriprep and CAT12 across 20 original images from the Synthetic Atrophy dataset with medium (left) and strong (right) synthetic image artifacts. Violin plot density traces terminate exactly at the observed minima and maxima and the superimposed box plots represent 25th percentile (lower), median (center), and 75th percentile (upper) with whiskers extending to data points within 1.5 $\times$ IQR of the quartiles.

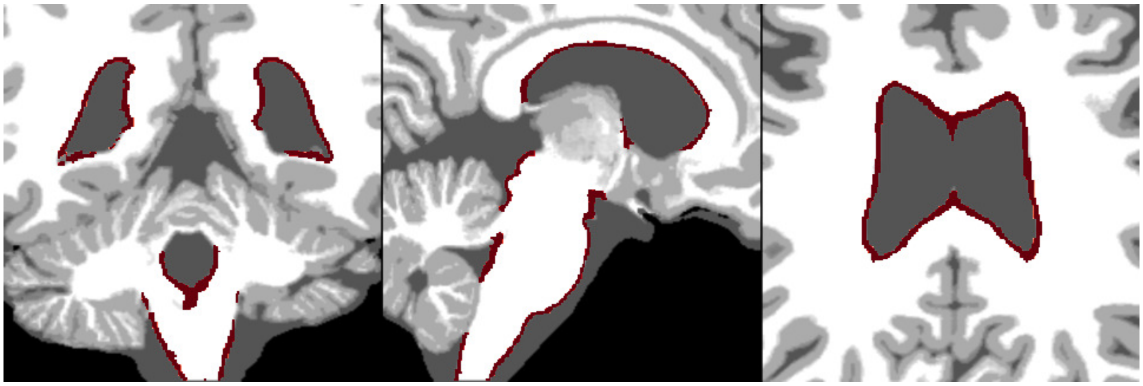

Supplementary Figure 39: Coronal (left), sagittal (center), and axial slice (right) of an example tissue segmentation map, with all voxels subject to gray matter (GM) masking highlighted in red.

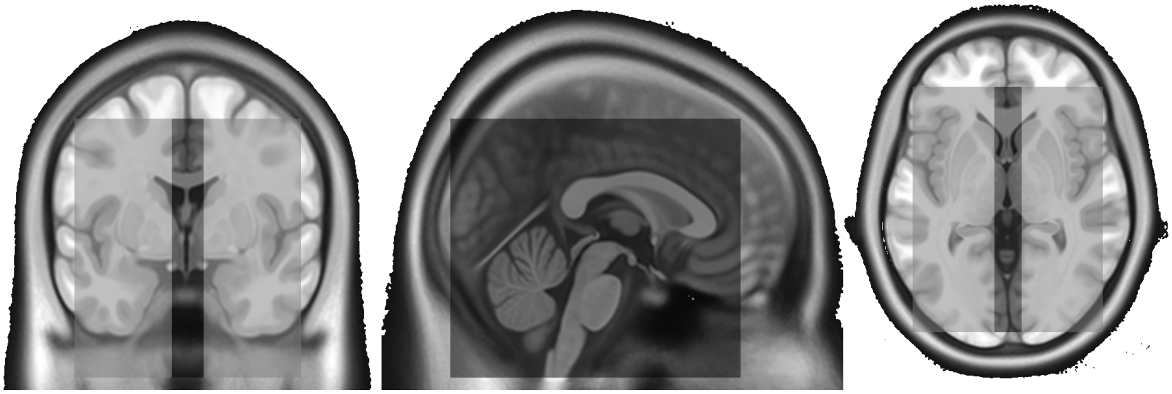

Supplementary Figure 40: Coronal (left), sagittal (center), and axial slice (right) of the two 128x228x256 voxel patches used for gray matter masking. For reference, the T1 template of CAT12, upsampled to the utilized resolution of 0.5 mm, is shown in the background

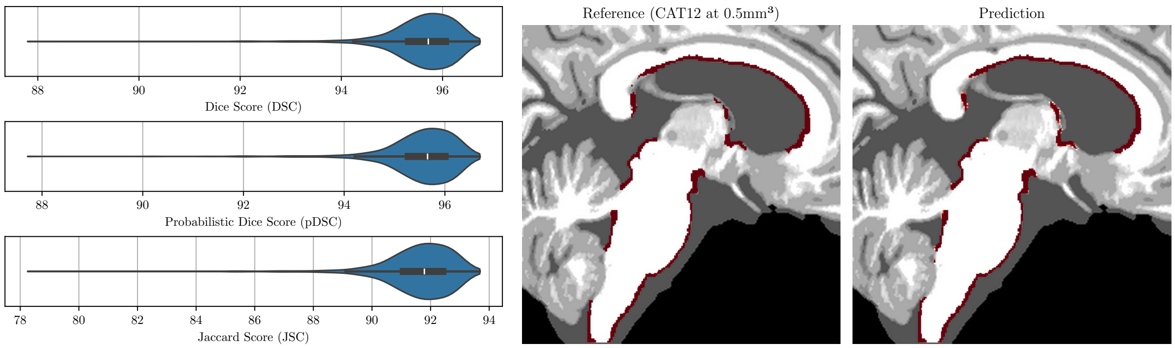

Supplementary Figure 41: Left: Distribution of Dice scores probabilistic Dice scores, and Jaccard scores of the predicted gray matter (GM) masks across 685 validation images obtained during five-fold cross-dataset validation. Violin plot density traces terminate exactly at the observed minima and maxima and the superimposed box plots represent 25th percentile (lower), median (center), and 75th percentile (upper) with whiskers extending to data points within 1.5 $\times$ IQR of the quartiles. Right: Sagittal slice of the predicted GM mask (red), which resulted in the lowest Dice score compared to the reference GM mask (red). The model input, i.e. the respective tissue segmentation map, is shown in the background.
