# Supplementary material for: deepmriprep: voxel-based morphometry preprocessing via deep neural networks
Source: Nat Comput Sci. 2026 Jan 30;6(3):250–9. doi: 10.1038/s43588-026-00953-7 (PMC13021509; doi:10.1038/s43588-026-00953-7)
Supplement: Supplementary file 2 — Reporting Summary [file 43588_2026_953_MOESM2_ESM.pdf]

nature portfolio

Corresponding author(s): Lukas Fisch

Last updated by author(s): Nov 28, 2025

Reporting Summary

Nature Portfolio wishes to improve the reproducibility of the work that we publish. This form provides structure for consistency and transparency in reporting. For further information on Nature Portfolio policies, see our Editorial Policies and the Editorial Policy Checklist.

Statistics

For all statistical analyses, confirm that the following items are present in the figure legend, table legend, main text, or Methods section.

n/a	Confirmed	
☐	☒	The exact sample size (*n*) for each experimental group/condition, given as a discrete number and unit of measurement
☐	☒	A statement on whether measurements were taken from distinct samples or whether the same sample was measured repeatedly
☐	☒	The statistical test(s) used AND whether they are one- or two-sided<br>*Only common tests should be described solely by name; describe more complex techniques in the Methods section.*
☐	☒	A description of all covariates tested
☐	☒	A description of any assumptions or corrections, such as tests of normality and adjustment for multiple comparisons
☐	☒	A full description of the statistical parameters including central tendency (e.g. means) or other basic estimates (e.g. regression coefficient) AND variation (e.g. standard deviation) or associated estimates of uncertainty (e.g. confidence intervals)
☐	☒	For null hypothesis testing, the test statistic (e.g. *F*, *t*, *r*) with confidence intervals, effect sizes, degrees of freedom and *P* value noted<br>*Give P values as exact values whenever suitable.*
☒	☐	For Bayesian analysis, information on the choice of priors and Markov chain Monte Carlo settings
☒	☐	For hierarchical and complex designs, identification of the appropriate level for tests and full reporting of outcomes
☐	☒	Estimates of effect sizes (e.g. Cohen's *d*, Pearson's *r*), indicating how they were calculated

Our web collection on statistics for biologists contains articles on many of the points above.

Software and code

Policy information about availability of computer code

Data collection	openneuro-py version 2023.1.0 (Python package: https://pypi.org/project/openneuro-py) was used to download the OpenNeuro datasets.
Data analysis	The implementation of the models and the respective training procedures are publicly accessible at https://github.com/wwu-mmll/deepmriprep-train which uses the niftiai version 0.3.2 Python package (https://pypi.org/project/niftiai). Besides deepmriprep version 0.3.1 Python package (https://pypi.org/project/deepmriprep), CAT12 version 12.8.2 (https://neuro-jena.github.io/cat/) was used for preprocessing and nilearn version 0.10.1 (https://pypi.org/project/nilearn) was used for VBM analyses.

For manuscripts utilizing custom algorithms or software that are central to the research but not yet described in published literature, software must be made available to editors and reviewers. We strongly encourage code deposition in a community repository (e.g. GitHub). See the Nature Portfolio guidelines for submitting code & software for further information.

Data

Policy information about availability of data

All manuscripts must include a data availability statement. This statement should provide the following information, where applicable:

- Accession codes, unique identifiers, or web links for publicly available datasets
- A description of any restrictions on data availability
- For clinical datasets or third party data, please ensure that the statement adheres to our policy

All raw data of the datasets OpenNeuro-HD, OpenNeuro-Total and OpenNeuro-Kids is publicly available at https://openneuro.org (the respective OpenNeuro

dataset IDs and filepaths can be found in the Supplementary Data).
All raw data of the Synthetic Atrophy dataset is publicly available at https://data.csiro.au/collection/csiro:53241.
With regard to the Marburg-Münster Affective Disorders Cohort Study (FOR2107/MACS), Münster Neuroimaging Cohort (MNC) and BiDirect dataset, individual raw data is not published due to current EU data protection regulations and the sensitive nature of clinical MRI data but can be made available in form of summary statistics or anonymized aggregation of voxel-wise data upon reasonable request to the corresponding author, within four weeks, depending on the required data or results derivates. Source data are provided in this paper. The data availability of the FOR2107/MACS and MNC dataset is governed by Udo Dannlowski and the availibility of the BiDirect dataset is governed by Klaus Berger.

Research involving human participants, their data, or biological material

Policy information about studies with human participants or human data. See also policy information about sex, gender (identity/presentation), and sexual orientation and race, ethnicity and racism.

Reporting on sex and gender	Gender was not considered and sex was determined based on self-reporting. To control for confounding effects, VBM analyses used sex as a covariate.
Reporting on race, ethnicity, or other socially relevant groupings	Race or ethnicity were not considered.
Population characteristics	Described in the methods section.
Recruitment	Described in the methods section.
Ethics oversight	Described in the methods section.

Note that full information on the approval of the study protocol must also be provided in the manuscript.

Field-specific reporting

Please select the one below that is the best fit for your research. If you are not sure, read the appropriate sections before making your selection.

☒ Life sciences ☐ Behavioural & social sciences ☐ Ecological, evolutionary & environmental sciences

For a reference copy of the document with all sections, see nature.com/documents/nr-reporting-summary-flat.pdf

Life sciences study design

All studies must disclose on these points even when the disclosure is negative.

Sample size	On top of Synthetic Atrophy dataset, the Marburg-Münster Affective Disorders Cohort Study (FOR2107/MACS), Münster Neuroimaging Cohort (MNC) and BiDirect dataset, the number of datasets was maximized by crawling MRI data available at OpenNeuro, yielding 225 OpenNeuro datasets which should be sufficient.
Data exclusions	29 MRIs were excluded from OpenNeuro-Total due to improper masking and erroneous orientation and 300 MRIs were excluded from OpenNeuro-Kids due to improper masking and strong motion artifacts. Additionally, images in OpenNeuro-HD had to meet strict quality thresholds (minimum B- grade) to be included.
Replication	Best practices were applied: 1. Grouped cross-validation to ensure that validation images came from datasets unseen during training 2. Usage of multiple separate test datasets 3. VBM analyses were repeated 100 times with a randomly picked 80% subset.
Randomization	VBM analyses used sex and age as covariates.
Blinding	Given the retrospective nature of the study blinding is not relevant.

Reporting for specific materials, systems and methods

We require information from authors about some types of materials, experimental systems and methods used in many studies. Here, indicate whether each material, system or method listed is relevant to your study. If you are not sure if a list item applies to your research, read the appropriate section before selecting a response.

Materials & experimental systems

n/a	Involved in the study
☒	☐ Antibodies
☒	☐ Eukaryotic cell lines
☒	☐ Palaeontology and archaeology
☒	☐ Animals and other organisms
☒	☐ Clinical data
☒	☐ Dual use research of concern
☒	☐ Plants

Methods

n/a	Involved in the study
☒	☐ ChIP-seq
☒	☐ Flow cytometry
☐	☒ MRI-based neuroimaging

Plants

Seed stocks: n/a

Novel plant genotypes: n/a

Authentication: n/a

Magnetic resonance imaging

Experimental design

Design type: Structural T1-weighted MRI

Design specifications: n/a

Behavioral performance measures: n/a

Acquisition

Imaging type(s): Structural

Field strength: OpenNeuro datasets contain 1.5, 3 and 7 Tesla MRIs while test datasets were all measured with 3 Tesla.

Sequence & imaging parameters: Sequence details can be found in the respective dataset sources.

Area of acquisition: Whole-brain scan.

Diffusion MRI: ☐ Used ☒ Not used

Preprocessing

Preprocessing software: CAT12 version 12.8.2 (https://neuro-jena.github.io/cat/) and deepmriprep version 0.3.1 (Python package: https://pypi.org/project/deepmriprep).

Normalization: Images were non-linearly spatially normalized as described in the methods section.

Normalization template: MNI template of CAT12.

Noise and artifact removal: CAT12 uses a spatial adaptive non-local means (SANLM) denoising filter while deepmriprep uses a 3D CNN explicitly trained to be robust with respect to any MRI artifacts via data augmentation.

Volume censoring: SPM is used for volume censoring of non-brain voxels in CAT12 while deepmriprep uses deepbet version 1.0.2 (https://pypi.org/project/deepbet).

Statistical modeling & inference

Model type and settings: Voxel-Based Morphometry (VBM); analyses included associations with biological (age, sex, BMI) and psychological (MDD vs

Model type and settings	HC, IQ, Years of Education) variables. Bootstrapping was employed (N=100 repetitions with 80% subsets) to ensure robust inference.
Effect(s) tested	The study investigates the effects of preprocessing on Voxel-Based Morphometry (VBM) findings, analyzing associations with biological (age, sex, BMI) and psychological (Years of Education, Major Depression Disorder vs. Healthy Controls, IQ) variables using generalized linear modeling (GLM). Bootstrapping (N=100 iterations) ensures reliability.

Specify type of analysis: ☒ Whole brain ☐ ROI-based ☐ Both

Statistic type for inference<br>(See Eklund et al. 2016)	Voxel-wise
Correction	t-maps were liberaly thresholded at $p < 0.001$ to compare effects obtained with CAT12 and deepmriprep without the influence of additional corrections.

Models & analysis

n/a	Involved in the study
☒	☐ Functional and/or effective connectivity
☒	☐ Graph analysis
☐	☒ Multivariate modeling or predictive analysis

Multivariate modeling and predictive analysis	Independent Variables: Age, sex, BMI, education, MDD vs. HC, IQ.<br>Feature Extraction & Dimension Reduction: VBM on T1-weighted MRI, preprocessing with deepmriprep and CAT12.<br>Model: deepmriprep (neural network) vs. CAT12.<br>Training & Validation Metrics: Dice Score for segmentation and MSE and Linear Elasticity for registration.<br>Evaluation Metrics: Bootstrapped VBM (100 iterations, 80% subsets), correlation with CAT12, variable associations.
